# Stop codon read-through of mammalian *MTCH2* leading to an unstable isoform regulates mitochondrial membrane potential

**DOI:** 10.1074/jbc.RA120.014253

**Published:** 2020-10-07

**Authors:** Lekha E. Manjunath, Anumeha Singh, Sarthak Sahoo, Ashutosh Mishra, Jinsha Padmarajan, Chaithanya G. Basavaraju, Sandeep M. Eswarappa

**Affiliations:** Department of Biochemistry, Indian Institute of Science, Bengaluru, Karnataka, India

**Keywords:** mitochondria, MTCH2, stop codon, translational read-through, translation control, mRNA, protein degradation, mitochondrial membrane potential, ribosome, stop, codon

## Abstract

Stop codon read-through (SCR) is a process of continuation of translation beyond a stop codon. This phenomenon, which occurs only in certain mRNAs under specific conditions, leads to a longer isoform with properties different from that of the canonical isoform. *MTCH2*, which encodes a mitochondrial protein that regulates mitochondrial metabolism, was selected as a potential read-through candidate based on evolutionary conservation observed in the proximal region of its 3′ UTR. Here, we demonstrate translational read-through across two evolutionarily conserved, in-frame stop codons of *MTCH2* using luminescence- and fluorescence-based assays, and by analyzing ribosome-profiling and mass spectrometry (MS) data. This phenomenon generates two isoforms, MTCH2x and MTCH2xx (single- and double-SCR products, respectively), in addition to the canonical isoform MTCH2, from the same mRNA. Our experiments revealed that a *cis*-acting 12-nucleotide sequence in the proximal 3′ UTR of *MTCH2* is the necessary signal for SCR. Functional characterization showed that MTCH2 and MTCH2x were localized to mitochondria with a long *t*_1/2_ (>36 h). However, MTCH2xx was found predominantly in the cytoplasm. This mislocalization and its unique C terminus led to increased degradation, as shown by greatly reduced *t*_1/2_ (<1 h). *MTCH2* read-through–deficient cells, generated using CRISPR-Cas9, showed increased MTCH2 expression and, consistent with this, decreased mitochondrial membrane potential. Thus, double-SCR of *MTCH2* regulates its own expression levels contributing toward the maintenance of normal mitochondrial membrane potential.

Stop codon read-through (SCR), also known as translational read-through, is the process in which ribosomes continue to translate beyond the canonical stop codon, up to a downstream, in-frame stop codon, resulting in a polypeptide with a C-terminal extension. The basal levels of SCR, which occur due to errors during translation termination are very low (0.01 to 0.1% in mammalian cells) as the termination machinery is very accurate (1). However, in certain transcripts, SCR is induced by *cis*-acting RNA element or *trans*-acting factors interacting with the element (2–4). Efficiency of read-through is much higher than the basal level in case of these programmed events.

SCR was first described in viruses and has subsequently been extensively studied (5). Read-through has also been reported in fungi, protozoans, and insects (6–10). Translational read-through in mammals has been shown in the following genes: *HBB, MPZ, VEGFA, AGO1, OPRL1, OPRK1, AQP4, MAPK10, LDHB, MDH1, AMD1,* and *VDR* (2, 3, 11–18). Comparative genomics analysis and ribosomal profiling studies have not only identified candidate genes for SCR, they have also suggested potential double-SCR (read-through across two in-frame stop codons) candidate genes in *Drosophila* and *Plasmodium* (6, 8, 9). Double-SCR was demonstrated in case of carnation mottle virus (CarMV) *in vitro* using rabbit reticulocyte lysate (19). However, to date, double-SCR has not been experimentally demonstrated in any living system.

SCR contributes to proteome expansion and adds to functional diversity. In some cases, this process generates proteins with different cellular localization. *e.g. LDHB* and *MDH1* (14, 17). The read-through protein can also have a function different from that of the canonical protein as seen in *VEGFA* and *AGO1,* or different efficiency as seen in *VDR* (2, 3, 15, 20). The process of read-through has also been reported to limit the protein synthesis from the mRNA by causing ribosomal stalling at the downstream stop codon (16). Recent studies have revealed the *in vivo* physiological significance of SCR. AQP4ex^−/−^ mouse, which lacks the read-through product of *AQP4*, shows altered localization and increased expression of AQP4, the canonical isoform. This study also suggests that AQP4ex plays a role in neuromyelitis optica (21). Up-regulation of SCR product of *MPZ* in mice leads to a neuropathy that resembles Charcot–Marie–Tooth disease (22).

One of the genes identified as a potential SCR candidate in a genome-wide, bioinformatics-based screen was *MTCH2*, a gene present in the nuclear genome encoding a mitochondrial protein (2). MTCH2 is a ∼33 kDa outer mitochondrial membrane protein, which is homologous to mitochondrial carrier proteins. Genome-wide association studies have linked *MTCH2* with obesity, diabetes, and Alzheimer's disease (23–25). The role of MTCH2 in the regulation of lipid homeostasis has been shown in zebrafish, *Caenorhabditis elegans,* and mouse (26–28). MTCH2 plays a key role in mitochondrial pathway of apoptosis, mitochondrial metabolism, and mitochondrial fusion. MTCH2 aids in the recruitment of the pro-apoptotic tBID to mitochondria and has a critical function in Fas-induced liver apoptosis (29). *MTCH2* deletion results in embryonic lethality in mice. Skeletal muscle-specific deletions of *MTCH2* in mice results in increased mitochondrial mass and metabolism granting protection against diet-induced obesity (30). In this study, we demonstrate double-SCR of *MTCH2*. We identify a *cis*-acting RNA signal element that drives the double-SCR. This process regulates the expression of *MTCH2* by generating a highly unstable isoform with cytoplasmic localization. Using *MTCH2* read-through–deficient cells, generated by CRISPR-Cas9 system, we show that this process is vital for the maintenance of the mitochondrial membrane potential.

## Results

### Evolutionary conservation at the proximal 3′ UTR of MTCH2

**Figure 1.**
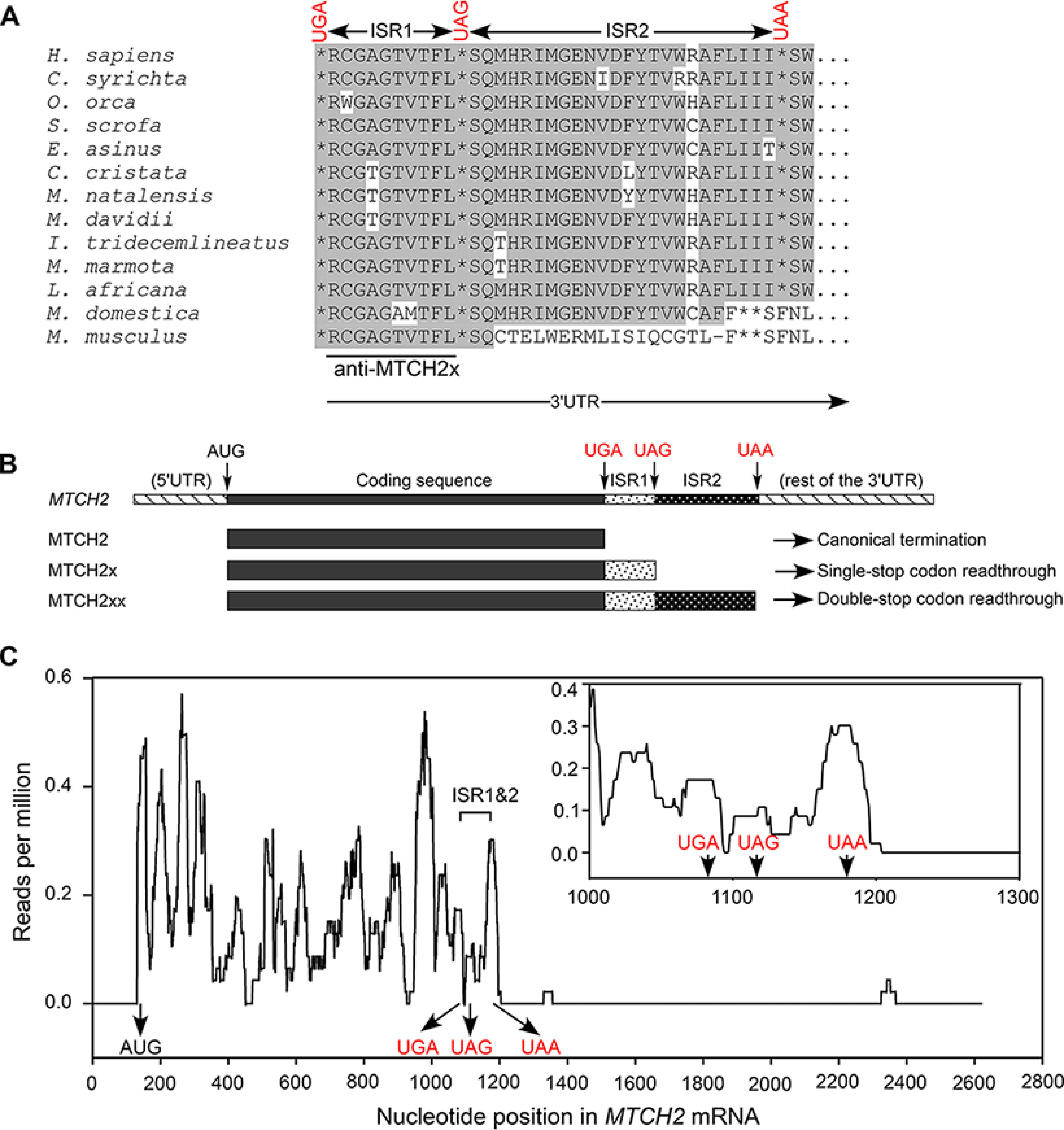
**Mammalian *MTCH2* has potential to undergo double stop codon read-through during translation.** *A,* alignment of amino acid sequences potentially encoded in the proximal 3' UTR of *MTCH2* from multiple mammalian species. Position of the canonical stop codon (UGA), first (UAG), and second (UAA) downstream in-frame stop codons are shown. Two inter stop codon regions (*ISR1* and *ISR2*) are indicated. Peptide used to raise a specific antibody is also shown. Conserved amino acids are shown in *gray* background. *B,* schematic of three potential MTCH2 isoforms generated from the same mRNA depending on the site of translation termination. *C, MTCH2* ribosome profile in mouse liver (SRR1630813) showing the presence of ribosomes in ISR1 and ISR2. Position of start codon and three in-frame stop codons are shown. A zoomed in portion near the stop codons is shown in the *inset*.

In our previous study, we had predicted SCR in *MTCH2* (2). This prediction was based on an evolutionarily conserved stop codon (UAG), 30 nucleotides downstream of the canonical stop codon. Also, the sequence between the two stop codons and their in-frame nature is highly conserved in mammals. Interestingly, the conservation at both amino acid and nucleotide level extends beyond the downstream stop codon in most mammals until another in-frame stop codon, UAA, which is 105 nucleotides downstream of the canonical stop codon (Fig. 1*A* and Fig. S1). This observation suggests a possibility of translational read-through across two stop codons of *MTCH2*, which will be referred to as “double-SCR.” Thus, there could be three isoforms from the same mRNA: translation termination at the first stop codon (UGA) results in the canonical isoform MTCH2; read-through across the first stop codon and termination at the second stop codon (UAG) results in an isoform with 11 extra amino acids at the C terminus (termed MTCH2x); read-through across the first and the second stop codons (double-SCR), and termination at the third stop codon (UAA) results in an isoform with 36 extra amino acids at the C terminus (termed MTCH2xx) (Fig. 1*B*).

### Evidence for double-SCR in ribosome profiling data

To gather more evidence for SCR in *MTCH2*, we analyzed ribosome profiling data available in the Sequence Read Archive (SRA) of NCBI. Ribosome profiling reveals ribosome-bound regions of mRNAs, which indicates translation in that region. Ribosome profiling has been successfully used to predict SCR (8). We analyzed ribosome footprints on *MTCH2* mRNA as described previously (3). 3′ UTR downstream of the two inter-stop codon regions (ISR1 and ISR2) was used as control to know the level of background reads. Interestingly, ribosome footprints were enriched in ISR1 and ISR2 of *MTCH2* mRNA compared with the rest of the 3′ UTR. It is important to note that the sequences of ISR1 and ISR2 are unique to *MTCH2*. No other mammalian gene has sequence similar to these. Therefore, the presence of ribosome footprints in ISR1 and ISR2 strongly indicates double-SCR. Ribosome footprints in ISR1 and ISR2 of *MTCH2* were detected in liver, kidney, primary keratinocytes, embryonic stem cells, neural stem cells, and bone marrow-derived dendritic cells (Fig. 1*C* and Fig. S2). The relative density of the ribosomal footprints in ISR1 and ISR2 varies among different cell types suggesting cell/tissue-specific regulation. These observations provided more evidence for double-SCR in *MTCH2*.

**Figure 2.**
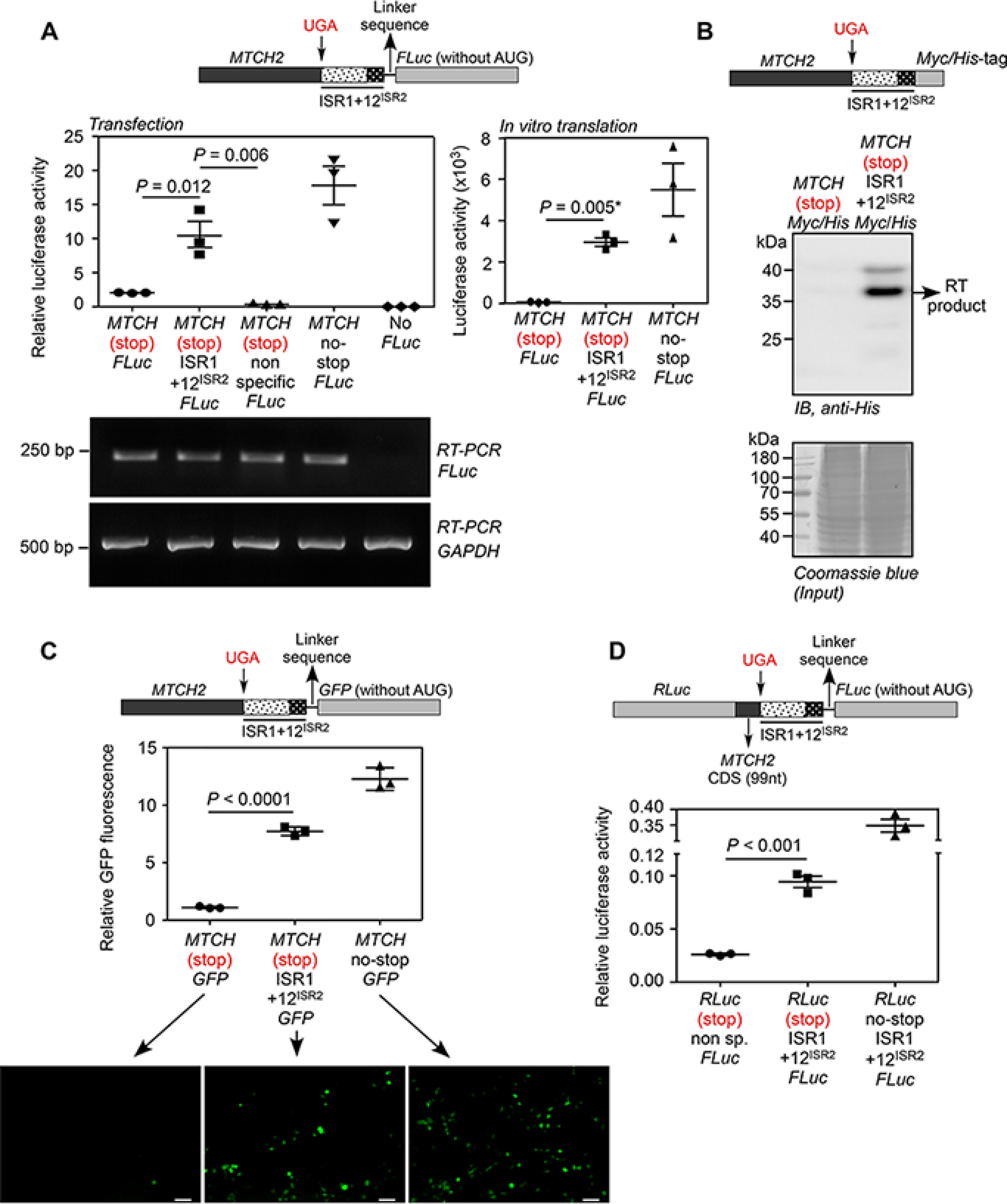
**Demonstration of translational read-through across the canonical stop codon of *MTCH2*.** *A,* luminescence-based assay: plasmid expressing in-frame MTCH2-(UGA)-(ISR1 + 12^ISR2^)-FLuc and its variants were transfected in HEK293 cells. FLuc activity relative to the activity of co-transfected *Renilla* luciferase (RLuc) is shown (*left*). A construct without ISR1 + 12^ISR2^ between *MTCH2* and *FLuc*, and another construct with a nonspecific sequence between them served as negative controls. RT-PCR of *FLuc* mRNA is shown. The constructs were subjected to *in vitro* transcription and *in vitro* translation using rabbit reticulocyte lysate. Luciferase activity is shown (*right*). *B,* Western blotting-based assay: HEK293 cells were transfected with MTCH2-(UGA)-(ISR1 + 12^ISR2^)-Myc/His construct. His-tagged translational read-through product was enriched using Ni-NTA column and was detected by Western blotting using anti-His antibody. *RT*, read-through. *C,* fluorescence-based assay: HEK293 cells were transfected with constructs similar to the one described in *A*, but the coding sequence of FLuc was replaced with GFP. Fluorescence was analyzed by flow cytometry (graph) and fluorescence microcopy (images below). *Scale bar*, 50 μm. Graph shows the mean intensity of GFP fluorescence relative to that of co-transfected RFP fluorescence. *D,* dual luciferase-based assay: 99 nucleotides from the 3′ end of the *MTCH2* coding sequence and ISR1 + 12^ISR2^ were cloned between the coding sequences of RLuc and FLuc (see schematic). All were in-frame. This construct was transfected in HEK293 cells and FLuc activity relative to RLuc is shown. All graphs show mean ± S.E. (*n* = 3). Results are representatives of at least three independent experiments done in triplicate. Two-tailed Student's *t* test was used to calculate the *p* value. *, Welch's correction was applied. *MTCH*, MTCH2; *12^ISR2^*, first 12 nucleotides of ISR2 (UCCCAGAUGCAC); *CDS*, coding sequence.

### Single-SCR in MTCH2

Because double-SCR implies single-SCR, we first investigated read-through across the canonical stop codon (UGA). We employed an established luciferase-based read-through assay for this (2, 3). We cloned the *MTCH2* coding sequence upstream of the firefly luciferase (FLuc) coding sequence along with the first 45 nucleotides of the 3′ UTR (ISR1 and first 12 nucleotides from ISR2 (12^ISR2^)). *MTCH2* and *FLuc* were in-frame. The second stop codon (UAG) was removed such that read-through across the first stop codon generates luciferase enzyme whose activity can be quantified (schematic in Fig. 2*A*). A construct without any ISR and another construct where a scrambled sequence of the same length was inserted in place of ISR1 + 12^ISR2^ served as negative controls. Read-through efficiency was calculated using a construct without any stop codon between *MTCH2* and *FLuc*. These constructs were transfected in HEK293 cells and read-through activity was quantified as FLuc activity relative to the co-transfected *Renilla* luciferase (RLuc) activity. Interestingly, the construct with ISR1 + 12^ISR2^ showed a significant luciferase activity much above the background activity seen in negative controls. The efficiency of read-through in this assay was 59 ± 10% (Fig. 2*A*, *left*). The construct with only ISR1 (without 12^ISR2^) exhibited just about 5% read-through indicating the necessity of 12^ISR2^ in this process (Fig. S3). Because we used an exogenous strong overexpression system in these assays, even the negative control showed higher basal read-through than the normal translational error rate. Nevertheless, it was much lower than the *MTCH2* read-through efficiency in the presence of ISR1 + 12^ISR2^. RT-PCR analysis showed that all these constructs resulted in a comparable amount of luciferase RNA (Fig. 2*A*). Similar results were obtained by *in vitro* translation using rabbit reticulocyte lysate (Fig. 2*A*, *right*). Furthermore, we could also observe read-through product by Western blotting (Fig. 2*B*).

**Figure 3.**
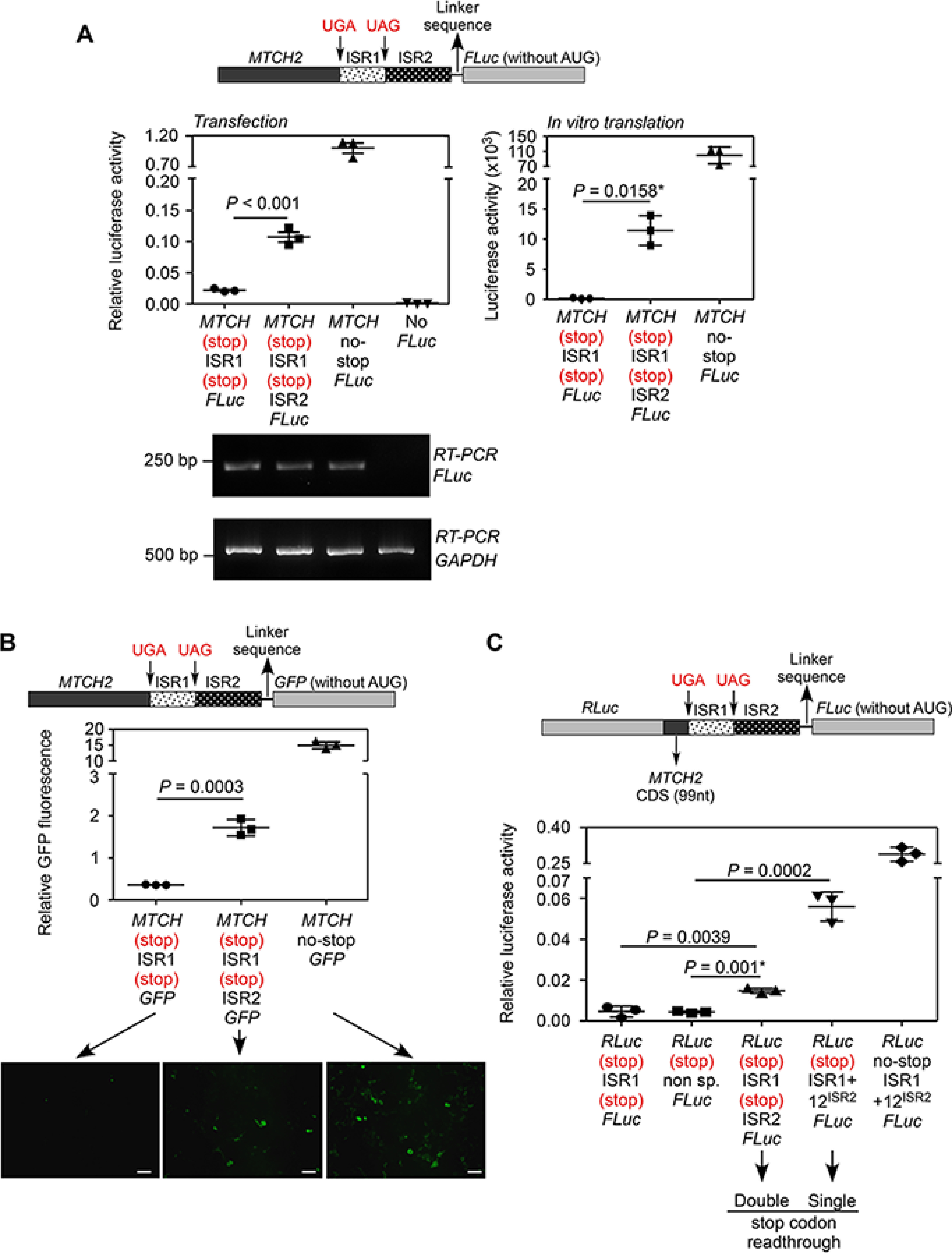
**Demonstration of SCR across the canonical and the first in-frame stop codons of *MTCH2* (double-SCR).** *A,* luminescence-based assay: plasmid expressing in-frame MTCH2-(UGA)-ISR1-(UAG)-ISR2-FLuc and its variants were transfected in HEK293 cells. FLuc activity relative to the activity of co-transfected RLuc is shown (*left*). A construct without ISR2 served as negative control. RT-PCR of *FLuc* mRNA is also shown. The same constructs were subjected to *in vitro* transcription and *in vitro* translation using rabbit reticulocyte lysate. Luciferase activity is shown (*right*). *B,* fluorescence-based assay: HEK293 cells were transfected with constructs similar to the ones described in *A*, but the coding sequence of FLuc was replaced with that of GFP. Fluorescence can be observed only if there is double-SCR. Fluorescence was analyzed by flow cytometry (graph) and by fluorescence microcopy (images below). *Scale bar*, 50 μm. Graph shows the mean intensity of GFP fluorescence relative to that of co-transfected RFP fluorescence. *C,* dual luciferase-based assay: 99 nucleotides from the 3′ end of the *MTCH2* coding sequence, ISR1 and ISR2 were cloned between the coding sequences of RLuc and FLuc (schematic). All were in-frame. Although RLuc activity is expected all the time, FLuc activity is expected only if there is double-SCR. This construct was transfected in HEK293 cells and FLuc activity relative to RLuc is shown. All graphs show mean ± S.D. (*n* = 3). Results are representatives of at least three independent experiments done in triplicate. Two-tailed Student's *t* test was used to calculate the *p* value. *, Welch's correction was applied. *MTCH*, MTCH2; *CDS*, coding sequence.

To further confirm this phenomenon, we replaced the coding sequence of firefly luciferase with that of GFP in all constructs (schematic in Fig. 2*C*). GFP will be translated only if there is read-through across the canonical stop codon of *MTCH2*. When transfected in HEK293 cells, the construct with the ISR1 + 12^ISR2^ region showed fluorescence much above the background level. This observation was made by both fluorescence microscopy and flow cytometry. Efficiency of SCR was 63 ± 3.1% based on flow cytometry analysis (Fig. 2*C*).

To determine whether ISR1 + 12^ISR2^ could drive SCR in a heterologous system, dual luciferase-based read-through assay was carried out. We cloned the ISR1 + 12^ISR2^ region along with distal (3′ end) 99 nucleotides of the coding sequence of *MTCH2* between RLuc and FLuc such that all were in-frame (schematic in Fig. 2*D*). In this construct, FLuc is expressed only if there is SCR. We observed significant FLuc activity in HEK293 cells transfected with the construct containing ISR1 + 12^ISR2^ compared with the construct that lacks this region. Based on the activity of a construct without any stop codon between RLuc and Fluc, the efficiency of single-SCR in this assay was estimated to be 27 ± 1.5% (Fig. 2*D*).

### Double-SCR in MTCH2

We performed the experiments described above to test double-SCR. For luminescence-based assay, we cloned *MTCH2* coding sequence along with ISR1 and ISR2 upstream of and in-frame with the coding sequence of FLuc (schematic in Fig. 3*A*). FLuc activity is expected only if there is translational read-through across both the stop codons (UGA and UAG). A construct without ISR2, but with both the stop codons, served as negative control. When transfected in HEK293 cells, we observed significant FLuc activity compared with the negative control indicating double-SCR. The efficiency of this event was 10.75 ± 0.8%, which was estimated based on another construct without any stop codon between *MTCH2* and FLuc. *In vitro* translation using rabbit reticulocyte lysate also showed comparable results (13.7 ± 3.4%; Fig. 3*A*). Furthermore, we could also demonstrate double-SCR using GFP as reporter instead of FLuc (schematic in Fig. 3*B*). The construct with both ISR1 and ISR2 showed more fluorescence intensity of GFP compared with the negative control demonstrating translational read-through across two stop codons. Efficiency of double-SCR was 11.5 ± 1.1% based on this assay (Fig. 3*B*). Dual luciferase assay also showed double-SCR with an efficiency of 5.15 ± 0.45% (Fig. 3*C*). Notably, the efficiency of double-SCR was much less compared with that of single-SCR in all assays.

### Identification of cis-acting RNA signal sequence required for SCR

**Figure 4.**
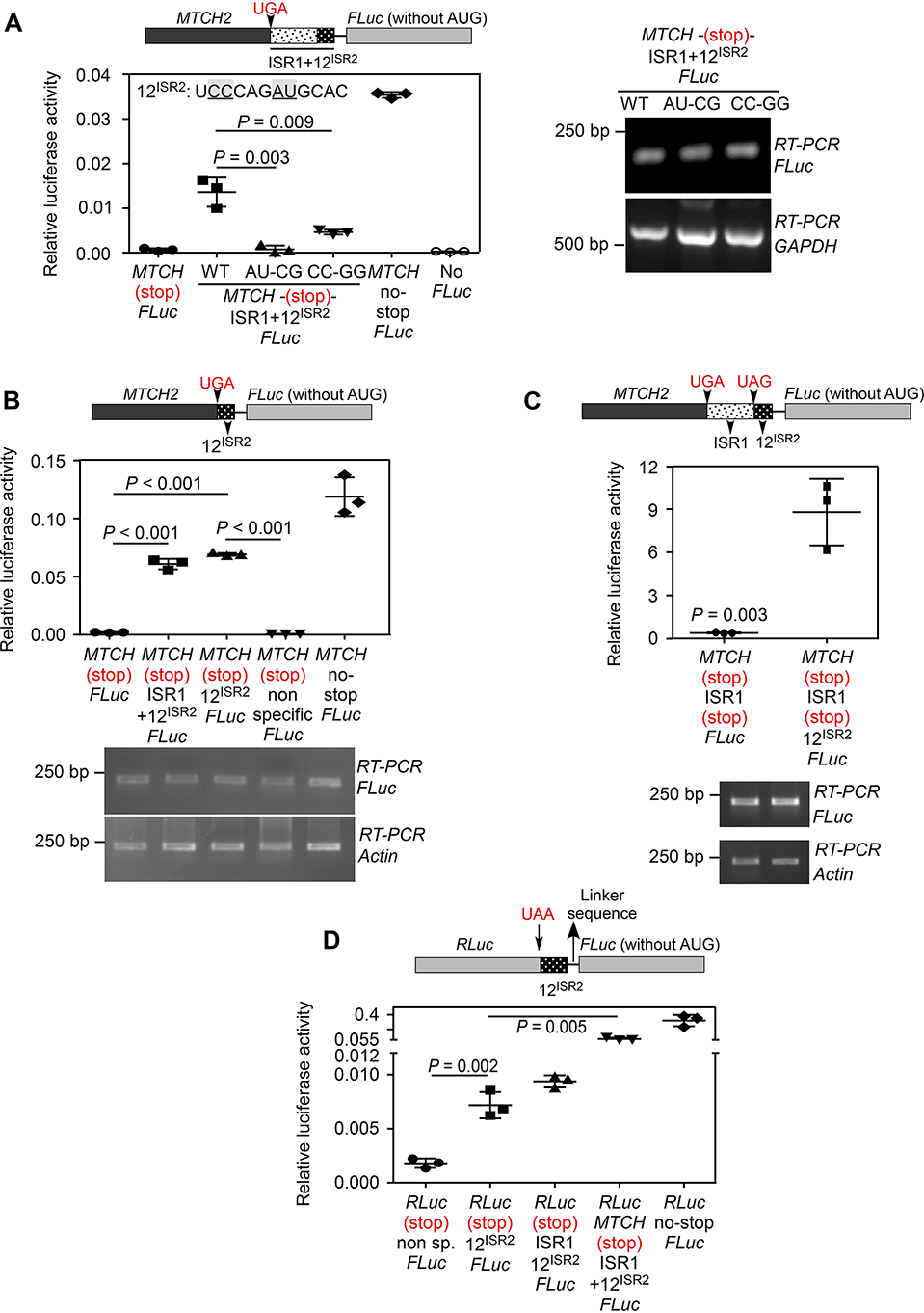
**First 12 nucleotides of ISR2 (12^ISR2^) constitutes the RNA signal necessary for the SCR of *MTCH2*.** *A,* mutations in 12^ISR2^ reduce read-through across the canonical stop codon. Constructs having ISR1 and mutated 12^ISR2^ were transfected in HEK293 cells and luciferase-based read-through assay was performed. Sequence of 12^ISR2^ is shown. Mutated residues are *underlined*. RT-PCR of luciferase mRNA is shown (*right*). *B*, 12^ISR2^ can drive read-through across the canonical stop codon of *MTCH2*. A construct having just 12^ISR2^ between *MTCH2* and *FLuc* was used (schematic). Results of luciferase-based read-through assay carried out in HEK293 cells are shown. *C,* 12^ISR2^ can drive double-SCR of *MTCH2*. Construct having ISR1 + 12^ISR2^ along with the two stop codons between *MTCH2* and *FLuc* was used (schematic). Results of luciferase-based read-through assay carried out in HEK293 cells are shown. *D,* 12^ISR2^ is sufficient to drive SCR in a heterologous context. 12^ISR2^ sequence was cloned between *RLuc* and *FLuc* such that all were in-frame. FLuc activity is expected only if the 12^ISR2^ sequence drives read-through across the stop codon of *RLuc*. Results of luciferase-based read-through assay carried out in HEK293 cells are shown. All graphs show mean ± S.D. (*n* = 3). Results are representative of at least three independent experiments done in triplicate. Two-tailed Student's *t* test was used to calculate the *p* value. *MTCH*, MTCH2; *12^ISR2^*, first 12 nucleotides of ISR2 (UCCCAGAUGCAC).

To identify the *cis*-acting RNA signal sequence that drives read-through, we made two sets of mutations in 12^ISR2^. This sequence was chosen as it was common in our constructs that demonstrated single- and double-SCR (Figs. 2*A* and 3*A*). Interestingly, constructs having ISR1 and mutated 12^ISR2^ showed reduced translational read-through in luminescence-based assay (Fig. 4*A*). Although the ISR1 + 12^ISR2^ region contains a potential stem-loop secondary structure, mutations that can potentially disrupt the predicted structure did not bring down its ability to induce translational read-through (Fig. S4). Remarkably, 12^ISR2^ alone could induce read-through across the canonical stop codon in *MTCH2* as efficiently as ISR1 + 12^ISR2^. Furthermore, 12^ISR2^ could also drive double-SCR in *MTCH2*. These observations were made in HEK293 cells as well as *in vitro* using rabbit reticulocyte lysate (Fig. 4, *B* and *C*, and Fig. S5, *A* and *B*). These results demonstrate that the 12^ISR2^ sequence drives translational read-through across both stop codons in *MTCH2*. We then tested if this element can drive read-through in a heterologous system. We cloned the 12^ISR2^ sequence between RLuc and FLuc such that all were in-frame. FLuc activity is expected only if the 12^ISR2^ sequence drives read-through across the stop codon of RLuc. When transfected in HEK293 cells, we observed significant FLuc activity much above the background level showing that the 12^ISR2^ is the signal sequence that drives the SCR in *MTCH2*. However, the efficiency was much less compared with the construct containing a part of *MTCH2* coding sequence before the stop codon (Fig. 4*D*). Overall, these results show that double-SCR of *MTCH2* is a programmed event driven primarily by the 12^ISR2^ sequence.

**Figure 5.**
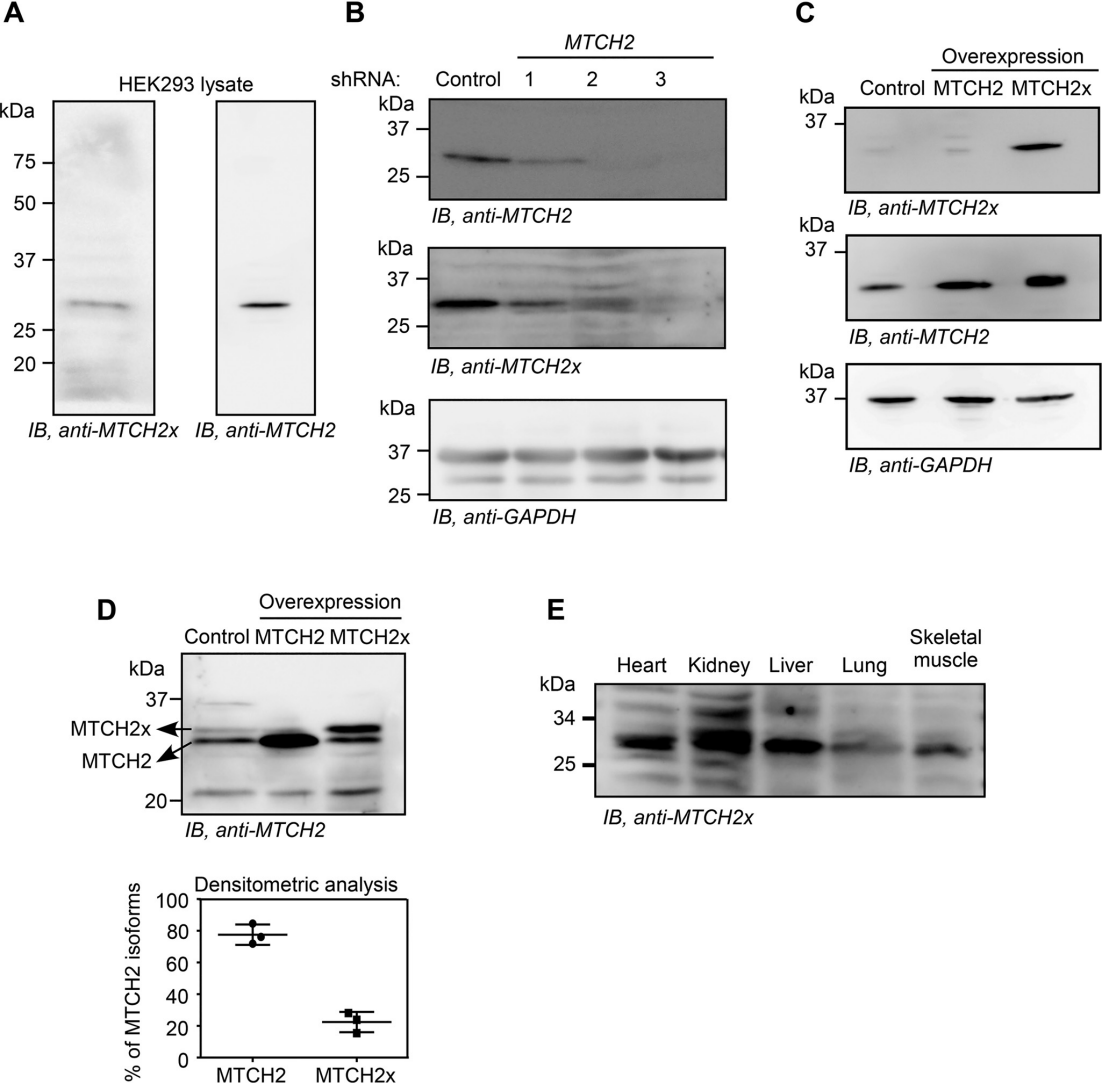
**Detection of endogenous MTCH2x.** *A,* Western blotting performed using an antibody (anti-MTCH2x) generated against the peptide encoded by the ISR1 (RCGAGTVTFL) in HEK293 cell lysate. *B,* Western blotting performed using the same antibody in HEK293 cells transfected with *MTCH2*-specific shRNAs. *C,* detection of exogenous overexpressed MTCH2x by anti-MTCH2x antibody in HEK293 cells. *D,* amount of MTCH2x relative to MTCH2 was estimated by performing a prolonged SDS-PAGE followed by Western blotting using anti-MTCH2 antibody that can potentially recognize all isoforms. Quantification is shown below (mean ± S.D., *n* = 3). Lysates from cells overexpressing MTCH2 or MTCH2x (without any tag) were used to distinguish endogenous MTCH2 and MTCH2x. *E,* detection of endogenous MTCH2x in mitochondrial extracts from multiple mouse organs.

### Detection of endogenous SCR products

To detect endogenous translational read-through products, we first analyzed the available MS data for peptides generated after translational read-through in *MTCH2*. The analyses revealed the presence of read-through–specific peptides in mouse brain and liver. We detected peptides that spanned across both stop codons further indicating double SCR (Table S1). Encouraged by this observation, we raised a polyclonal antibody against the peptide, RCGAGTVTFL, which is generated only after read-through across the canonical stop codon in *MTCH2* (Fig. 1*A*). Because this peptide is predicted to be present in both read-through products, an antibody against it is expected to detect both MTCH2x (34.42 kDa) and MTCH2xx (37.43 kDa) isoforms. We performed Western blotting using this antibody with the lysate from HEK293 cells. The assay detected a distinct band around the expected size of MTCH2x (Fig. 5*A*). The intensity of this band was reduced in cells transfected with *MTCH2*-specific shRNAs, showing that the band indeed represents an isoform of *MTCH2* (Fig. 5*B*). The antibody could also detect overexpressed MTCH2x in HEK293 cells (Fig. 5*C*). A commercially available MTCH2-specific antibody could differentiate MTCH2 (canonical) and MTCH2x isoforms in HEK293 lysates after prolonged electrophoresis. Quantification of the bands revealed that MTCH2x represents about 20% of the total MTCH2 protein under the conditions tested (Fig. 5*D*). Furthermore, endogenous MTCH2x was detected in the mitochondrial extracts from mouse heart, kidney, liver, lung, and skeletal muscle tissues indicating translational read-through *in vivo* (Fig. 5*E*). Together, these results demonstrate the presence of endogenous MTCH2x. However, surprisingly, the MTCH2x antibody did not detect any band corresponding to the double-SCR product, MTCH2xx, in the conditions we tested.

### Double-SCR of MTCH2 makes the protein vulnerable for proteasome-mediated degradation

**Figure 6.**
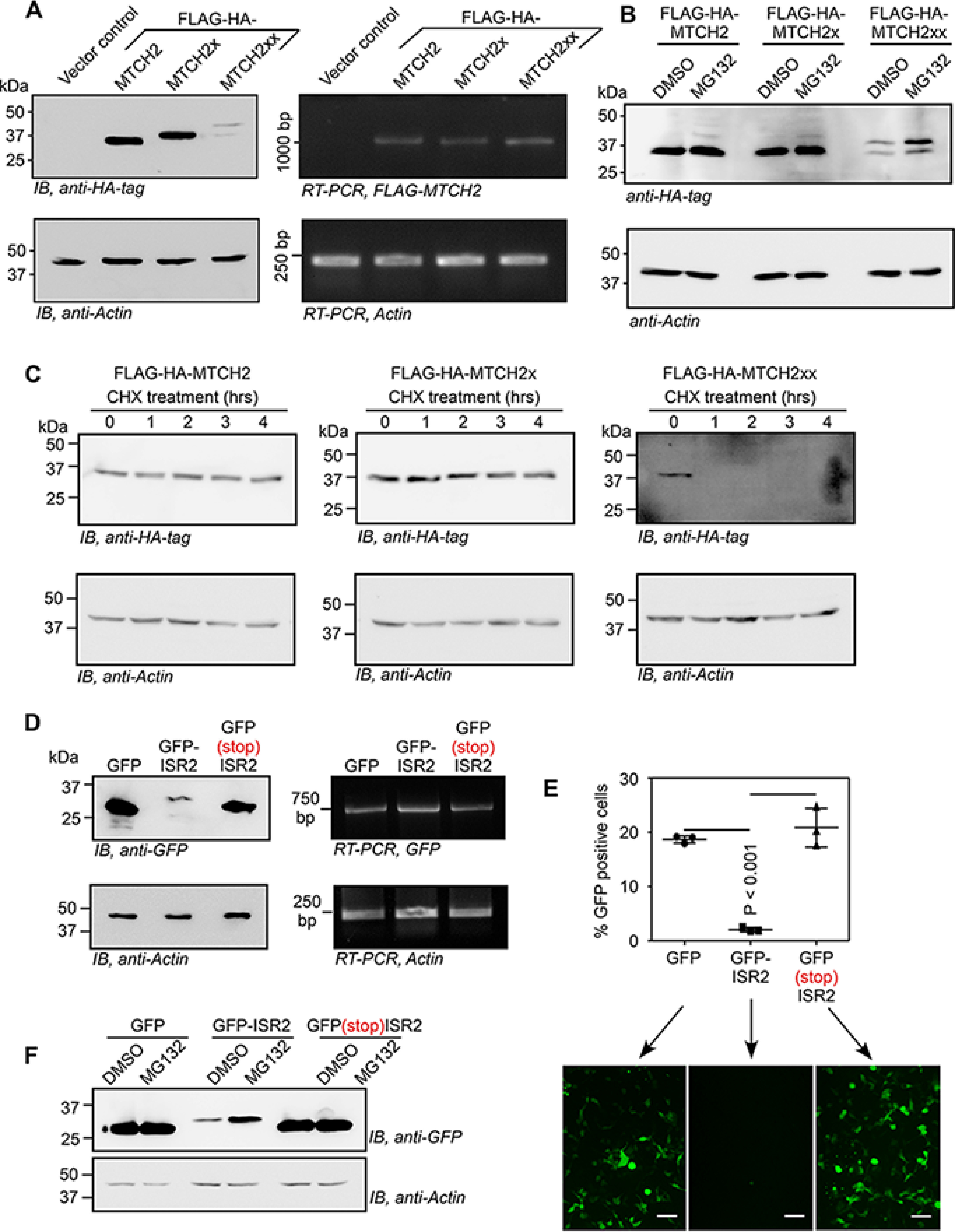
**Double-SCR of *MTCH2* reduces the stability of the protein generated.** *A* and *B,* Western blotting of lysates of HEK293 cells transfected with plasmids expressing FLAG-HA-tagged MTCH2 isoforms. RT-PCR results show comparable expression of all three constructs at the RNA level. In *B*, cells were treated with 10 μm MG132 for 5 h. Assays were done 24 h after transfection. *C, t*_1/2_ of FLAG-HA–tagged MTCH2 isoforms. HEK293 cells transfected with plasmids expressing MTCH2 isoforms were treated with 100 µg/ml of CHX for the indicated duration and their lysates were used for Western blot to check the expression of FLAG-HA–tagged isoforms. *D,* ISR2 sequence was cloned downstream of and in-frame with the coding sequence of GFP such that ISR2 is translated along with GFP. Western blotting and RT-PCR analyses of GFP expression with or without ISR2 or with a stop codon between them, in transfected HEK293 cells are shown. *E,* fluorescence microscopy images and flow cytometry analyses (mean ± S.D., *n* = 3) of HEK293 cells transfected with GFP-expressing constructs described in *D, scale bar*, 50 μm. Two-tailed Student's *t* test was used to calculate the *p* value. *F,* Western blotting image showing increased expression of GFP-ISR2 in cells treated with MG132.

To understand why endogenous MTCH2xx was undetectable by Western blotting, we expressed this isoform in HEK293 cells exogenously by cloning under the cytomegalovirus promoter with FLAG-HA tag; the first and second in-frame stop codons were mutated to sense codons (UGA to UCA and UAG to UCG) to maximize its expression. Remarkably, the FLAG-HA-MTCH2xx expression level was very low compared with that of FLAG-HA-MTCH2 and FLAG-HA-MTCH2x isoforms, when tested under identical conditions. RT-PCR revealed comparable expression of all three isoforms at the RNA level (Fig. 6*A*). The level of FLAG-HA-MTCH2xx increased when cells were treated with MG132, an inhibitor of proteasome, suggesting that this isoform is more susceptible for proteasome-mediated degradation (Fig. 6*B*). We then treated transfected cells with translation inhibitor cycloheximide (CHX) to estimate the *t*_1/2_ of FLAG-HA–tagged MTCH2 isoforms. Although MTCH2 and MTCH2x isoforms were stable until 4 h after CHX treatment, MTCH2xx was undetectable within 1 h after CHX treatment (Fig. 6*C*). In fact, MTCH2 and MTCH2x were detectable even after 36 h of CHX treatment (Fig. S6*A*). We then cloned ISR2 at the 3′ end of the coding sequence of GFP to investigate its effect on the expression of GFP. Translation of ISR2 drastically reduced the expression of GFP. However, the corresponding RNA levels were comparable. Insertion of an in-frame stop codon between GFP and ISR2 completely rescued the GFP expression demonstrating that the peptide sequence encoded in ISR2 is responsible for the reduced *t*_1/2_ (Fig. 6*D* and Fig. S6*B*). Similar observations were made by flow cytometry and fluorescence microscopy (Fig. 6*E*). Treatment of cells with MG132 increased the expression of GFP-ISR2 showing the involvement of proteasome-mediated degradation of ISR2-tagged GFP (Fig. 6*F*). Similarly, addition of ISR2-encoded peptide at the C terminus of mitochondrially targeted red fluorescent protein (mtDsRed) abolished its expression (Fig. S6*C*). Together, these results show that the amino acid sequence encoded by ISR2 makes the MTCH2xx isoform more susceptible to proteasome-mediated degradation compared with two other isoforms.

### Double-SCR of MTCH2 changes the localization of the isoform generated

**Figure 7.**
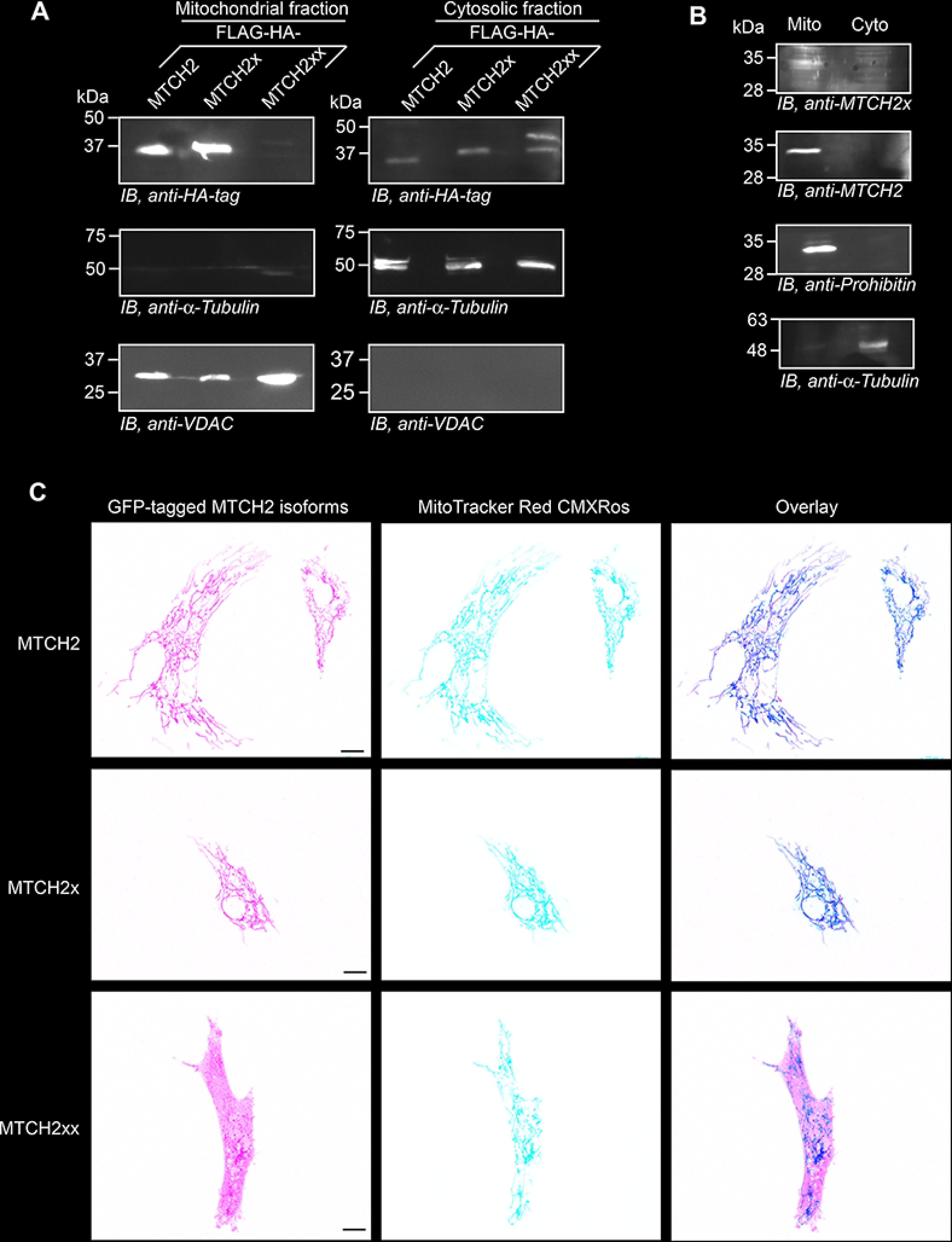
**Differential localization of MTCH2 isoforms.** *A,* plasmids expressing FLAG-HA–tagged isoforms of MTCH2 were transfected in HEK293 cells. Mitochondrial and cytoplasmic fractions from these cells were used for Western blotting. VDAC and α-tubulin were used as markers for mitochondria and cytoplasm, respectively. *B,* mitochondrial localization of endogenous MTCH2 and MTCH2x in HEK293 cells. Prohibitin was used as a marker for mitochondria. *C,* confocal fluorescence microscopy images showing cellular localization of GFP-tagged MTCH2 isoforms in primary bovine aortic endothelial cells. MitoTracker Red CMXRos was used to stain mitochondria. Percentage of GFP-positive cells in case of GFP-MTCH2xx was less compared with GFP-MTCH2 and GFP-MTCH2x. One such cell is shown. *Scale bar*, 10 μm.

MTCH2 is an outer mitochondrial membrane protein. Mislocalized mitochondrial proteins undergo proteasomal degradation (31–33). Hence, we investigated the localization of FLAG-HA–tagged MTCH2x and MTCH2xx. To maximize their expression, the canonical stop codon (for MTCH2x and MTCH2xx) and the first in-frame stop codon (for MTCH2xx) were mutated to sense codons as described above. Western blotting analysis of mitochondrial and cytosolic fractions revealed that, like MTCH2, MTCH2x was found predominantly in mitochondria (exogenous (Fig. 7*A*) and endogenous (Fig. 7*B*)). Although the expression level of MTCH2xx was much less, it was predominantly present in the cytoplasm (Fig. 7*A*). Similar observations were also made by fluorescence microscopy in primary bovine aortic endothelial cells and HEK293 cells (Fig. 7*C* and Fig. S^7^, *A* and *B*). Thus, double-SCR of *MTCH2* changes the localization as well as the stability of the isoform generated.

### Double-SCR of MTCH2 determines the cellular MTCH2 level

**Figure 8.**
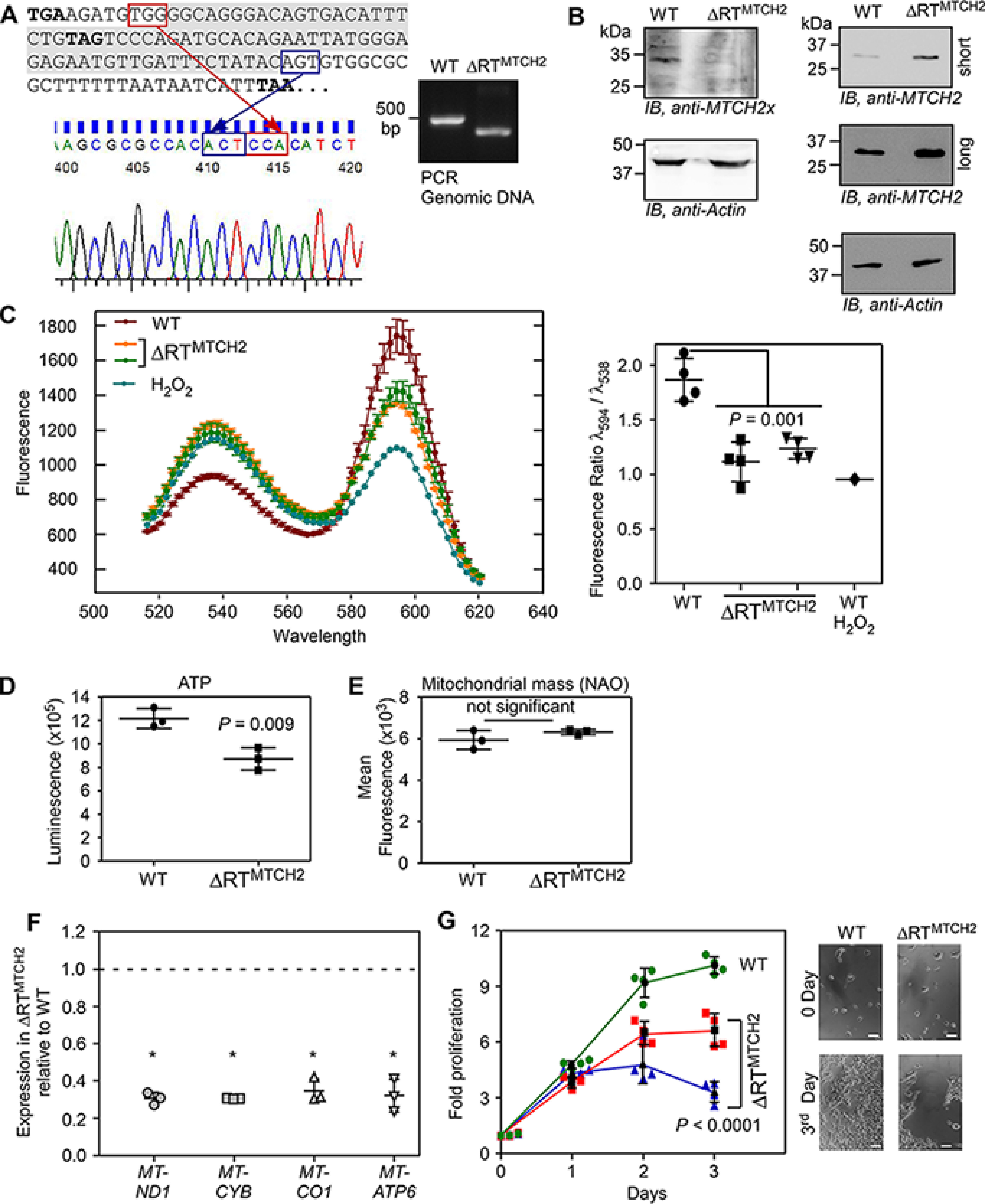
**Double-SCR of *MTCH2* is required to maintain normal mitochondrial membrane potential.** *A,* CRISPR-Cas9 technique was used to delete the most part of ISR1 and ISR2 in the genome of HEK293 cells (termed ΔRT^MTCH2^). Deletion was confirmed by PCR amplification from genomic DNA using primers flanking the targeted region, and by sequencing the product. Sequencing was performed using a reverse primer that generated reverse-complement sequence. A part of this is shown as an electropherogram. The deleted part in ISR1 and ISR2 is shown in *gray* background. Three in-frame stop codons are shown in *boldface*. *B,* Western blotting showing the absence of MTCH2x and increased levels of MTCH2 in ΔRT^MTCH2^ cells compared with the WT cells (*WT*). Long and short exposures are shown for MTCH2. *C,* JC-1 fluorescence profile shows reduced mitochondrial membrane potential in two different ΔRT^MTCH2^ clones (*left*). H_2_O_2_ treatment was used as a positive control for mitochondrial membrane depolarization. Ratio of fluorescence at λ_594_ to λ_538_ is shown (*right*) (*n* = 4). *D,* ΔRT^MTCH2^ cells show reduced ATP levels compared with the WT cells. Cells were incubated in serum-free DMEM containing 10 μm galactose for 48 h before measuring ATP levels using a luminescence-based assay (*n* = 3). *E,* WT and ΔRT^MTCH2^ cells have comparable mitochondrial mass. The flow cytometry was performed using the fluorescent probe, nonyl acridine orange (*NAO*) (*n* = 3). *F,* quantitative real-time PCR analyses of the expression of mitochondrial genes that encode components of the mitochondrial respiratory chain complexes in WT and ΔRT^MTCH2^ cells. Expression of mitochondrial genes relative to that of *ACTB* (β-actin) was calculated using the 2^−ΔΔ*Ct*^ method (*n* = 3). *, *p* < 0.01. *G,* proliferation profile of WT and two ΔRT^MTCH2^ clones. MTT assay was used to quantify proliferation (*n* = 4). Brightfield images of cells at 0 day and 3rd day are shown. *Scale bar*, 50 μm. All graphs show mean ± S.D. Results are representatives of at least three independent experiments done in triplicate. Two-tailed Student's *t* test was used to calculate the *p* value, except in *G*, where a two-way analysis of variance test was used.

The short *t*_1/2_ of MTCH2xx implies that double-SCR reduces the expression levels of MTCH2 by engaging the translation machinery in generating this unstable isoform. To investigate this aspect, we deleted 69 nucleotides from the ISR1 + ISR2 region in HEK293 cells using the CRISPR-Cas9 system. Because the deleted region includes 12^ISR2^, read-through across the stop codon will not occur in these cells (termed ΔRT^MTCH2^). Deletion was confirmed by PCR of that genomic region, and by sequencing the PCR product (Fig. 8*A*). Western blotting further confirmed the deletion as MTCH2x antibody failed to show any band in ΔRT^MTCH2^ cells. However, there was a 2-fold increase (2 ± 0.4, *n* = 3 experiments) in the expression of total MTCH2 protein in ΔRT^MTCH2^ cells compared with the WT cells (Fig. 8*B*). Because unstable MTCH2xx is not produced in ΔRT^MTCH2^ cells, *MTCH2* mRNAs generate only the canonical MTCH2 isoform increasing its level. Thus, double-SCR determines the cellular MTCH2 level.

### Double-SCR of MTCH2 is required to maintain normal mitochondrial membrane potential

Finally, we investigated the functional significance of double-SCR of *MTCH2.* Because MTCH2 is a regulator of oxidative phosphorylation, we analyzed the mitochondrial membrane potential (ΔΨm) in ΔRT^MTCH2^ cells using JC-1 dye. This fluorescent dye has two emission peaks depending on its oligomerization status, which in turn is determined by its cellular localization. Ratio of its fluorescence at these two peaks (λ_594_ to λ_538_) is an indicator of ΔΨm. This assay revealed significantly lower ΔΨm in ΔRT^MTCH2^ cells (Fig. 8*C*). This observation is consistent with previous reports (26, 34, 35), where reduced ΔΨm was observed when MTCH2 was overexpressed. Furthermore, the ATP level in ΔRT^MTCH2^ cells was also lower than that in WT cells (Fig. 8*D*). However, there was no change in the total mitochondrial mass in ΔRT^MTCH2^ cells compared with the WT cells (Fig. 8*E*). To understand the mechanism behind reduced ΔΨm and ATP in ΔRT^MTCH2^ cells, we investigated the expression of genes that encode mitochondrial respiratory chain complexes, which are responsible for the maintenance of normal ΔΨm. *MTCH2* knockout cells show increased expression of genes that encode mitochondrial subunits of the respiratory chain complexes (30, 36). We measured the expression of *MT-ND1* (complex I), *MT-CYB* (complex III), *MT-CO1* (complex IV), and *MT-ATP6* (complex V) by quantitative real-time PCR. Our analyses showed that expression of these genes was reduced in ΔRT^MTCH2^ cells compared with WT cells explaining reduced ΔΨm and ATP in ΔRT^MTCH2^ cells (Fig. 8*F*). Consistent with the reduced ΔΨm and low levels of cellular ATP concentration, ΔRT^MTCH2^ cells showed reduced proliferation compared with the WT cells (Fig. 8*G*). Together, these results demonstrate that programmed double-SCR of *MTCH2* contributes to the maintenance of normal ΔΨm by regulating the MTCH2 level.

## Discussion

**Figure 9.**
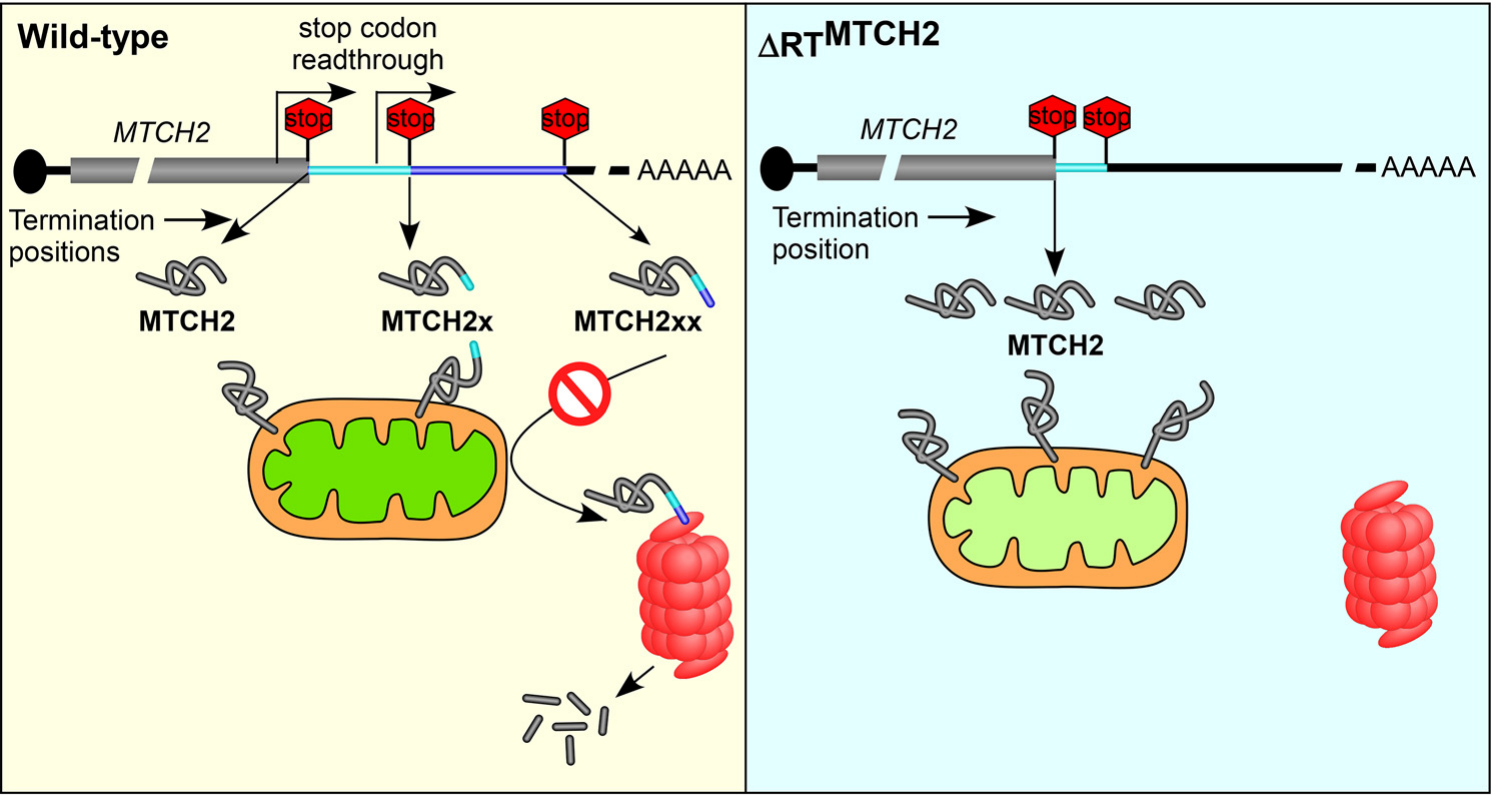
**Double-SCR of *MTCH2* mRNA regulates cellular level of MTCH2 protein and mitochondrial membrane potential.** Mammalian *MTCH2* mRNA has three evolutionarily conserved stop codons. This mRNA can generate three isoforms depending on the position of translation termination. Translation termination at the first stop codon results in the canonical isoform MTCH2. Read-through across the first stop codon and termination at the second stop codon results in an isoform with 11 extra amino acids at the C terminus (termed MTCH2x). Read-through across the first and the second stop codons (double-stop codon read-through), and termination at the third stop codon results in an isoform with 36 extra amino acids at the C terminus (termed MTCH2xx). MTCH2 and MTCH2x localize to mitochondria. However, MTCH2xx is predominantly cytoplasmic. This mislocalization and the unique C terminus makes this isoform susceptible for proteasomal degradation. Thus, double-stop codon read-through negatively regulates the level of MTCH2 available in the cell, which is critical for the maintenance of physiological level of mitochondrial membrane potential. In the absence of double-SCR, as seen in read-through deficient (ΔRT^MTCH2^) cells, MTCH2 level increases and the mitochondrial membrane potential decreases.

Organisms have evolved multiple mechanisms including alternative splicing, alternative initiation, RNA editing, post-translational modification, and SCR to expand their proteome. SCR has been reported in all domains of life. Although predicted (6, 8, 9), translational read-through across two stop codons (double-SCR) has not been demonstrated in any life form. Here, we demonstrate programmed double-SCR in mammalian *MTCH2* mRNA. This process generates an isoform that is predominantly cytoplasmic in its localization, unlike the canonical isoform. This mislocalization and the unique C terminus makes this long isoform vulnerable for proteasome-mediated degradation. Ultimately, this process reduces the total amount of MTCH2 generated from *MTCH2* mRNA. Thus, double-SCR of *MTCH2* regulates its own expression. Our results also show that this process is vital for the maintenance of normal ΔΨ_m_ (Fig. 9).

Physiological ΔΨm varies from −80 to −160 mV depending on the cell type and age (37). Higher ΔΨm will result in excessive production of harmful reactive oxygen species, whereas lower ΔΨm cannot generate sufficient quantity of ATP to meet the energy demand of the cell. Such variations are observed during physiological and pathological conditions. Several chronic diseases including atherosclerosis and type II diabetes are associated with increased cellular reactive oxygen species (38). Therefore, it is vital for eukaryotic cells to maintain physiological level of ΔΨm.

The ΔΨm is primarily regulated by the phosphorylation status of the complex proteins. It is also regulated allosterically by the cellular ATP level (37). Alterations in the cellular MTCH2 level can change the mitochondrial membrane potential (ΔΨm). For example, *MTCH2*-deficient hematopoietic stem cells show increased ΔΨm (36). Conversely, overexpression of MTCH2 results in reduced ΔΨm in HEK293 cells, DA3 cells, and mouse kidney (26, 34, 35). One way of changing the expression of MTCH2 is by altering the efficiency of double-SCR. An increase in the efficiency of this process will lead to a reduced MTCH2 level as the product is easily degraded. On the other hand, a decrease in the same will increase the MTCH2 level. *trans*-Acting factors such as micro-RNAs or RNA-binding proteins can serve as regulators by binding to the *cis*-acting RNA signal as reported previously (2, 3). Furthermore, abnormal changes in this read-through process can result in pathological consequences as ΔΨm determines the cellular reactive oxygen species level. For example, higher ΔΨm will result in excessive production of harmful reactive oxygen species (38).

Mitochondrial proteins are typically targeted to their destination (*i.e.* mitochondria) via a mitochondrial targeting signal, usually present at the N terminus. Interestingly, MTCH2 isoforms lack such a signal. One possibility is that MTCH2 reaches mitochondria by piggybacking another mitochondrial protein with a mitochondrial targeting signal. The unique C terminus of MTCH2xx may not allow such interaction explaining its predominantly cytoplasmic location.

Our experiments on the mechanism of the SCR revealed a 12-nucleotide sequence (12^ISR2^, UCCCAGAUGCAC) present at the 5′ end of ISR2 as the primary signal essential for both single- and double-SCR of *MTCH2*. Although this sequence was sufficient to induce SCR even in a heterologous context, the efficiency was not as high as observed in *MTCH2* context. This suggests that the sequence upstream of the canonical stop codon of *MTCH2* also plays a role in SCR. However, this upstream sequence alone was not sufficient to induce read-through as seen in our read-through assays. Interestingly, we observed a stretch of rare codons in the coding sequence of *MTCH2* very close to the canonical stop codon (Fig. S8). Rare codons can potentially slow down the translating ribosomes, and ribosomal pausing is associated with SCR (39, 40). Therefore, the rare codon stretch near the canonical stop codon of *MTCH2* might serve as the secondary signal that drives SCR, when the primary signal (12^ISR2^) is also present.

Regulation of gene expression by programmed SCR has been reported previously in two genes: mammalian *AMD1* and yeast *PDE2*. In case of *AMD1* (encodes adenosylmethionine decarboxylase 1), translational read-through across the canonical stop codon results in ribosomal stalling at the downstream stop codon limiting the synthesis of AMD1 protein (16). Translational read-through of *Saccharomyces cerevisiae PDE2* (encodes phosphodiesterase 2) creates an extension of 22 amino acids, which results in proteasome-dependent degradation instead of localization to the nucleus (41). Furthermore, nonprogrammed molecular errors or mutations that lead to SCR will also result in longer proteins that are degraded quickly (42, 43). Thus, SCR not only contributes to proteome expansion, it also regulates gene expression. The full potential of this process in cellular and organismal physiology remains to be uncovered.

## Experimental procedures

### Cell culture

HEK293 cells were cultured in Dulbecco's modified Eagle's medium (DMEM, HiMedia) supplemented with 10% fetal bovine serum (FBS, Gibco) and 1% antibiotics (10,000 units/ml of penicillin, 10,000 μg/ml of streptomycin, Lonza). Bovine aortic endothelial cells were cultured in DMEM/F-12 (1:1, Sigma) supplemented with 10% FBS and 1% antibiotics. Cells were maintained at 37 °C in a humidified atmosphere with 5% CO_2_.

### Construction of plasmids

The luciferase reporter constructs used for read-through assays were made in the pcDNA3.1B backbone as described (3). For single-SCR assay, a partial coding sequence of *MTCH2* (source: HEK293 cells) along with 45 nucleotides of the 3′ UTR (*i.e.* ISR1 + 12^ISR2^) were cloned (between HindIII and BamHI sites) upstream and in-frame with the coding sequence of firefly luciferase (*FLuc*) without its start codon. The second stop codon (UAG) was mutated to UAU such that read-through across the canonical stop codon will result in FLuc activity. For double-SCR assays, the coding sequence of *MTCH2* along with ISR1 and ISR2 were cloned (between HindIII and BamHI sites) upstream of and in-frame with *FLuc* without its start codon. A linker sequence (5′-GGCGGCUCCGGCGGCUCCCUCGUGCUCGAG-3′) was inserted between *MTCH2* and *FLuc*. Mutations were generated by PCR-based site-directed mutagenesis method. The GFP reporter constructs for GFP-based read-through assay were made by replacing *FLuc* with *GFP* in the constructs described above. pcDNA3.1B Myc/His vector was used for Western blotting-based translational read-through assay.

Plasmid constructs for dual-luciferase–based read-through assay were prepared in pcDNA3.1B vector. *Renilla* luciferase (RLuc) was cloned between HindIII and BamHI sites. FLuc with a linker and without AUG was cloned between XhoI and ApaI sites. Test sequences (ISR1 + 12^ISR2^ and ISR1 + ISR2) were cloned between BamHI and XhoI sites. A positive control was created by mutating the stop codon of RLuc (UAA) to UCA.

FLAG-HA-MTCH2, FLAG-HA-MTCH2x, and FLAG-HA-MTCH2xx constructs were made in pIRESneo-FLAG/HA plasmid (between NotI and EcoRI sites). For GFP-based localization studies, MTCH2, MTCH2x, and MTCH2xx were cloned in pEGFP-C1 vector between KpnI and BamHI sites. For constitutive overexpression of MTCH2x, the canonical stop codon UGA was mutated to UCA. For constitutive overexpression of MTCH2xx, the first (UGA) and second (UAG) stop codons were mutated to UCA and UCG, respectively. To test if the translation of ISR2 would reduce the expression levels of the tagged protein, ISR2 was cloned (between BamHI and NotI) downstream of and in-frame with GFP (cloned between HindIII and BamHI sites) in pcDNA3.1B vector. ISR2 was also cloned (between KpnI and BamHI) downstream of and in-frame with mtDsRed (cloned between HindIII and KpnI sites) in pcDNA3.1B. All constructs were confirmed by sequencing.

### Amino acid and nucleotide sequence alignment

The nucleotide sequences of the 3′ UTR of *MTCH2* mRNA from multiple mammals were obtained from NCBI. They were *in silico* translated using Expasy Translate tool to obtain the potential peptide sequences encoded by them. The amino acid sequences were aligned using Clustal Omega to analyze the conservation of the 3′ UTR of *MTCH2*.

### Antibodies

Polyclonal antibody specific to the C terminus extended region of MTCH2x was generated by injecting rabbits with the synthetic peptide RCGAGTVTFL. The same peptide was used for affinity purification of the antibody (Abgenex). Anti-MTCH2 (Sigma, SAB1101408 (Immunogen, amino acids 104-115 of human MTCH2) and Thermo Fisher Scientific, PA5-25406 (Immunogen, amino acids 75-103 of human MTCH2)), anti-HA (Sigma, 11867423001), anti-His (Sigma, SAB1305538), anti-GFP (BioLegend, 902602), anti-GAPDH (Sigma, G9295), anti-Actin (Sigma, A3854), anti-Prohibitin antibody (Thermo Fisher Scientific, MA5-12858), anti-VDAC antibody (BioLegend, 820701), anti-α-tubulin (Thermo Fisher Scientific, 62204), horseradish peroxidase-conjugated secondary antibodies (Thermo Fisher Scientific), and Alexa Fluor-conjugated secondary antibodies (Molecular Probes) were used at concentrations recommended by the manufacturer.

### Analysis of ribosomal profiling data

Ribosome profiling datasets were downloaded from NCBI's SRA using SRA Toolkit. Adapter sequences were first removed from the FASTQ files using the Fastp tool. Bowtie 2 was used for aligning the datasets to the mouse transcriptome. Reads having at least 24 nucleotides and a perfect match (no mismatches or gaps) with the reference sequence of the *MTCH2* (NM_001317242) were considered as *MTCH2*-specific ribosome-protected fragments. Datasets that displayed 50% or higher coverage of the inter stop codon regions (ISR1 and ISR2) with a decrease in ribosome density after the second in-frame stop codon (*i.e.* at least a 4-fold decrease in the ribosomal footprint density in the 3′ UTR compared with that in the ISRs) were considered positive for translational read-through of *MTCH2*.

### Analysis of MS data

MS raw files were downloaded from the ProteomeXchange Consortium using Aspera Connect. All the raw files were analyzed using MaxQuant version 1.6.2.6. Each raw data set was searched against a fasta file containing the whole mouse proteome (downloaded from the UniProt) along with 400 possible MTCH2xx protein sequences (all possible combinations of amino acids in place of the canonical stop codon and the first in-frame stop codon). The number of missed cleavages was set to 5 with the false discovery rate for peptide spectrum matches set to 0.05. Protein false discovery rate filter was disabled. Default values were used for all other parameters. In-house Python scripts were used to identify peptides aligning to the canonical coding sequence and ISRs of *MTCH2*. Using the “tblastn” tool, we made sure that the identified peptides were unique to the coding sequence and ISRs of *MTCH2*.

### Translational read-through assays

#### 

##### Luminescence-based assays

HEK293 cells were seeded in 24-well–plates at 75–95% confluence. They were transfected with 500 ng/well of luciferase-encoding plasmids using Lipofectamine 2000 (Invitrogen). In single luciferase assays, 25 ng/well of RLuc was co-transfected as a transfection control. Twenty-four hours after transfection, FLuc and RLuc activities were measured using Dual-Luciferase Reporter Assay System (Promega) in Glomax Explorer (Promega). For *in vitro* assay, the constructs were linearized using NotI or ApaI enzyme. The linearized plasmid was *in vitro* transcribed using T7 RNA polymerase (Thermo Fisher Scientific) at 37 °C for 2 h. Two μg of *in vitro* transcribed RNA was *in vitro* translated using rabbit reticulocyte lysate system (Promega) at 30 °C for 2 h. Luciferase activity was measured as described above.

##### Fluorescence-based assay

500 ng/well of GFP-encoding plasmids were transfected in HEK293 cells seeded in a 24-well–plate using Lipofectamine 2000. Fluorescence was analyzed both by fluorescence microscopy (Olympus IX73) and flow cytometry (CytoFLEX S, Beckman Coulter).

##### Western blotting-based assay

Lysates of cells transfected with Myc/His constructs were subjected to Ni-NTA enrichment by incubating with Ni-NTA-agarose beads overnight (Thermo Fisher Scientific). His-tagged proteins were eluted (elution buffer: 300 mm NaCl and 100 mm imidazole in 20 mm phosphate buffer), and the elutions were subjected to Western blotting analysis to detect the read-through protein using anti-His antibody.

### RNA isolation and RT-PCR analysis

Total RNA was isolated using TRI reagent (Sigma). cDNA synthesis was carried out using the oligo(dT) universal reverse primer with RevertAid RT enzyme (Thermo Fisher Scientific). Gene-specific primers were used for semi-quantitative analysis of the mRNA levels. Primer sequences are (5′ to 3′): *FLUC*: CAACTGCATAAGGCTATGAAGAGA; ATTTGTATTCAGCCCATATCGTTT; *GAPDH*: CCACCCATGGCAAATTCCATGGCA; TCTAGACGGCAGGTCAGGTCCACC; *ACTB* (β-Actin): AGAGCTACGAGCTGCCTGAC; AGCACTGTGTTGGCGTACAG; *GFP*: ATGGTGAGCAAGGGCGAGGAGCTGTT; CTTGTACAGCTCGTCCATGCCGAG; and *FLAG-MTCH2*: GATTACAAGGATGACGACGATAA; AATTAACATTTTCAGGTCACAA.

### Western blotting analysis

Cells were lysed in lysis buffer (20 mm Tris-HCl, 150 mm NaCl, 1 mm EDTA, 1% Triton X-100 with protease inhibitor mixture (Roche Applied Science)). The protein concentration was determined using the Protein Assay Dye Reagent (Bio-Rad). 20-100 μg of the cell lysate or 20-100 μg of mitochondrial lysate or 10-50 μg of tissue lysate was subjected to denaturing SDS-PAGE in a 12.5% Tris glycine gel. The proteins were then transferred onto a polyvinylidene difluoride membrane (Merck) and blocked with a blocking agent (5% milk or 3% BSA in PBS). The membrane was probed with the specific primary antibody and then with horseradish peroxidase-conjugated secondary antibody. The blot was developed using Clarity ECL reagent (Bio-Rad) and the images were recorded using LAS-3000 or LAS-4000 imager (Fujifilm). Quantification of band intensities was carried out using ImageJ software.

### shRNA

shRNA constructs (MISSION shRNA Sigma) were transfected in HEK293 cells using Lipofectamine 2000. Cells were then selected for 1 week using puromycin and subjected to immunoblot analysis. The shRNA specific to human *MTCH2* mRNA target the following sequences: shRNA 1: 5′-CCTCCAACAATAGGACGAAAT-3′ (TRCN0000059393); shRNA 2: 5′-GCCTTCTAGGTGACATCCTTTCTTT-3′ (TRCN0000059394); shRNA 3: 5′-CCCTTTGTGCTTGTCTCCAATCT-3′ (TRCN0000059395).

### Isolation of mitochondria

Mitochondria were isolated based by a previously described method (44). HEK293 cells were harvested and centrifuged at 600 × *g* for 10 min. The pellet was resuspended in mitochondrial isolation buffer (0.01 m Tris-MOPS, 0.001 m EGTA/Tris, and 0.5 m sucrose) and homogenized at 3000 rpm using a motor-driven tissue grinder (Genetix). The homogenate was centrifuged at 600 × *g* for 10 min. The supernatant was again centrifuged at 7000 × *g* for 10 min. The mitochondrial pellet was suspended in mitochondrial isolation buffer. The supernatant obtained was the cytosolic fraction.

### Microscopy

Transfected cells were reseeded in 350-mm dishes with a glass bottom and taken for imaging. Images were obtained using 1.0 Airy pinhole in TCS SP8 confocal microscope (Leica) or LSM 880 Confocal Laser Scanning Microscope (Zeiss) or FLUOVIEW FV3000 confocal microscope (Olympus). 100 nm Mitotracker Red CMXRos (Life Technologies) was used to stain mitochondria. Brightness and contrast were increased equally in all the images shown in Fig. 3*B* and Fig. S7.

### Protein t_1/2_ measurement

N-terminal FLAG-HA–tagged MTCH2, MTCH2x, and MTCH2xx constructs were transfected in HEK293 cells using Lipofectamine 2000. 20 h post-transfection, cells were treated with 100 μg/ml of cycloheximide along with vehicle control. Cells were harvested at different time points and subjected to Western blotting analysis.

### Deletion of MTCH2 translational read-through region in/award1/ctgiq HEK293 cells (ΔRT^MTCH2^) using CRISPR/cas9 system

The sgRNAs (5′-AAAATGTTAATTTGAAGATG-3′ and 5′-ATGTTGATTTCTATACAGTG-3′) were cloned in pSpCas9(BB)-2A-GFP (PX458) plasmid. Two μg of each sgRNA-expressing plasmids were transfected in HEK293 cells at 75% confluence in a 35-mm dish using Lipofectamine 2000. 24 h post-transfection, GFP-positive cells were sorted and seeded (single cell per well) in a 96-well–plate using FACSAria™ II sorter (BD Biosciences). They were further expanded and screened for the deletion by PCR using primers flanking the region of expected deletion (5′-AAGGTCCCCTTTGGGAAG-3′ and 5′-AGGGTCTACCCAAGAAAA-3′). The expected PCR products were WT, 488 base pairs, and ΔRT^MTCH2^, 419 base pairs. Clones showing successful deletion were further confirmed by sequencing of PCR product and Western blotting analysis.

### Measurement of mitochondrial membrane potential

Cells were seeded in a 96-well–plate (10^4^ cells/well). 5 μm JC-1 dye (Sigma) was added to each well and the plate was incubated for 15 min. Fluorescence intensity scan was then carried out with excitation at 495 nm and emission spectrum recorded from 510 to 620 nm (The Infinite M Nano+ microplate reader, Tecan). The ratio of fluorescence intensity at 594 nm to that at 538 nm was calculated and used to indicate the mitochondrial membrane potential. H_2_O_2_ treatment (20 μm) was used as a positive control to induce mitochondrial membrane depolarization.

### ATP measurement

This was carried out using the ATP detection reagent of Mitochondrial ToxGlo Assay kit (Promega). Cells were seeded (10^4^ cells/well) in a 96-well–plate. They were incubated in serum-free DMEM containing 10 mm galactose for 48 h, and then lysed using ATP lysis buffer for 10 min at room temperature. Luminescence was recorded using Glomax Explorer (Promega).

### Mitochondrial mass measurement

HEK293 cells (10^5^ cells/well in a 24-well–plate) were incubated with 100 nm NAO (Molecular Probes) for 20 min. Stained cells were washed, trypsinized, and harvested. The cell pellet was resuspended in 300 μl of PBS and subjected to flow cytometry analysis (CytoFLEX S, Beckman Coulter).

### Quantitative real-time PCR

Total RNA was extracted using RNAiso Plus (TaKaRa) following the manufacturer's instructions from WT and ΔRT^MTCH2^ cells. BioPhotometer (Eppendorf) was used to measure the concentration and the purity of RNA. For cDNA synthesis, equal concentration of RNA of the experimental samples (1 to 3 μg of RNA) was used to synthesize cDNA using oligo(dT) primers and RevertAid Reverse Transcriptase as per the manufacturer's instructions. Quantitative real-time PCR was carried out by setting up 10-μl reactions containing 1.5 μl of cDNA, 330 nM gene-specific primers and 5 μl of 2× TB Green Premix Ex TaqII (Takara) in iCycler iQ PCR Plates 96-well (Bio-Rad) using iQ5 real-time PCR system (Bio-Rad). All quantitative PCR were carried out in triplicates. The cycling conditions used were as follow: 95 °C for 5 min followed by 40 cycles of 95 °C for 30 s, 55 °C for 30 s, 72 °C for 30 s, and a single final step at 72 °C for 5 min. Melting curves were generated after each reaction. The specificity of the primers was verified by visualizing the amplified product using agarose gel electrophoresis. Expression of mitochondrial genes relative to that of β-Actin was calculated using the 2^-ΔΔ^*^Ct^* method. Sequences of the primers (5′ to 3′) are as follows: *ACTB* (β-Actin): AGAGCTACGAGCTGCCTGAC; AGCACTGTGTTGGCGTACAG; *MT-ATP6*: TAGCCATACACAACACTAAAGGACGA; GGGCATTTTTAATCTTAGAGCGAAA; *MT-CO1*: GACGTAGACACACGAGCATATTTCA; AGGACATAGTGGAAGTGAGCTACAAC; *MT-CYB*: ATCACTCGAGACGTAAATTATGGCT; TGAACTAGGTCTGTCCCAATGTATG; *MT-ND1*: CCACCTCTAGCCTAGCCGTTTA; GGGTCATGATGGCAGGAGTAAT.

### Cell proliferation assay

WT and ΔRT^MTCH2^ cells were seeded (5 × 10^3^ cells/well) in a 96-well–plate. MTT (Sigma, 50 μg/well) was added at different time points and incubated for 2 h. The medium was removed, 100 μl of DMSO was added and incubated further for 15 min. The supernatant was transferred to another plate and the absorbance at 570 nm was measured using a microplate reader (VERSAmax, Molecular devices).

### Statistics

To test if the differences observed between samples in experiments were significant, two-sided Student's *t* test was used when samples showed normal distribution. If equal variance was not observed, Welch's correction was applied. Two-way analysis of variance test was used to test if the difference observed in cell proliferation assay was significant.

## Data availability

All data are contained within the manuscript.

## Supplementary Material

Supporting Information
