# Supplementary material for: Stop codon read-through of mammalian MTCH2 leading to an unstable isoform regulates mitochondrial membrane potential
Source: J Biol Chem. 2020 Oct 7;295(50):17009–26. doi: 10.1074/jbc.RA120.014253 (PMC7863902; doi:10.1074/jbc.RA120.014253)
Supplement: Supporting Information [file supp_295_50_17009__index.html]

Stop codon readthrough of mammalian MTCH2 leading to an unstable isoform regulates mitochondrial membrane potential — Double-stop codon readthrough of MTCH2 — Stop codon read-through of mammalian MTCH2 leading to an unstable isoform regulates mitochondrial membrane potential — Double-stop codon read-through of MTCH2 — Supporting Information 

# Stop codon read-through of mammalian *MTCH2* leading to an unstable isoform regulates mitochondrial membrane potential

## Supporting Information

- Supporting Information (to be published online) - Figures S1 to S8 and Table S1
- Original Source Data - Source/Raw data of readthrough assays, Western blots and images.
