# Supplementary material for: Stop codon read-through of mammalian MTCH2 leading to an unstable isoform regulates mitochondrial membrane potential
Source: J Biol Chem. 2020 Oct 7;295(50):17009–26. doi: 10.1074/jbc.RA120.014253 (PMC7863902; doi:10.1074/jbc.RA120.014253)
Supplement: Supporting Information [file supp_RA120.014253_160699_2_supp_606517_qhl9h5.pdf]

Figure S1

H.sapiens	CUGAAAAUGUUAAUU**UGA**AGAUGUGGGGCAGGGACAGUGACAUUUCUG**UAG**UCCCAGAUGCACAGA...
C.syrichta	CUGAGAAUGUUAAUU**UGA**AGAUGUGGGGCAGGGACAGUGACAUUUCUA**UAG**UCCCAGAUGCACAGA...
O.orca	CUGAGAAUGUUAAUU**UGA**AGAUGGGGGGCAGGGACAGUGACAUUUCUA**UAG**UCCCAGAUGCACAGA...
S.scrofa	CUGAGAAUGUUAAUU**UGA**AGAUGUGGGGCAGGGACAGUGACAUUUCUA**UAG**UCCCAGAUGCACAGA...
E.asinus	CUGAGAAUGUUAAUU**UGA**AGAUGUGGGGCAGGGACAGUGACAUUUCUA**UAG**UCCCAGAUGCACAGA...
C.cristata	CUGAGAAUGUUAAUU**UGA**AGAUGUGGGACAGGGACAGUGACAUUUCUA**UAG**UCCCAGAUGCACAGA...
M.natalensis	CUGAGAAUGUUAAUU**UGA**AGAUGUGGGACAGGGACAGUGACAUUUCUA**UAG**UCCCAGAUGCACAGA...
M.davidii	CUGAGAAUGUUAAUU**UGA**AGAUGUGGGACAGGGACAGUGACAUUUCUA**UAG**UCCCAGAUGCACAGA...
I.tridecemlineatus	CUGAGAAUGUUAAUU**UGA**AGAUGUGGGGCAGGGACAGUGACAUUUCUA**UAG**UCCCAGACGCACAGA...
M.marmota	CUGAGAAUGUUAAUU**UGA**AGAUGUGGGGCAGGGACAGUGACAUUUCUA**UAG**UCCCAGACGCACAGA...
L.africana	CUGAGAAUGUUAAUU**UGA**AGAUGUGGGGCAGGGACAGUGACAUUUCUG**UAG**UCCCAGAUGCACAGA...
M.domestica	CUGAGGAUGUUAAUU**UGA**AGAUGUGGGGCAGGGGCAAUGACAUUUCUG**UAG**UCCCAGAUGCACAGA...
M.musculus	CUAAGAAUGUUAAUC**UGA**AGAUGUGGGGCAGGGACAGUGACAUUUCUA**UAG**UCCC-AAUGCACAGA...

H.sapiens	...AUUAUGGGAGAGAAUGUUGAUUUCUAUACAGUGUGGCGCGCUUUUU--UAAUAAUCAUU**UAA**...
C.syrichta	...AUUAUGGGAGAGAAUAUUGAUUUCUAUACAGUACGGCGUGCUUUUU--UAAUAAUCAUU**UAA**...
O.orca	...AUUAUGGGAGAGAAUGUUGAUUUCUAUACAGUGUGGCACGCUUUUU--UAAUAAUCAUU**UAA**...
S.scrofa	...AUUAUGGGAGAGAAUGUUGAUUUCUAUACAGUGUGGUGCGCUUUUU--UAAUAAUCAUU**UAA**...
E.asinus	...AUUAUGGGAGAGAAUGUUGAUUUCUAUACAGUGUGGUGCGCUUUUU--UAAUAAUCACU**UAA**...
C.cristata	...AUUAUGGGAGAGAAUGUUGAUUUAUAUACAGUGUGGCGCGCUUUUU--UAAUAAUCAUU**UAA**...
M.natalensis	...AUUAUGGGAGAGAAUGUUGAUUACUAUACAGUAUGGCACGCUUUUU--UAAUAAUCAUU**UGA**...
M.davidii	...AUUAUGGGAGAGAAUGUUGAUUUCUAUACAGUGUGGCACGCUUUUU--UAAUAAUCAUU**UAA**...
I.tridecemlineatus	...AUUAUGGGAGAGAAUGUUGAUUUCUAUACAGUGUGGCGCGCUUUUU--UAAUAAUCAUU**UAA**...
M.marmota	...AUUAUGGGAGAGAAUGUUGAUUUCUAUACAGUGUGGCGCGCUUUUU--UAAUAAUCAUU**UAA**...
L.Africana	...AUUAUGGGAGAGAAUGUUGAUUUCUAUACAGUGUGGCGCGCUUUUU--UAAUAAUCAUU**UAA**...
M.domestica	...AUUAUGGGAGAGAAUGUUGAUUUCUAUACAGUGUGGUGCGCUUUUUUU**UAAUAA**UCAUUUAA...
M.musculus	...AUUAUGGGAGAGAAUGUUGAUUUCUAUACAGUGUGGCACGCUUUUU--**UAAUAA**UCAUUUAA...

Figure S1. Proximal 3'UTR of *MTCH2* is highly conserved among mammals.
Alignment of nucleotide sequences of the distal part (15 nts) of the coding sequence and the proximal 3'UTR of *MTCH2* from multiple mammalian species. Canonical stop codon (UGA), first (UAG) and second (UAA) in-frame stop codons are shown in bold. Conserved residues are shown in grey background.

Figure S2

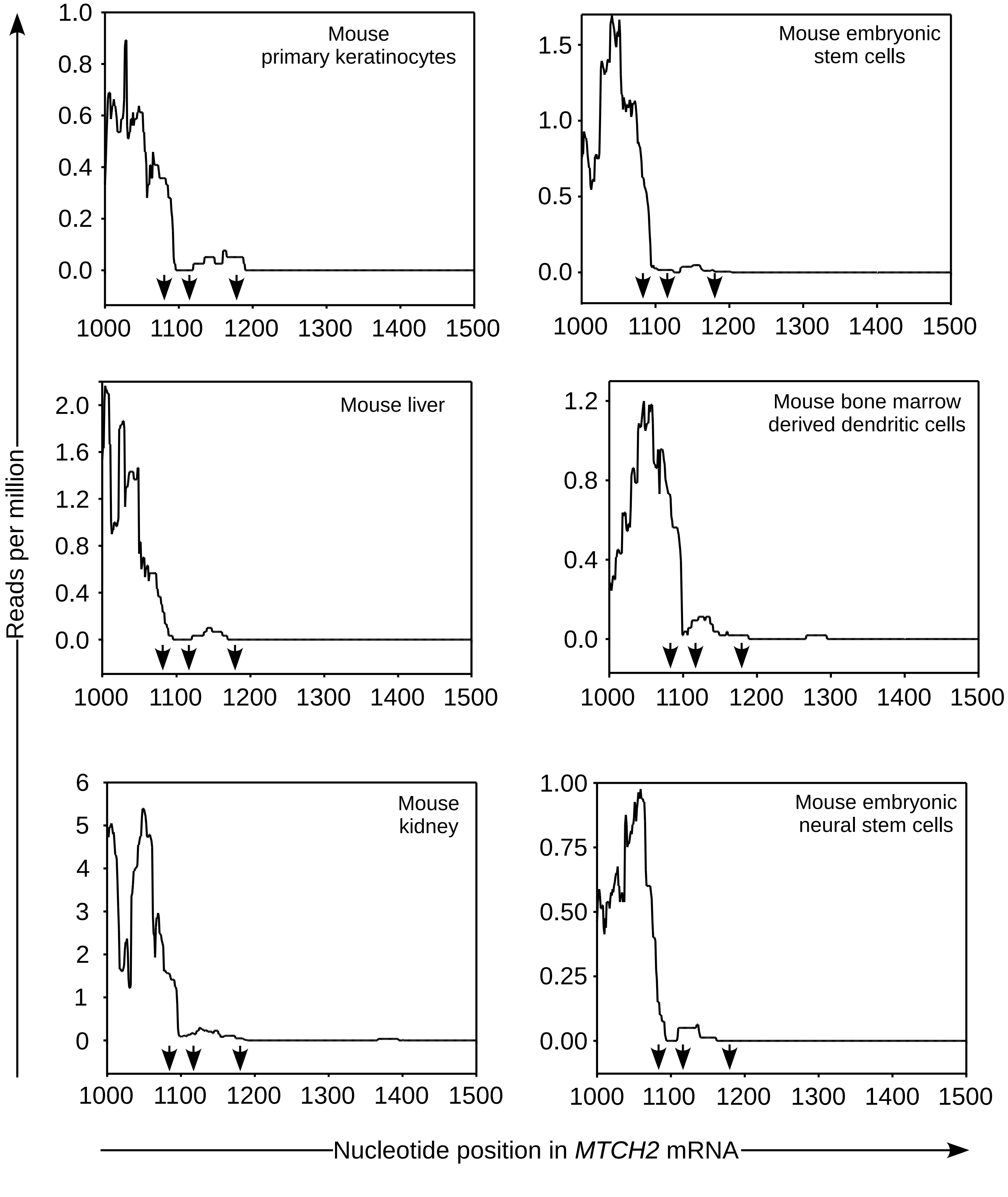

Figure S2. Ribosome profiling data from multiple sources supports double-SCR of *MTCH2*.
MTCH2 ribosome profile in mouse primary keratinocytes (SRR3665720), mouse embryonic stem cells (SRR606204), mouse liver (SRR3623564), mouse bone marrow derived dendritic cells (SRR2724718), mouse kidney (SRR3623564) and mouse embryonic neural stem cells (SRR1382096) showing the presence of ribosomes in ISR1 and ISR2. Arrows indicate the position of three in-frame stop codons (UGA, UAG, UAA).

Figure S3

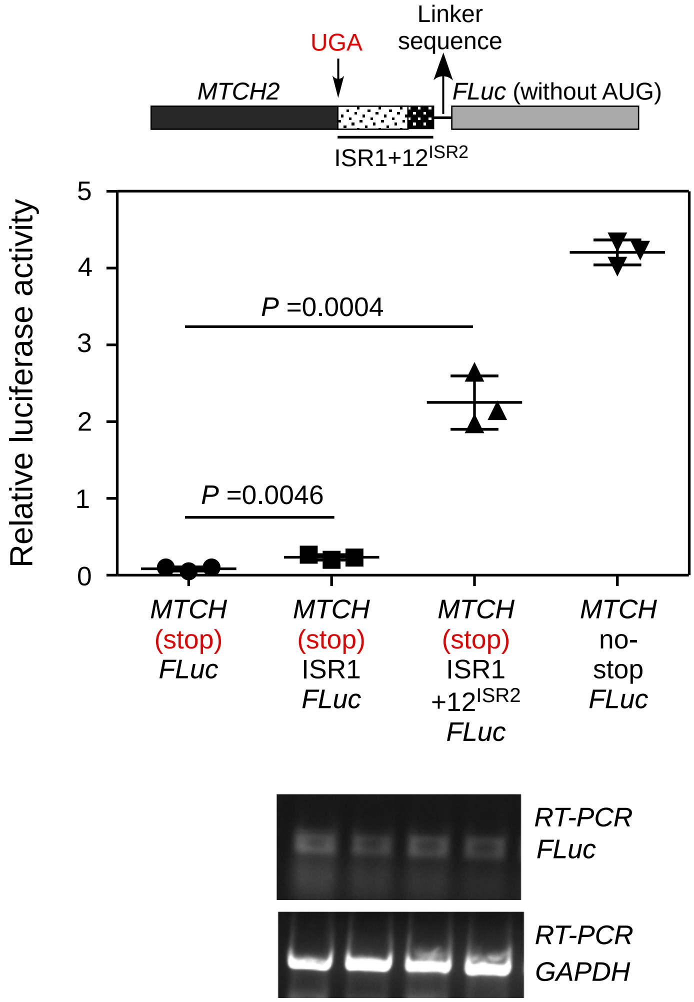

Fig. S3. Demonstration of translational readthrough across the canonical stop codon of *MTCH2*. Luminescence-based assay: Plasmid expressing in-frame MTCH2-(UGA)-(ISR1+12^{ISR2})-FLuc and its variants were transfected in HEK293 cells. FLuc activity relative to the activity of co-transfected renilla luciferase (RLuc) is shown. A construct without ISR1+12^{ISR2} between *MTCH2* and *FLuc* served as negative control. RT-PCR of *FLuc* mRNA is shown. Graph shows Mean ± SD (n = 3). Results are representatives of three independent experiments done in triplicate. Two-tailed Student's t-test was used to calculate the *P* value. FLuc, firefly luciferase; MTCH, MTCH2; 12^{ISR2}, First 12 nucleotides of ISR2 (UCCCAGAUGCAC).

Figure S4

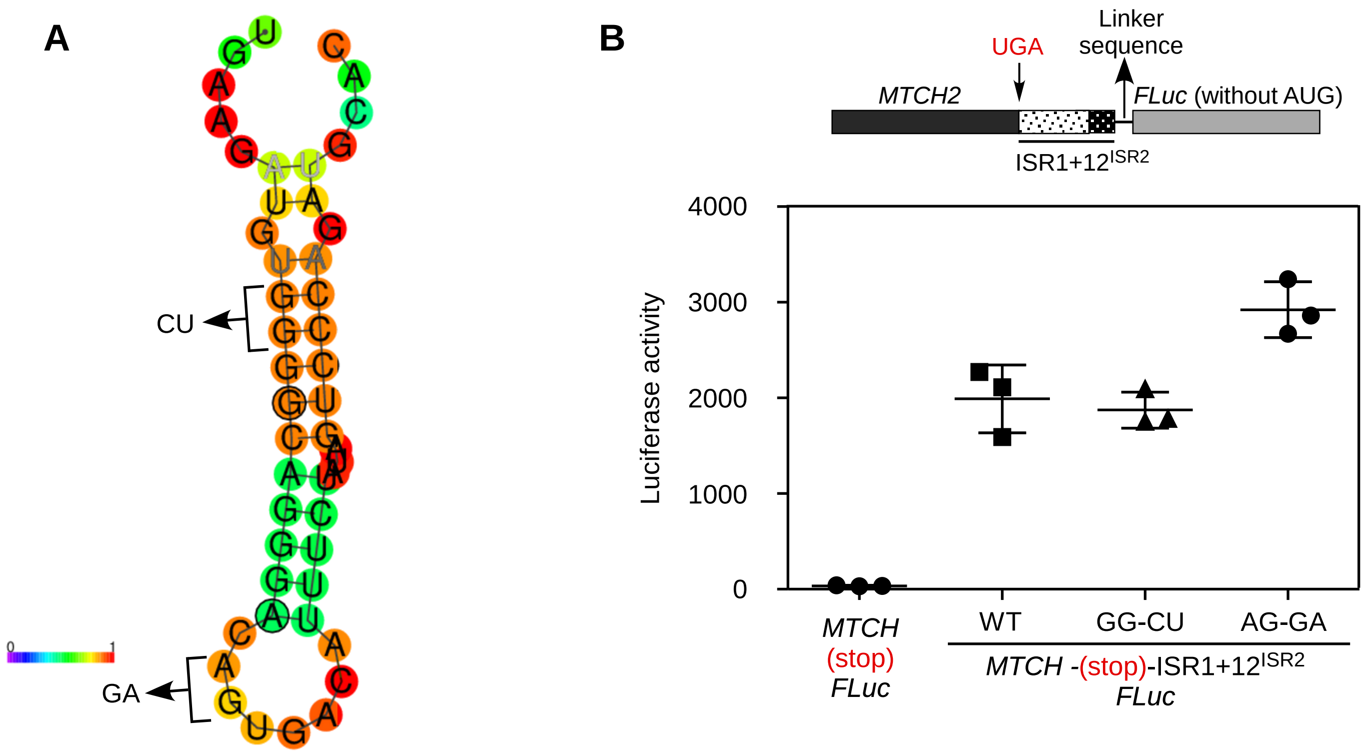

Figure S4. Predicted secondary structure of ISR1+12^{ISR2}

(A) Stem-loop structure of ISR1+12^{ISR2} as predicted by RNAalifold webserver. Colour code indicates probability of base-pairing. Mutations performed for readthrough assay are indicated by arrow.

(B) Luminescence-based assay: Plasmid expressing in-frame *MTCH2*-(UGA)-(ISR1+12^{ISR2})-FLuc and its mutants were subjected to *in vitro* transcription and *in vitro* translation using rabbit reticulocyte lysate. Luciferase activity is shown (Mean ± SD, n = 3). Results are representatives of three independent experiments done in triplicate. FLuc, firefly luciferase; MTCH, MTCH2.

Figure S5

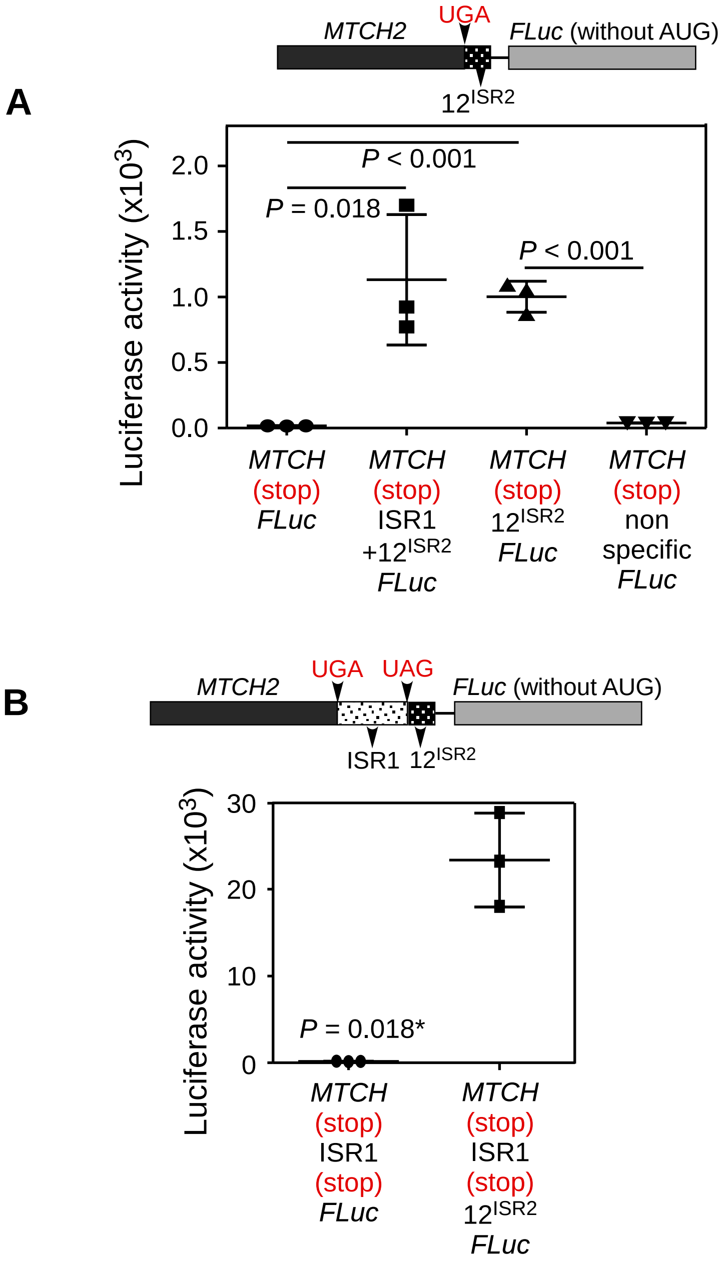

Figure S5. 12^{ISR2} is sufficient to drive SCR in *MTCH2*

(A) 12^{ISR2} alone can drive readthrough across the canonical stop codon of *MTCH2*. Construct having just 12^{ISR2} between *MTCH2* and *FLuc* was used (see schematic). Results of luciferase-based readthrough assay carried out *in vitro* using rabbit reticulocyte lysate system are shown.

(B) 12^{ISR2} alone can drive double-SCR of *MTCH2*. Construct having ISR1+12^{ISR2} along with the two stop codons between *MTCH2* and *FLuc* was used (see schematic). Results of luciferase-based readthrough assay carried out *in vitro* using rabbit reticulocyte lysate system are shown.

Bars in both graphs indicate Mean ± SD (n = 3). Results are representatives of at least three independent experiments done in triplicate. Two-tailed Student's t-test was used to calculate the *P* value. FLuc, firefly luciferase; MTCH, MTCH2; 12^{ISR2}, First 12 nucleotides of ISR2 (UCCCAGAUGCAC)

Figure S6

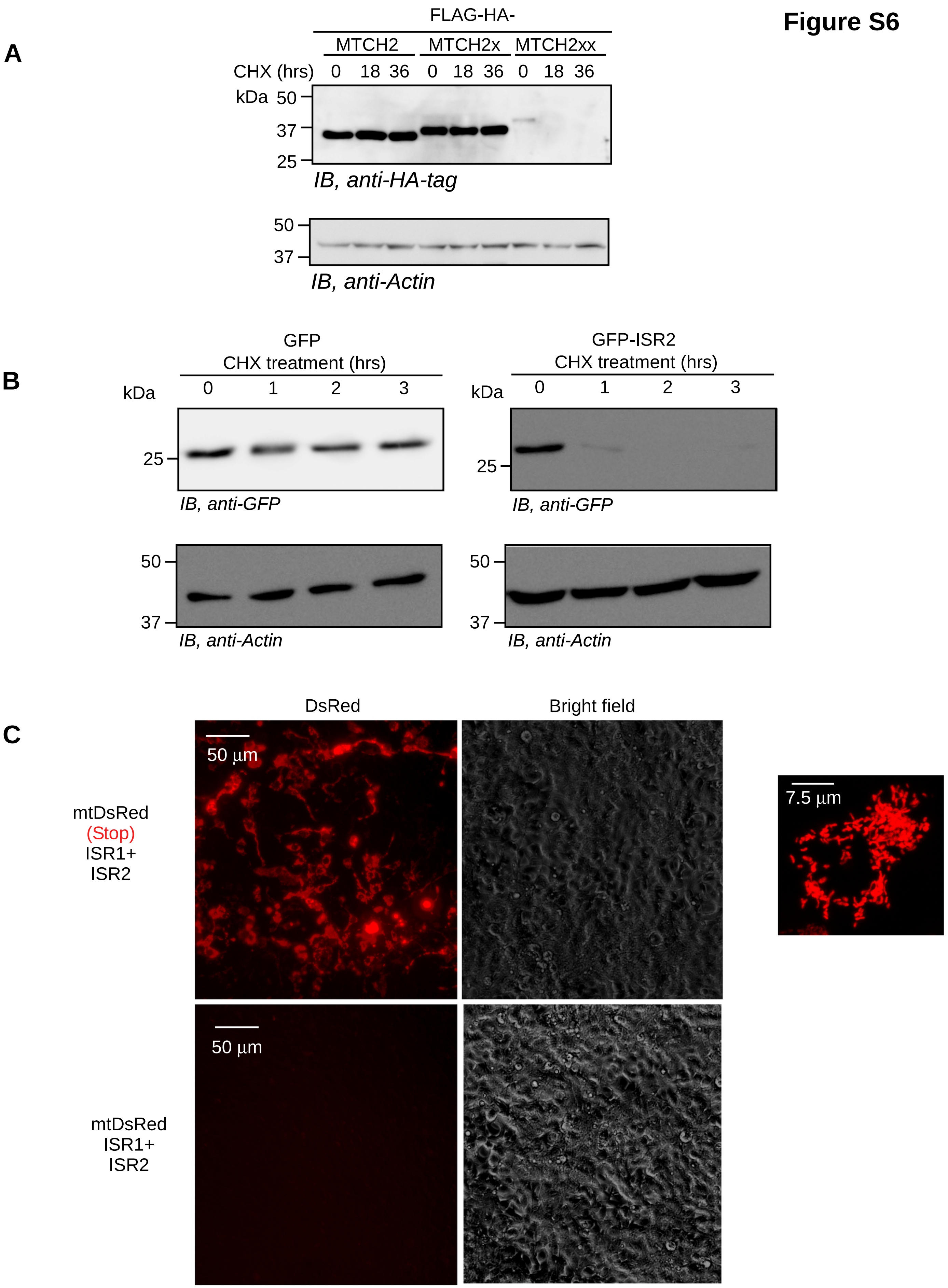

A
FLAG-HA-
MTCH2
MTCH2x
MTCH2xx
CHX (hrs) 0 18 36 0 18 36 0 18 36
kDa 50
37
25
IB, anti-HA-tag
50
37
IB, anti-Actin
B
GFP
CHX treatment (hrs)
kDa
0 1 2 3
25
IB, anti-GFP
GFP-ISR2
CHX treatment (hrs)
kDa
0 1 2 3
25
IB, anti-GFP
50
37
IB, anti-Actin
50
37
IB, anti-Actin
C
DsRed
Bright field
50 µm
mtDsRed
(Stop)
ISR1+
ISR2
7.5 µm
50 µm
mtDsRed
ISR1+
ISR2

Figure S6. Translation of ISR2 reduces the stability of the protein generated

(A) HEK293 cells transfected with plasmids expressing FLAG-HA-tagged MTCH2 isoforms were treated with cycloheximide (100 µg/ml) for the indicated durations and the lysates were subjected to immunoblot analysis to determine the relative stability.

(B) HEK293 cells transfected with plasmids expressing GFP and GFP-ISR2 were treated with cycloheximide (100 µg/ml) for the indicated durations and the lysates were subjected to immunoblot analysis to determine the effect of ISR2 on protein stability.

(C) Fluorescence microscopy images of HEK293 cells transfected with mtDsRed-ISR1+ISR2 are shown. A representative high magnification image with mitochondrial localization of mtDsRed is also shown (right).

Figure S7

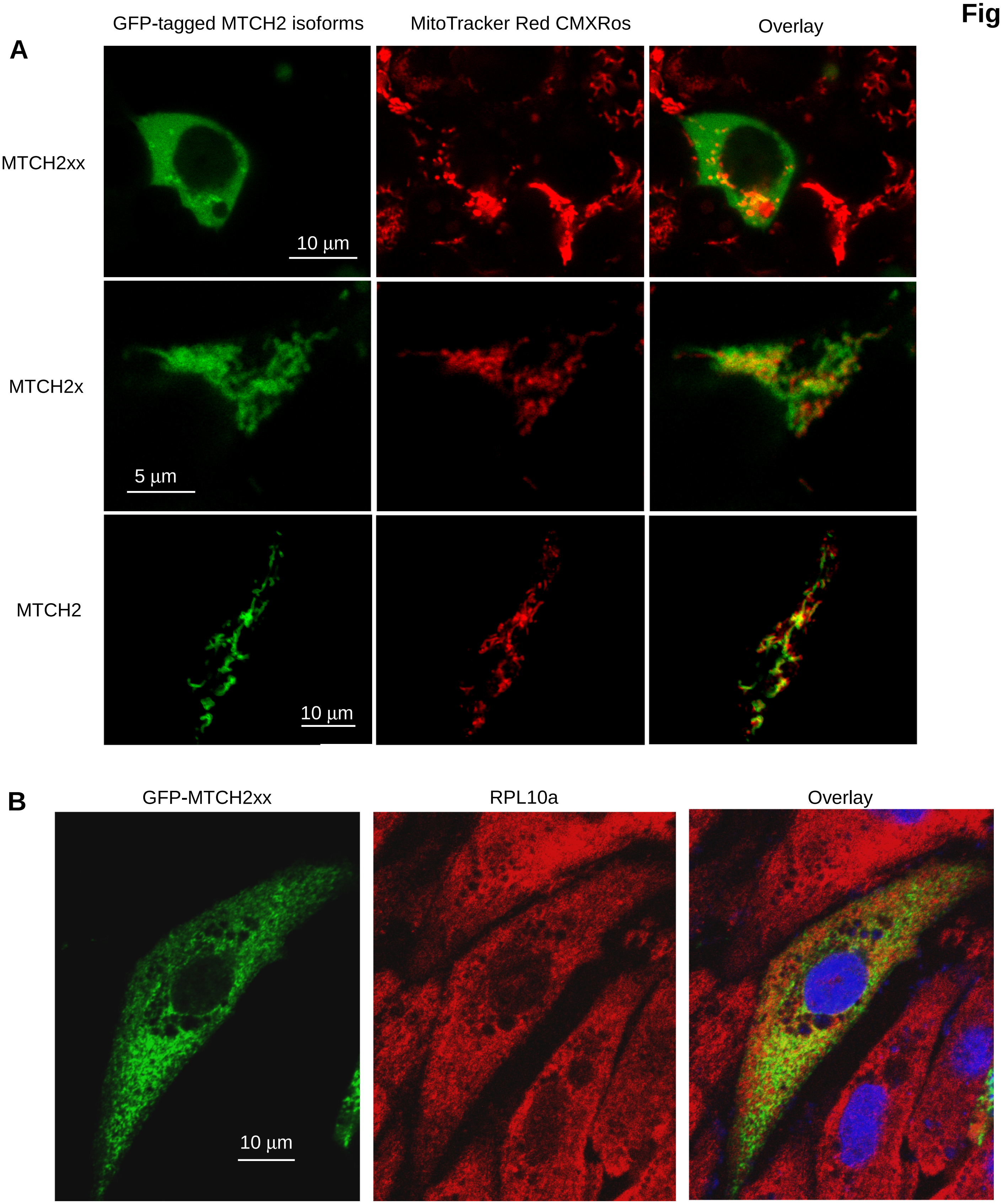

Figure S7. Differential cellular localization of MTCH2 isoforms.
(A) Confocal fluorescence microscopy images showing the cellular localization of GFP-tagged MTCH2 isoforms in HEK293 cells. Mitotracker Red CMXRos was used as mitochondrial marker. Percentage of GFP-positive cells in case of GFP-MTCH2xx was less in comparison with GFP-MTCH2 and GFP-MTCH2x.
(B) Confocal fluorescence microscopy images showing the cytoplasmic localization of exogenous GFP-tagged MTCH2xx in bovine aortic endothelial cells. Ribosomal protein L10a, which served as a cytoplasmic marker, was stained using a specific antibody (SC-10087, Santa Cruz Biotechnology).

Figure S8

A

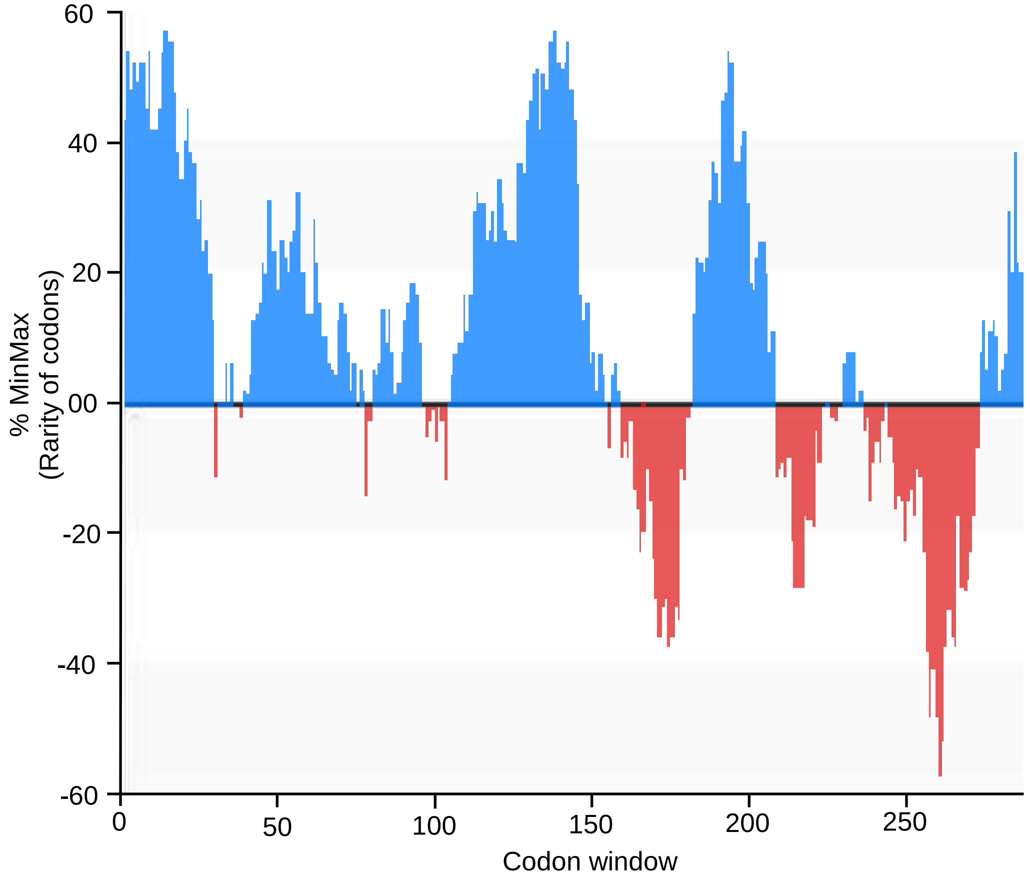

60
40
20
00
-20
-40
-60
% MinMax
(Rarity of codons)
0
50
100
150
200
250
Codon window

B

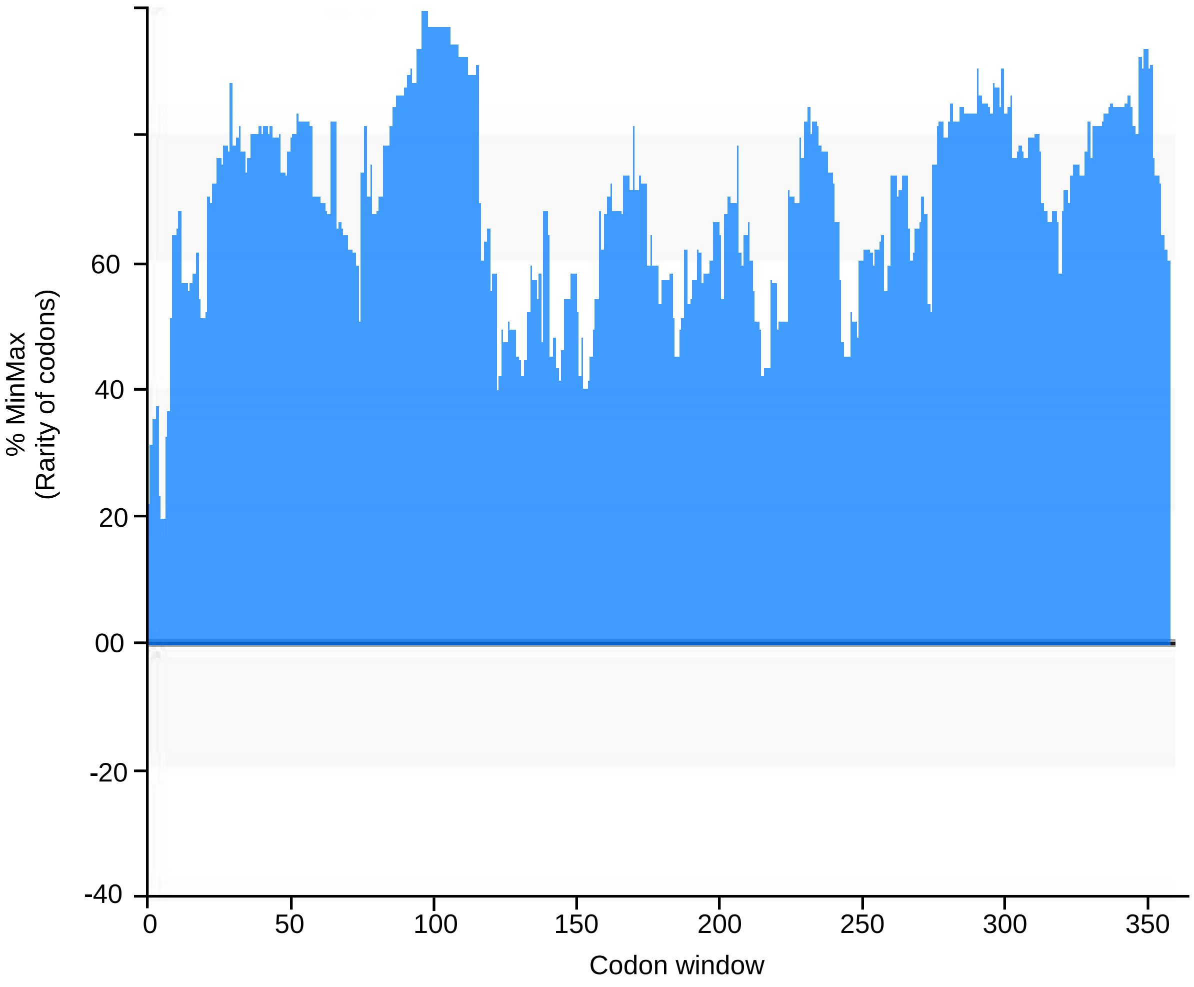

60
40
20
00
-20
-40
% MinMax
(Rarity of codons)
0
50
100
150
200
250
300
350
Codon window

Figure S8. Presence of clusters of rare codons upstream of the canonical stop codon of *MTCH2*.
(A) %MinMax algorithm was applied to the canonical coding sequence of *MTCH2* using 18-codon sliding window. %Max bars (blue) indicate clusters of commonly used codons and %Min bars (red) indicate clusters of rare codons.
(B) %MinMax algorithm applied to the canonical coding sequence of beta-actin is shown for comparison.
Rare codon calculator (http://www.codons.org/) was used for this analysis.

Table S1. List of *MTCH2*-derived peptides detected in proteomes of mouse organs

Peptide sequence	Source	Score
EYSSSFDR	Mouse brain[1]	61.999
MLISRCGAGTVTFLDSQCTELWER[#]		26.627
SAATLITHPFHVITLR		128.9
VLQYYQESEKPEELGSVTVQK		75.55
EYSSSFDR	Mouse brain[2]	79.809
GLFTGLTPR		134.81
GNSLFFR		113.08
LCSGVLGTVVHGK		100.19
MLITRCGAGTVTFLR[#]		28.297
QVCQLPGLFCYAQHIASIDGRR		69.122
SAATLITHPFHVITLR		95.347
TYCYDLR		135.35
VLIQVGYEPLPPTIGR		121.02
VLQYYQESEKPEELGSVTVQK		179.36
GLFTGLTPR	Mouse brain[2]	98.523
GNSLFFR		79.939
GNSLFFRK		38.962
LCSGVLGTVVHGK		66.498
MLITRCGAGTVTFLR[#]		28.297
QVCQLPGLFCYAQHIASIDGR		68.168
QVCQLPGLFCYAQHIASIDGRR		13.188
SAATLITHPFHVITLR		117.4
SMVQFIGR		139.92
VLIQVGYEPLPPTIGR		149.23
VLQYYQESEKPEELGSVTVQK		93.684
EYSSSFDR	Mouse brain[2]	82.241
GLFTGLTPR		43.808
GNSLFFR		49.358
LCSGVLGTVVHGK		19.496
MLISRCGAGTVTFLDSQCTELWER[#]		20.657
SAATLITHPFHVITLR		86.378
SMVQFIGR		58.981
TYCYDLR		43.246
VLIQVGYEPLPPTIGR		106.49
VLQYYQESEKPEELGSVTVQK		331.64
EYSSSFDR		82.241
GLFTGLTPR		43.808

SMVQFIGR	Mouse liver[3]	60.788
TYCYDLR		82.305
TYCYDLRMLIQR[@]		30.872
VLIQVGYEPLPPTIGR		134.99
VLQYYQESEKPEELGSVTVQK		211.57

[#]generated after double-SCR; specific to MTCH2xx isoform

[@]generated after single- or double-SCR; specific to MTCH2x or MTCH2xx isoform

REFRENCES

1. Hornburg, D. *et al.* Deep proteomic evaluation of primary and cell line motoneuron disease models delineates major differences in neuronal characteristics. *Mol. Cell. Proteom.* **13**, 3410-3420 (2014).
2. Sharma, K. *et al.* Cell type- and brain region-resolved mouse brain proteome. *Nat. Neurosci.* **18**, 1819-1831 (2015).
3. Azimifar, S.B., Nagaraj, N., Cox, J. & Mann, M. Cell-type-resolved quantitative proteomics of murine liver. *Cell Metab.* **20**, 1076-1087 (2014).
