# Supplementary material for: Stop codon read-through of mammalian MTCH2 leading to an unstable isoform regulates mitochondrial membrane potential
Source: J Biol Chem. 2020 Oct 7;295(50):17009–26. doi: 10.1074/jbc.RA120.014253 (PMC7863902; doi:10.1074/jbc.RA120.014253)

Fig 2A left

Sample	Replicate	Fluc	Rluc	Fluc/Rluc	Avg	SD
MTCH2 **-Stop-*Fluc***	1	45200	22700	1.991189427	2.048054473	0.053309325
	2	54100	25800	2.096899225		
	3	44000	21400	2.056074766		
MTCH2 **-Stop-ISR1+12^{ISR2}-*FLuc***	1	362000	37100	9.757412399	10.55370954	3.336405357
	2	295200	38400	7.6875		
	3	526000	37000	14.21621622		
MTCH2 **-Stop-Non specific-*Fluc***	1	12500	25700	0.486381323	0.368202038	0.102854804
	2	7600	23800	0.319327731		
	3	8130	27200	0.298897059		
MTCH2 **-No Stop-*FLuc***	1	833000	38700	21.5245478	17.79516187	4.883544479
	2	723000	36900	19.59349593		
	3	422000	34400	12.26744186		
**No *Fluc***	1	9	40600	0.000221675	0.000311009	9.76294E-05
	2	13	43900	0.000296128		
	3	12	28900	0.000415225		
**P value**						
MTCH2 -Stop-*Fluc* and *MTCH2* -Stop-ISR1+12^{ISR2}-*FLuc*	0.012					
MTCH2 -Stop-ISR1+12^{ISR2}-*Fluc* and *MTCH2* -Stop-Non specific-*Fluc*	0.006					

Fig 2A left

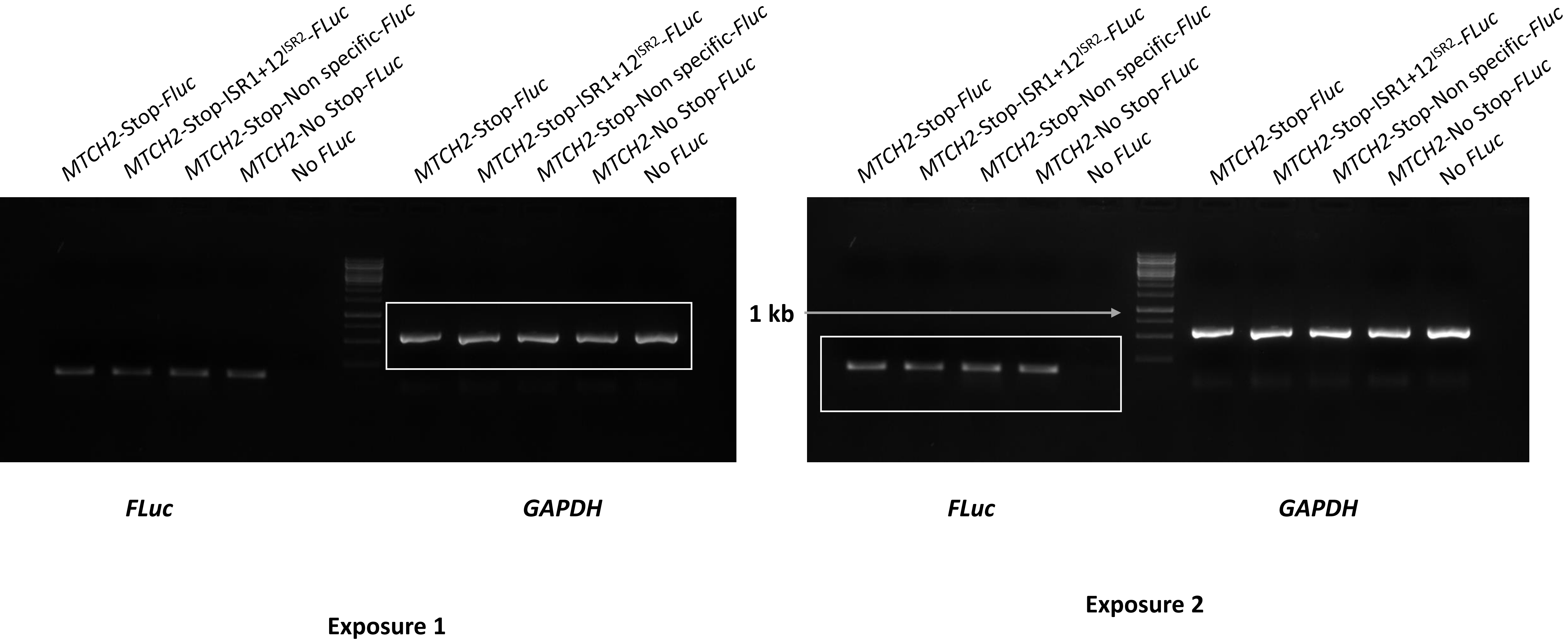

MTCH2-Stop-Fluc
MTCH2-Stop-ISR1+12^ISR2-FLuc
MTCH2-Stop-Non specific-Fluc
MTCH2-No Stop-FLuc
No FLuc
MTCH2-Stop-Fluc
MTCH2-Stop-ISR1+12^ISR2-FLuc
MTCH2-Stop-Non specific-Fluc
MTCH2-No Stop-FLuc
No FLuc
1 kb
FLuc
GAPDH
FLuc
GAPDH
Exposure 1
Exposure 2

Fig 2A right

Sample	Replicate	Fluc	Avg	SD
MTCH2* -Stop-*Fluc	1	39	54.66666667	18.44812547
	2	75		
	3	50		
MTCH2* -Stop-ISR1+12^{ISR2}-*FLuc	1	2619	2951.333333	350.1918522
	2	3317		
	3	2918		
MTCH2* -No Stop-*FLuc	1	7547	5486.666667	2215.629106
	2	5770		
	3	3143		
**P value**				
MTCH2 -Stop-*Fluc* and *MTCH2* -Stop-ISR1+12^{ISR2}-*FLuc*	0.005	Welch's correction applied		

Fig 2B top

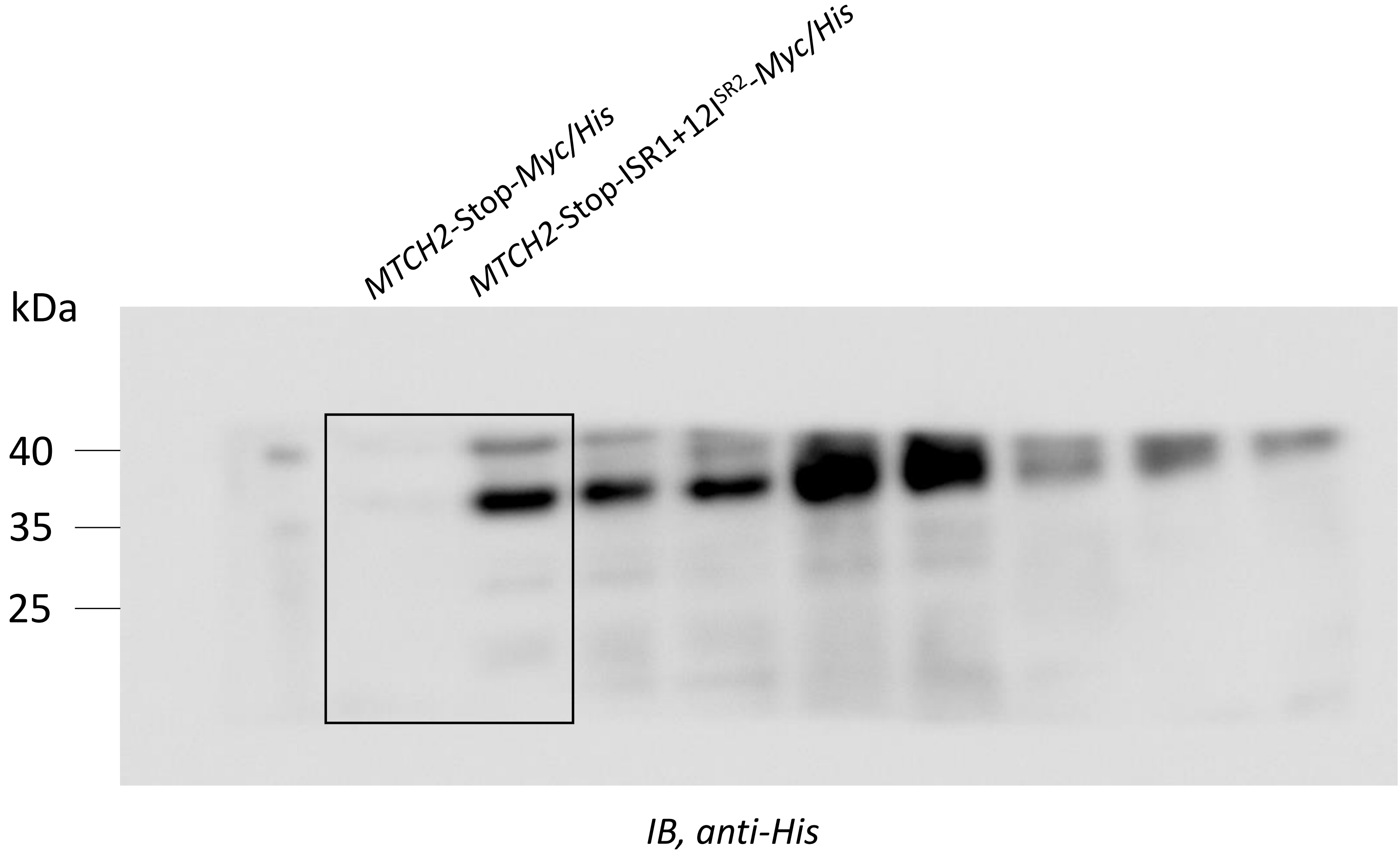
MTCH2-Stop-Myc/His
MTCH2-Stop-ISR1+12I^SR2-Myc/His
kDa
40
35
25
IB, anti-His

Fig 2B bottom

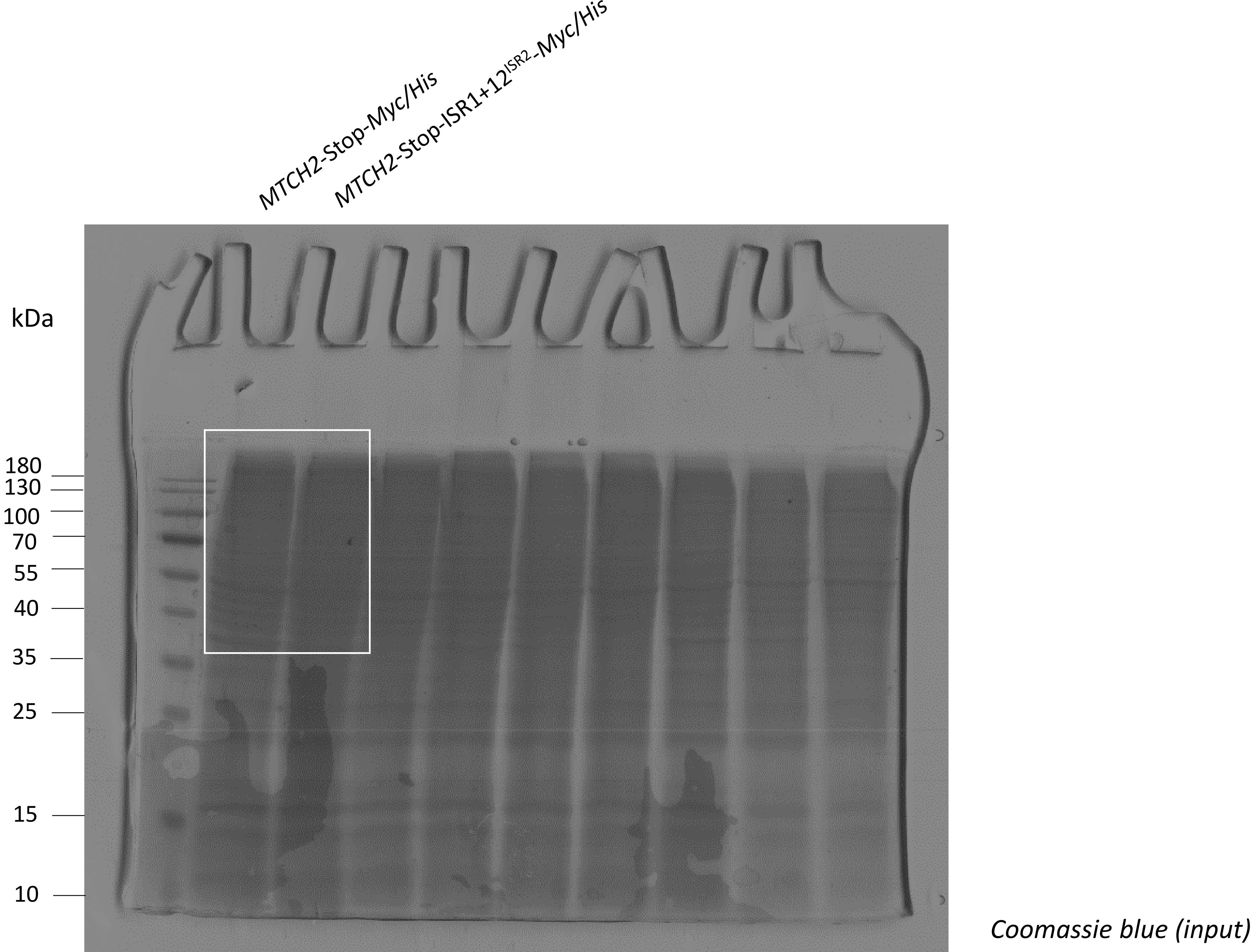

Fig 2C

Sample	Replicate	GFP MFI	RFP MFI	GFP/RFP	Avg	SD
MTCH2* -Stop-*GFP	1	1138.1	1078.8	1.054968484	1.112259838	0.089377815
	2	1038.3	973.5	1.066563945		
	3	1209.9	995.6	1.215247087		
MTCH2* -Stop-ISR1+12^{ISR2}-*GFP	1	10982.4	1491.9	7.361351297	7.734263723	0.380294958
	2	10440.4	1352.4	7.719905353		
	3	10458.1	1287.7	8.121534519		
MTCH2* -No Stop-*GFP	1	10754.6	905.1	11.88222296	12.27967012	0.9876024
	2	11243.1	973.2	11.5527127		
	3	11053	824.6	13.4040747		
**P value**						
MTCH2 -Stop-*GFP* and *MTCH2* -Stop-ISR1+12^{ISR2}-*GFP*	<0.0001					

Fig 2C

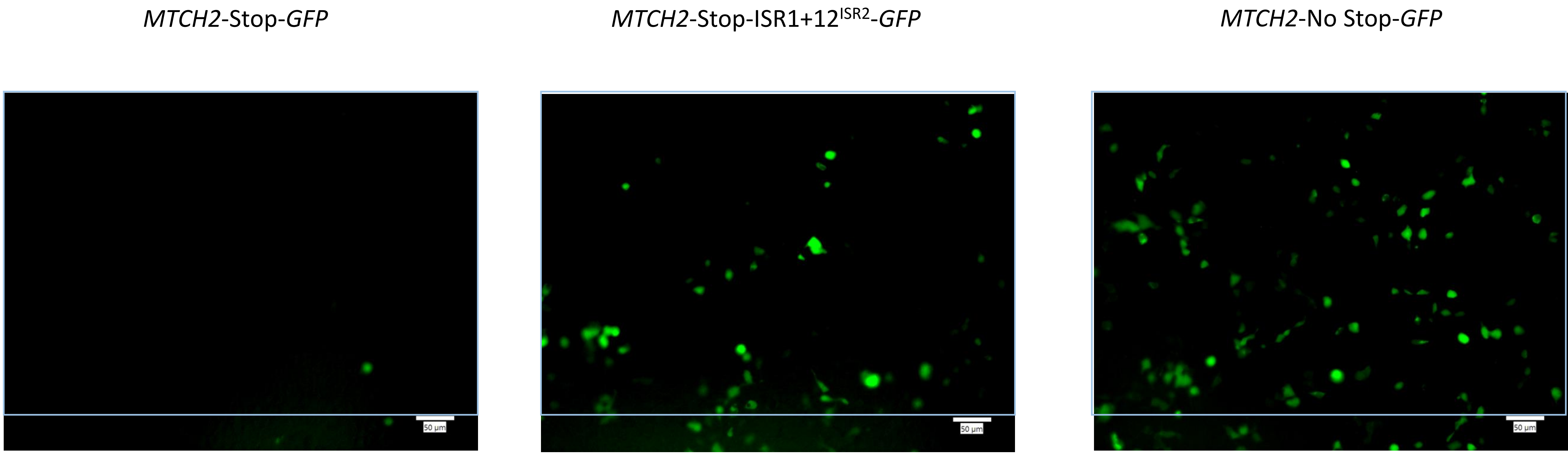

MTCH2-Stop-*GFP*
MTCH2-Stop-ISR1+12^{ISR2}-*GFP*
MTCH2-No Stop-*GFP*
50 μm
50 μm
50 μm

Fig 2D

Sample	Replicate	Fluc	Rluc	Fluc/Rluc	Avg	SD
Rluc* -Stop-non specific-*Fluc	1	1950	73700	0.026458616	0.025909391	0.001254471
	2	2210	90300	0.024473976		
	3	1940	72400	0.02679558		
Rluc* -Stop-ISR1+12^{ISR2}-*FLuc	1	1110	11400	0.097368421	0.093928456	0.009237474
	2	1060	12700	0.083464567		
	3	1060	10500	0.100952381		
Rluc*- No stop-ISR1+12^{ISR2}-*FLuc	1	341000	1080000	0.315740741	0.347855423	0.034618538
	2	333000	970000	0.343298969		
	3	333000	866000	0.384526559		
**P value**						
Rluc- Stop-non specific-*Fluc* and *Rluc*- Stop-ISR1+12^{ISR2}-*Fluc*		<0.001				

Fig 3A left

Sample	Replicate	Fluc	Rluc	Fluc/Rluc	Avg	SD
MTCH2* -Stop-ISR1-Stop-*Fluc	1	1830	91900	0.019912949	0.021895527	0.002563526
	2	2070	83500	0.024790419		
	3	1750	83400	0.020983213		
MTCH2* -Stop-ISR1-Stop-ISR2-*FLuc	1	7340	77400	0.094832041	0.107120687	0.013733271
	2	9780	80200	0.121945137		
	3	8440	80700	0.104584882		
MTCH2* -No Stop-*Fluc	1	14000	16800	0.833333333	0.995852703	0.14082265
	2	17200	15900	1.081761006		
	3	14800	13800	1.072463768		
**No *Fluc***	1	58	28200	0.002056738	0.001128328	0.000822155
	2	28	33500	0.000835821		
	3	13	26400	0.000492424		
P value						
MTCH2 -Stop-ISR1-Stop-*Fluc* and *MTCH2* -Stop-ISR1-Stop-ISR2-*Fluc*			<0.001			

Fig 3A left

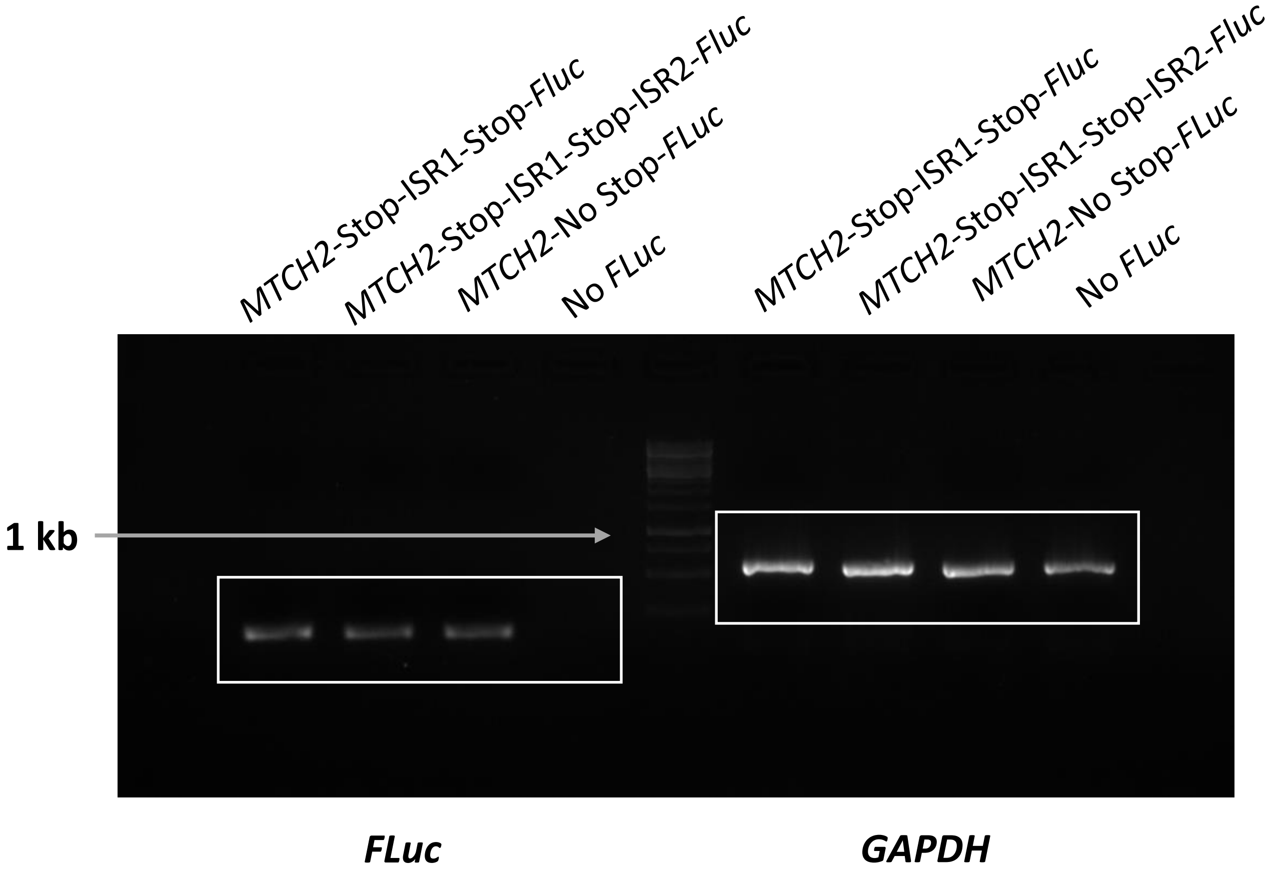
MTCH2-Stop-ISR1-Stop-Fluc
MTCH2-Stop-ISR1-Stop-ISR2-Fluc
MTCH2-No Stop-FLuc
No FLuc
MTCH2-Stop-ISR1-Stop-Fluc
MTCH2-Stop-ISR1-Stop-ISR2-Fluc
MTCH2-No Stop-FLuc
No FLuc
1 kb
FLuc
GAPDH

Fig 3A right

Sample	Replicate	Fluc	Avg	SD
MTCH2* -Stop-ISR1-Stop-*Fluc	1	109	173.6666667	88.86694174
	2	137		
	3	275		
MTCH2* -Stop-ISR1-Stop-ISR2-*FLuc	1	13900	11416.66667	2475.042087
	2	11400		
	3	8950		
MTCH2* -No Stop-*Fluc	1	73800	99266.66667	22077.43947
	2	111000		
	3	113000		
P value				
MTCH2 -Stop-ISR1-Stop-*Fluc* and *MTCH2* -Stop-ISR1-Stop-ISR2-*Fluc*		0.0158	Welch's correction applied	

Fig 3B

Sample	Replicate	GFP MFI	RFP MFI	GFP/RFP	Avg	SD
MTCH2* -Stop-ISR1-Stop-*GFP	1	170.133333	477.7	0.356151001	0.360166881	0.007013245
	2	169.733333	460.9	0.368264988		
	3	157.033333	441	0.356084655		
MTCH2* -Stop-ISR1-Stop-ISR2-*GFP	1	939.633333	486.6	1.931017947	1.719830229	0.193698769
	2	843.133333	543.8	1.550447468		
	3	1137.533333	677.9	1.678025274		
MTCH2* -No Stop-*GFP	1	14985.63333	1004.2	14.92295691	14.93940516	1.056443265
	2	14446.93333	1040	13.89128205		
	3	14622.83333	913.7	16.00397651		
P value						
MTCH2 -Stop-ISR1-Stop-*GFP* and *MTCH2* -Stop-ISR1-Stop-ISR2-*GFP*			0.0003			

Fig 3B

MTCH2-Stop-ISR1-Stop-*GFP* | *MTCH2*-Stop-ISR1-Stop-ISR2-*GFP* | *MTCH2*-No Stop-*GFP*

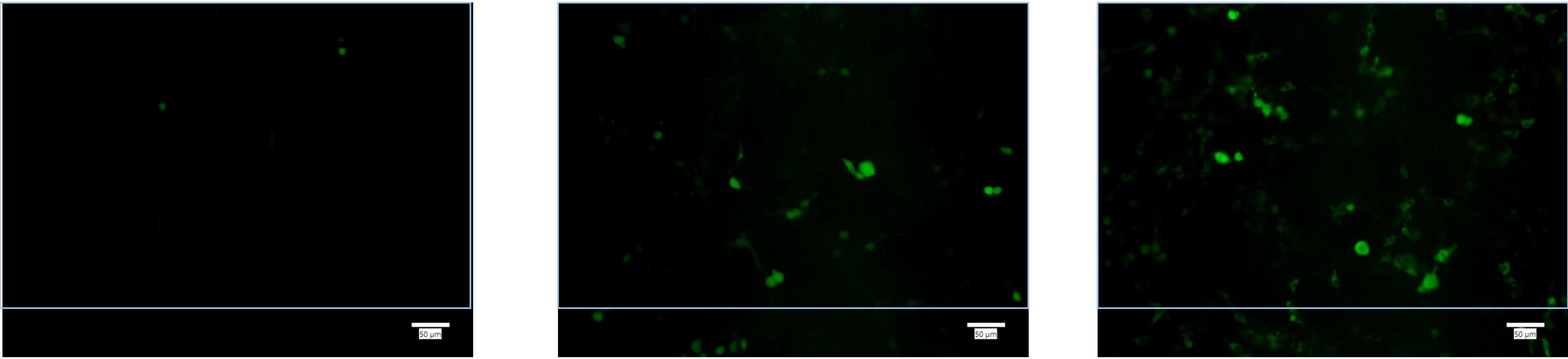

Fig 3C

Sample	Replicate	Fluc	Rluc	Fluc/Rluc	Avg	SD
Rluc* -Stop-ISR1-Stop-*Fluc	1	384	56800	0.006760563	0.004582753	0.002673198
	2	340	63100	0.005388273		
	3	111	69400	0.001599424		
Rluc* -Stop-non specific-*Fluc	1	2060	424000	0.004858491	0.004348468	0.000511443
	2	2180	501000	0.004351297		
	3	1960	511000	0.003835616		
Rluc* -Stop-ISR1-Stop-ISR2-*FLuc	1	932	62900	0.01481717	0.014737722	0.001189018
	2	862	63800	0.013510972		
	3	1050	66100	0.015885023		
Rluc* -Stop-ISR1+12^{ISR2}-*FLuc	1	3020	49500	0.061010101	0.055948159	0.00714586
	2	2790	58400	0.047773973		
	3	2640	44700	0.059060403		
Rluc* -No stop-ISR1+12^{ISR2}-*FLuc	1	539000	2090000	0.257894737	0.288646905	0.029481087
	2	570000	1800000	0.316666667		
	3	676000	2320000	0.29137931		
P value						
Rluc -Stop-ISR1-Stop-*Fluc* and *Rluc* -Stop-ISR1-Stop-ISR2-*Fluc*		0.0039				
Rluc- Stop-non specific-*Fluc* and *Rluc* -Stop-ISR1-Stop-ISR2-*Fluc*		0.001	Welch's correction applied			
Rluc- Stop-non specific-*Fluc* and *Rluc*- Stop-ISR1+12^{ISR2}-*FLuc*		0.0002				

Fig 4A

Sample	Replicate	Fluc	Rluc	Fluc/Rluc	Avg	SD
MTCH2* -Stop-*Fluc	1	1310	1270000	0.001031496	0.000696405	0.000335625
	2	634	909000	0.00069747		
	3	348	966000	0.000360248		
MTCH2* -Stop-ISR1+12^{ISR2} (WT)-*FLuc	1	8340	841000	0.009916766	0.013560588	0.003251991
	2	17300	1070000	0.016168224		
	3	18100	1240000	0.014596774		
MTCH2* -Stop-ISR1+12^{ISR2} (AU-CG)-*FLuc	1	2260	1310000	0.001725191	0.000762901	0.000858527
	2	532	1090000	0.000488073		
	3	86	1140000	7.54386E-05		
MTCH2* -Stop-ISR1+12^{ISR2} (CC-GG)-*FLuc	1	4380	1070000	0.004093458	0.004627088	0.000535764
	2	4540	879000	0.00516496		
	3	4290	928000	0.004622845		
MTCH2* -No Stop-*FLuc	1	29400	824000	0.035679612	0.035383596	0.000657741
	2	26200	731000	0.035841313		
	3	27600	797000	0.034629862		
**No *FLuc***	1	61	192000	0.000317708	0.000267103	7.55325E-05
	2	64	211000	0.000303318		
	3	64	355000	0.000180282		
P value						
MTCH2 -Stop-ISR1+12^{ISR2} (WT)-*Fluc* and *MTCH2* -Stop-ISR1+12^{ISR2} (AU-CG)-*FLuc*			0.003			
MTCH2 -Stop-ISR1+12^{ISR2} (WT)-*Fluc* and *MTCH2* -Stop-ISR1+12^{ISR2} (CC-GG)-*FLuc*			0.009			

Fig 4A

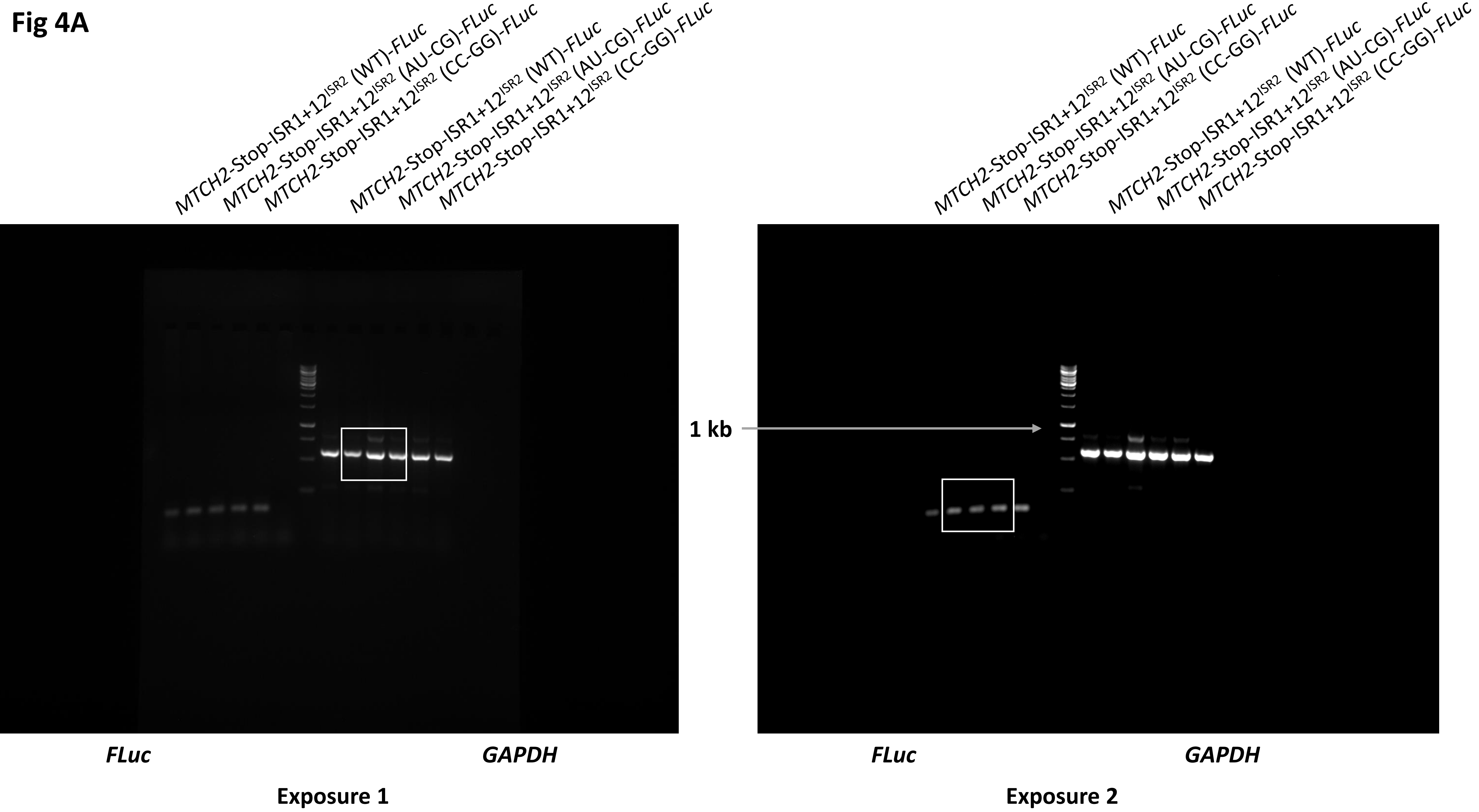
MTCH2-Stop-ISR1+12^{ISR2} (WT)-*FLuc*
MTCH2-Stop-ISR1+12^{ISR2} (AU-CG)-*FLuc*
MTCH2-Stop-ISR1+12^{ISR2} (CC-GG)-*FLuc*
MTCH2-Stop-ISR1+12^{ISR2} (WT)-*FLuc*
MTCH2-Stop-ISR1+12^{ISR2} (AU-CG)-*FLuc*
MTCH2-Stop-ISR1+12^{ISR2} (CC-GG)-*FLuc*
MTCH2-Stop-ISR1+12^{ISR2} (WT)-*FLuc*
MTCH2-Stop-ISR1+12^{ISR2} (AU-CG)-*FLuc*
MTCH2-Stop-ISR1+12^{ISR2} (CC-GG)-*FLuc*
MTCH2-Stop-ISR1+12^{ISR2} (WT)-*FLuc*
MTCH2-Stop-ISR1+12^{ISR2} (AU-CG)-*FLuc*
MTCH2-Stop-ISR1+12^{ISR2} (CC-GG)-*FLuc*
1 kb
FLuc
GAPDH
Exposure 1
FLuc
GAPDH
Exposure 2

Fig 4B

Sample	Replicate	Fluc	Rluc	Fluc/Rluc	Avg	SD
MTCH2* -Stop-*Fluc	1	476	285000	0.001670175	0.001987312	0.000281436
	2	469	225000	0.002084444		
	3	362	164000	0.002207317		
MTCH2* -Stop-ISR1+12^{ISR2}-*FLuc	1	17700	275000	0.064363636	0.06085084	0.004467126
	2	17400	279000	0.062365591		
	3	13900	249000	0.055823293		
MTCH2* -Stop+12^{ISR2}-*FLuc	1	22200	326000	0.06809816	0.069119123	0.001209257
	2	24800	352000	0.070454545		
	3	23600	343000	0.068804665		
MTCH2* -Stop-Non specific-*Fluc	1	198	260000	0.000761538	0.000806828	6.04996E-05
	2	204	233000	0.000875536		
	3	170	217000	0.00078341		
MTCH2* -No Stop-*FLuc	1	18000	171000	0.105263158	0.118811329	0.016649646
	2	16900	123000	0.137398374		
	3	19000	167000	0.113772455		
P value						
MTCH2 -Stop-*Fluc* and *MTCH2* -Stop-ISR1+12^{ISR2}-*FLuc*		<0.001				
MTCH2 -Stop-*Fluc* and *MTCH2* -Stop+12^{ISR2}-*FLuc*		<0.001				
MTCH2 -Stop+12^{ISR2}-*Fluc* and *MTCH2* -Non specific-*Fluc*		<0.001				

Fig 4B

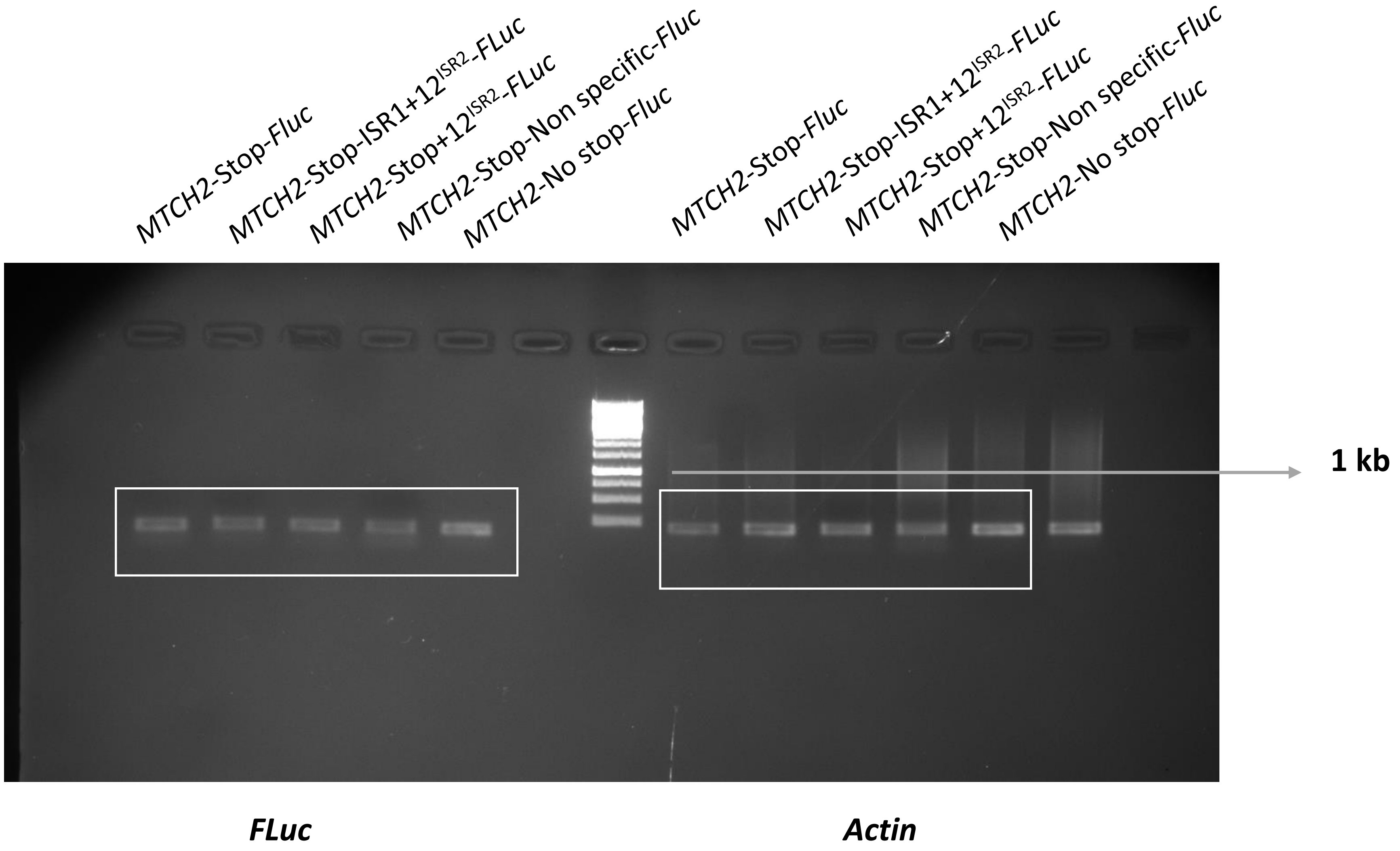

MTCH2-Stop-Fluc
MTCH2-Stop-ISR1+12ISR2-FLuc
MTCH2-Stop+12ISR2-FLuc
MTCH2-Stop-Non specific-Fluc
MTCH2-No stop-Fluc
MTCH2-Stop-Fluc
MTCH2-Stop-ISR1+12ISR2-FLuc
MTCH2-Stop+12ISR2-FLuc
MTCH2-Stop-Non specific-Fluc
MTCH2-No stop-Fluc
1 kb
FLuc
Actin

Fig 4C

Sample	Replicate	Fluc	Rluc	Fluc/Rluc	Avg	SD
MTCH2* -Stop-ISR1-Stop-*Fluc	1	2620	6810	0.384728341	0.389873363	0.038611302
	2	1990	5620	0.354092527		
	3	2210	5130	0.43079922		
MTCH2* -Stop-ISR1-Stop-12^{ISR2}-*FLuc	1	35200	3330	10.57057057	8.780863016	2.313570607
	2	42500	6890	6.168359942		
	3	31500	3280	9.603658537		
P value	0.003					

Fig 4C

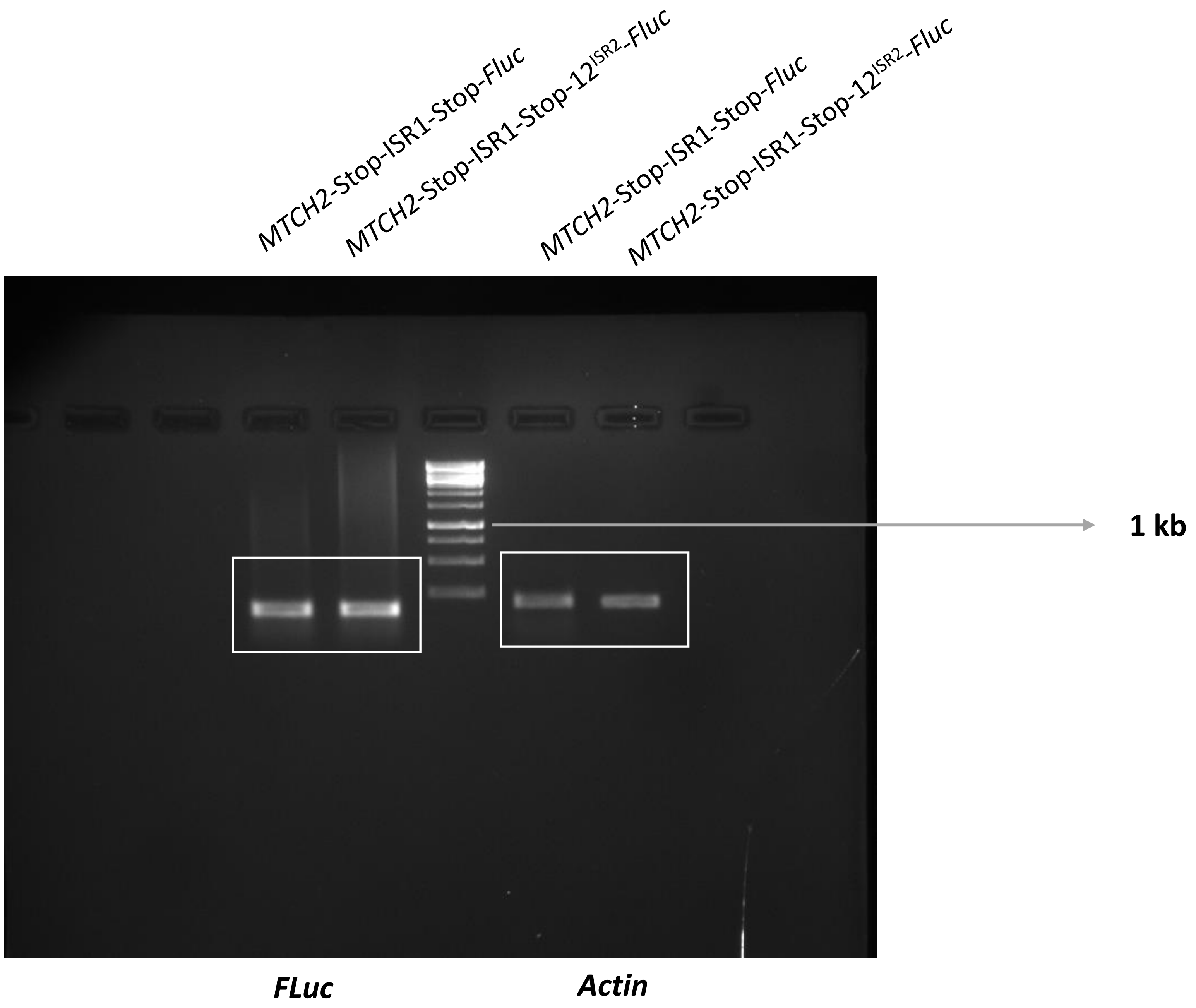

MTCH2-Stop-ISR1-Stop-Fluc
MTCH2-Stop-ISR1-Stop-12^{ISR2}-Fluc
MTCH2-Stop-ISR1-Stop-Fluc
MTCH2-Stop-ISR1-Stop-12^{ISR2}-Fluc
1 kb
FLuc
Actin

Fig 4D

Sample	Replicate	Fluc	Rluc	Fluc/Rluc	Avg	SD
Rluc* -Stop-Non specific-*Fluc	1	254	187000	0.001358289	0.00181046	0.00043405
	2	270	146000	0.001849315		
	3	318	143000	0.002223776		
RLuc* -Stop+12^{ISR2}-*FLuc	1	1080	173000	0.006242775	0.007183852	0.001220733
	2	1490	174000	0.008563218		
	3	1140	169000	0.006745562		
RLuc* -Stop-ISR1+12^{ISR2}-*FLuc	1	1050	120000	0.00875	0.009382699	0.000559318
	2	1040	106000	0.009811321		
	3	1160	121000	0.009586777		
RLuc* -*MTCH2* -Stop-ISR1+12^{ISR2}-*FLuc	1	808	13600	0.059411765	0.068147176	0.018760572
	2	797	14400	0.055347222		
	3	1130	12600	0.08968254		
RLuc* -NoStop-*FLuc	1	374000	985000	0.379695431	0.321334454	0.078277173
	2	347000	986000	0.351926978		
	3	244000	1050000	0.232380952		
P value						
Rluc -Stop-Non specific-*Fluc* and *RLuc* -Stop+12^{ISR2}-*FLuc*			0.002			
RLuc -Stop+12^{ISR2}-*Fluc* and *RLuc-MTCH2* -Stop-ISR1+12^{ISR2}-*FLuc*			0.005			

Fig 5A

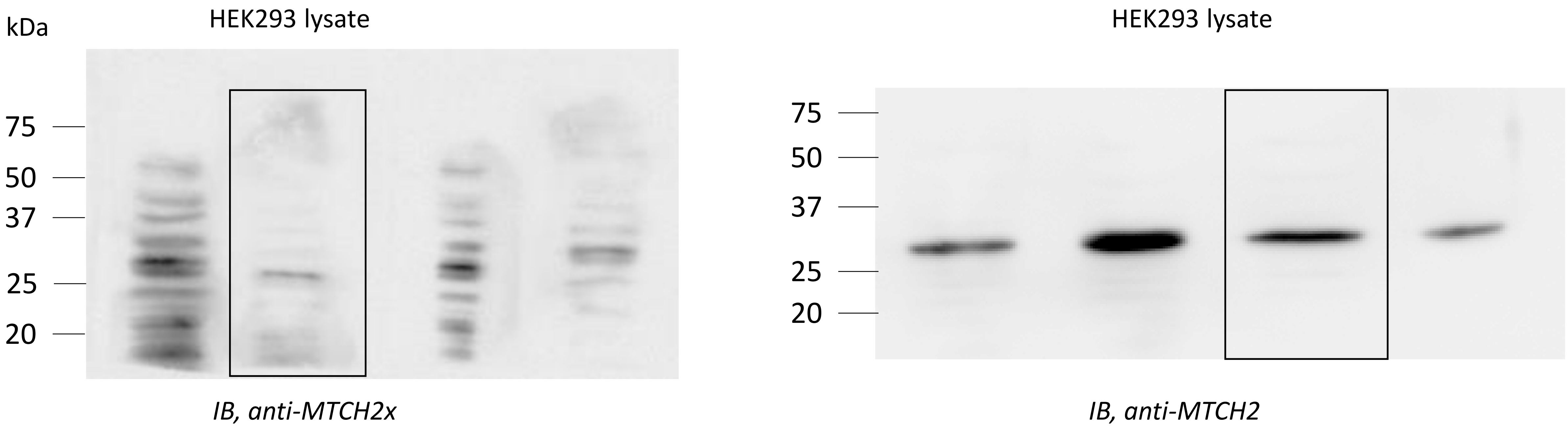
kDa
HEK293 lysate
75
50
37
25
20
IB, anti-MTCH2x
HEK293 lysate
75
50
37
25
20
IB, anti-MTCH2

Fig 5B

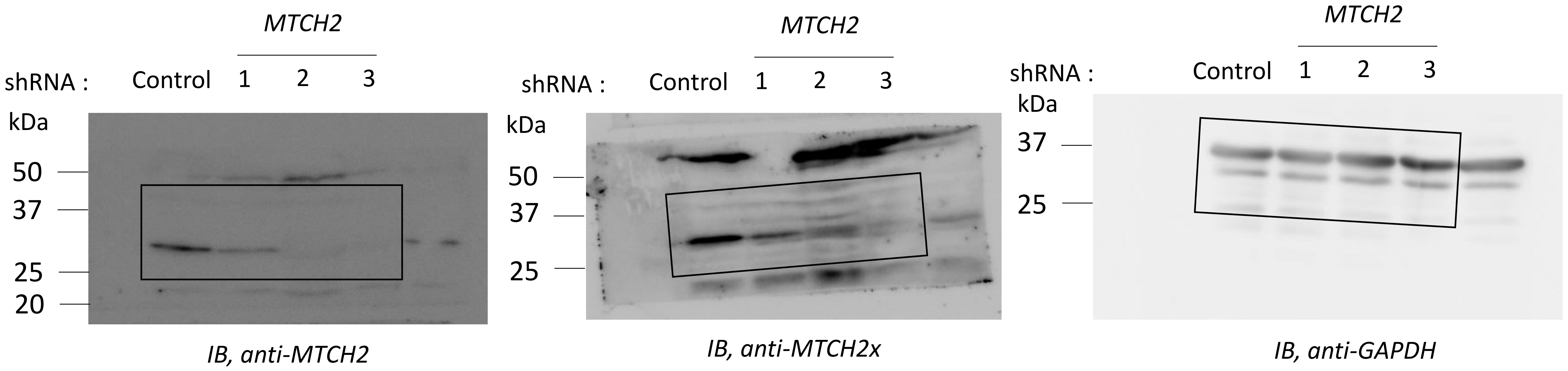
MTCH2
shRNA :
Control
1
2
3
kDa
50
37
25
20
IB, anti-MTCH2
MTCH2
shRNA :
Control
1
2
3
kDa
50
37
25
IB, anti-MTCH2x
MTCH2
shRNA :
Control
1
2
3
kDa
37
25
IB, anti-GAPDH

Fig 5C

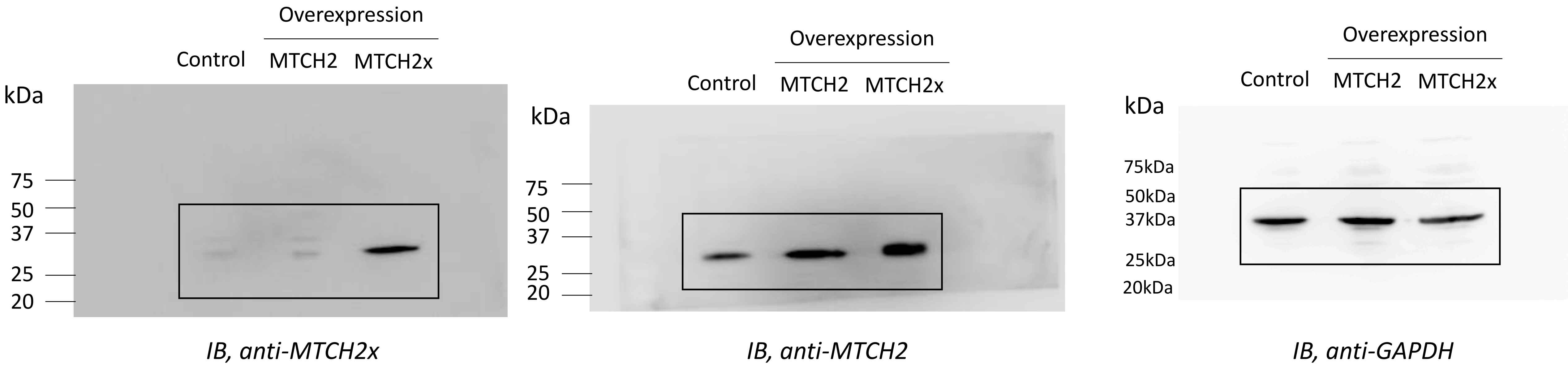
Overexpression
Control
MTCH2
MTCH2x
kDa
75
50
37
25
20
IB, anti-MTCH2x
Overexpression
Control
MTCH2
MTCH2x
kDa
75
50
37
25
20
IB, anti-MTCH2
Overexpression
Control
MTCH2
MTCH2x
kDa
75kDa
50kDa
37kDa
25kDa
20kDa
IB, anti-GAPDH

Fig 5D

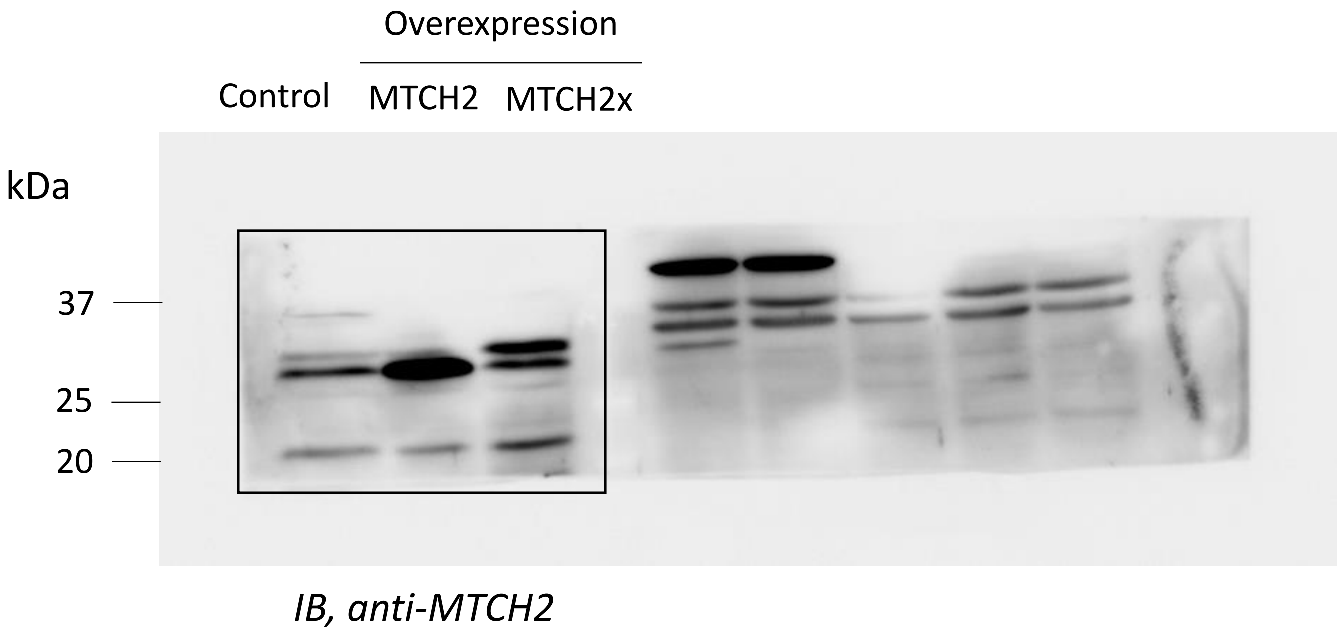
Overexpression
Control
MTCH2
MTCH2x
kDa
37
25
20
IB, anti-MTCH2

Fig 5D

MTCH2 isoform	Replicate	% of total MTCH2	Average	SD
MTCH2	1	71.89799287	77.51105396	6.419683121
	2	76.12443649		
	3	84.51073252		
MTCH2x	1	28.10200713	22.48894604	6.419683121
	2	23.87556351		
	3	15.48926748		

Fig 5E

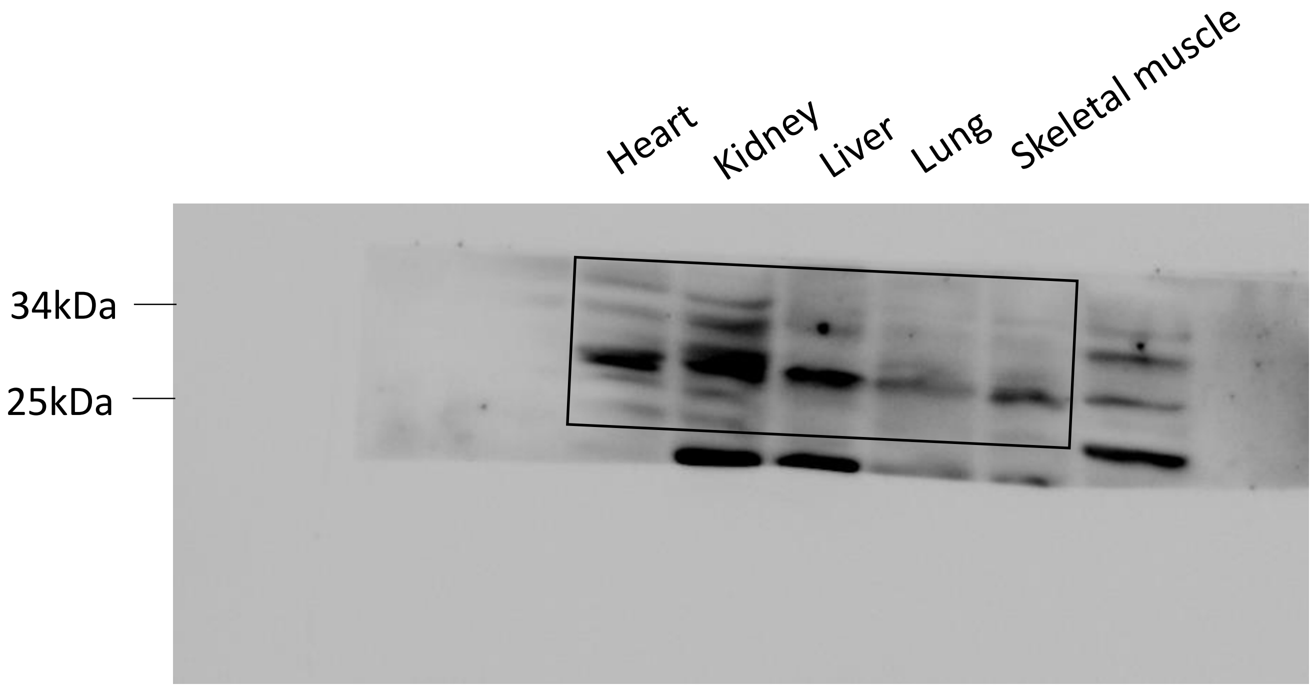

IB, anti-MTCH2x

Fig 6A

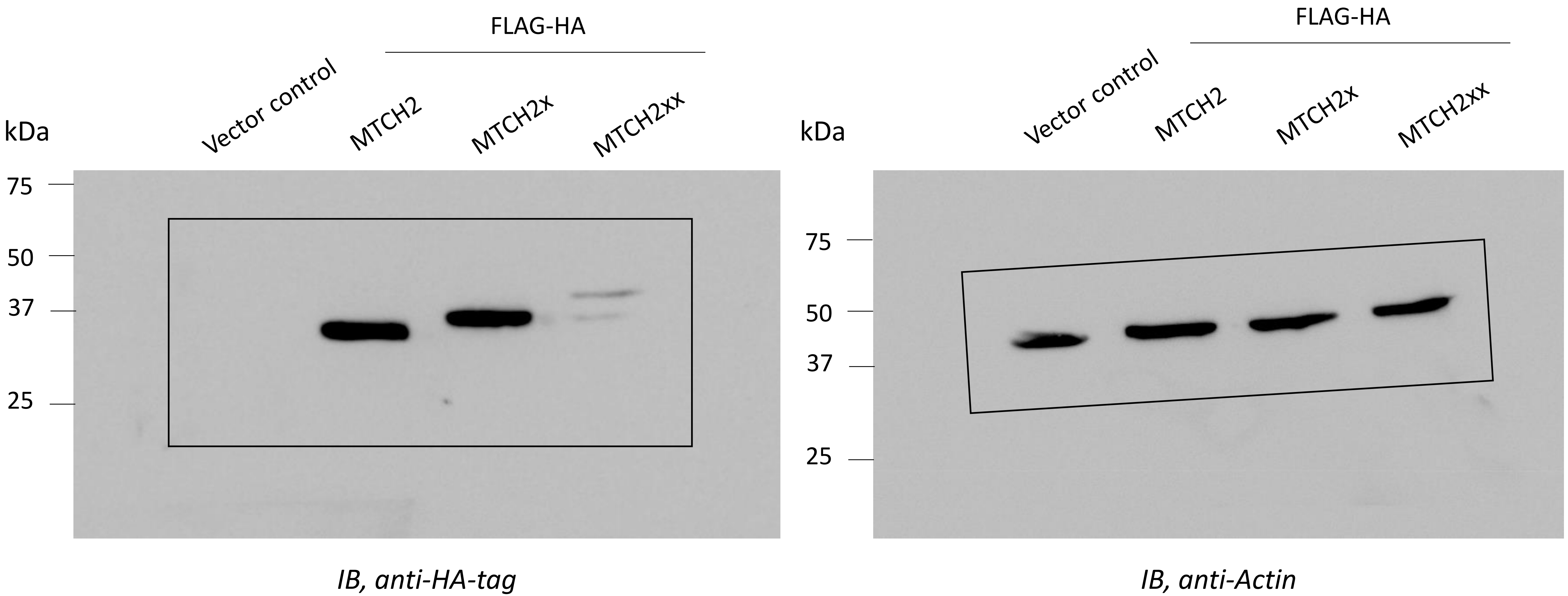
FLAG-HA
Vector control
MTCH2
MTCH2x
MTCH2xx
kDa
75
50
37
25
IB, anti-HA-tag
FLAG-HA
Vector control
MTCH2
MTCH2x
MTCH2xx
kDa
75
50
37
25
IB, anti-Actin

Fig 6A

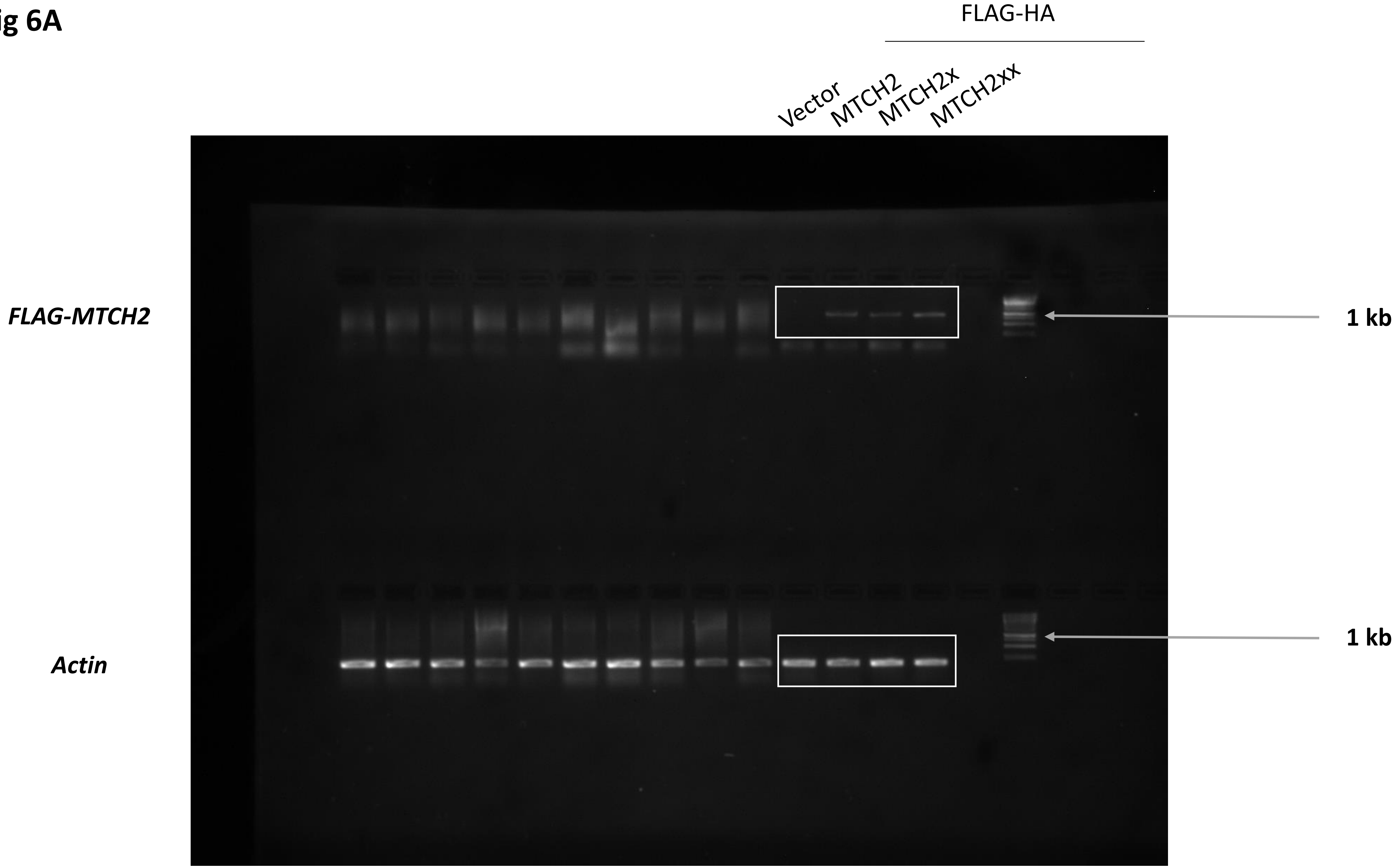
FLAG-HA
Vector
MTCH2
MTCH2x
MTCH2xx
FLAG-MTCH2
Actin
1 kb
1 kb

Fig 6B

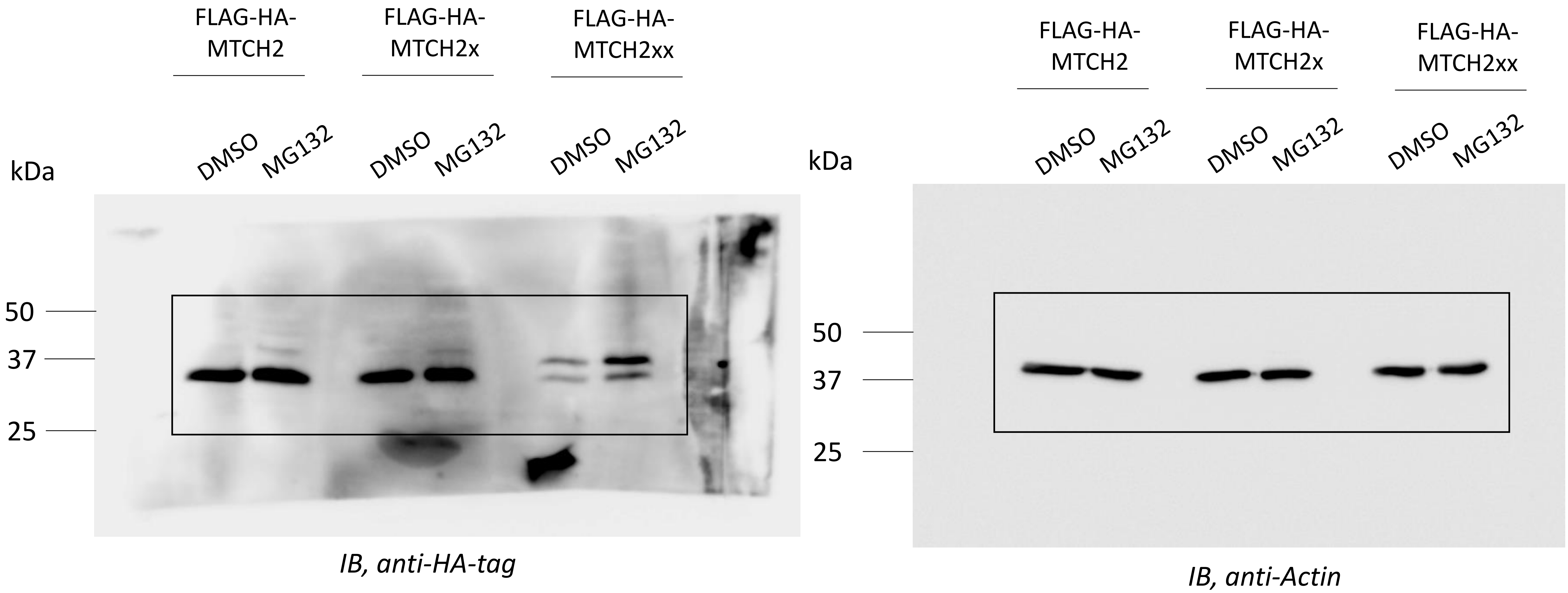
FLAG-HA-MTCH2
FLAG-HA-MTCH2x
FLAG-HA-MTCH2xx
DMSO
MG132
DMSO
MG132
DMSO
MG132
kDa
50
37
25
IB, anti-HA-tag
FLAG-HA-MTCH2
FLAG-HA-MTCH2x
FLAG-HA-MTCH2xx
DMSO
MG132
DMSO
MG132
DMSO
MG132
kDa
50
37
25
IB, anti-Actin

Fig 6C

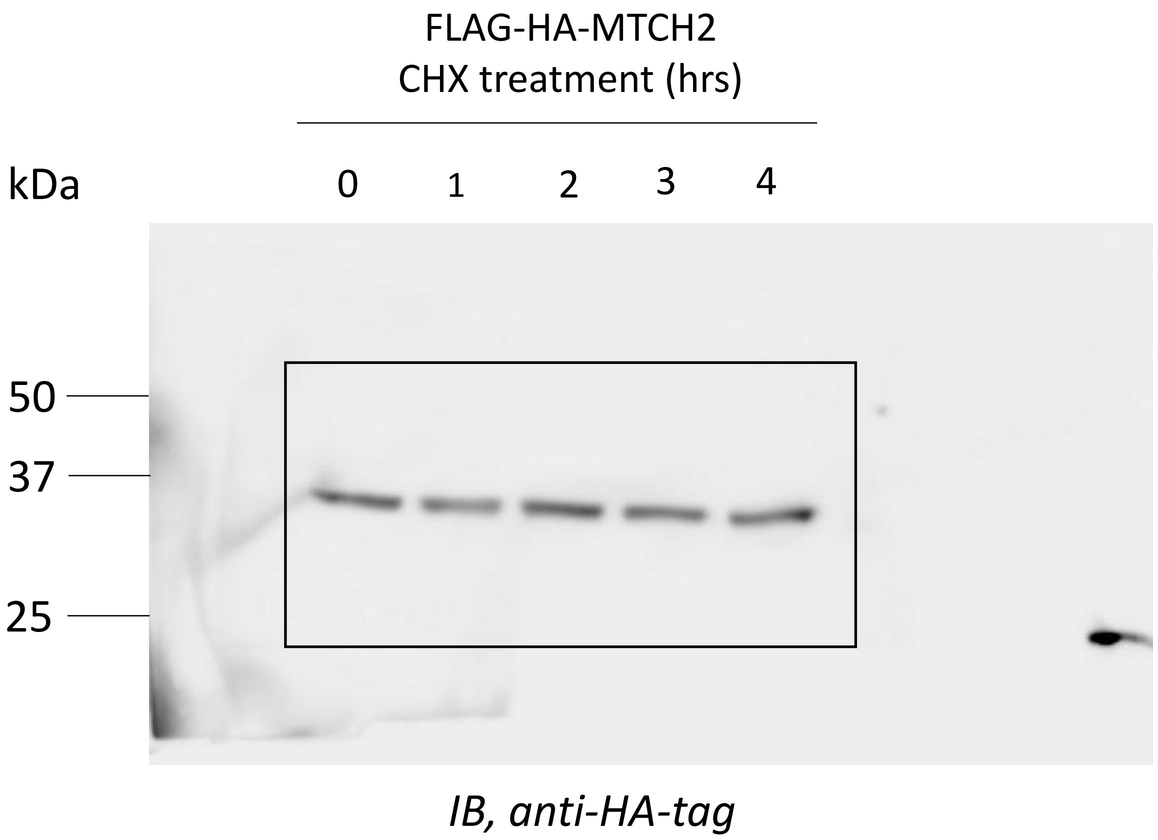
FLAG-HA-MTCH2
CHX treatment (hrs)
kDa
0
1
2
3
4
50
37
25
IB, anti-HA-tag

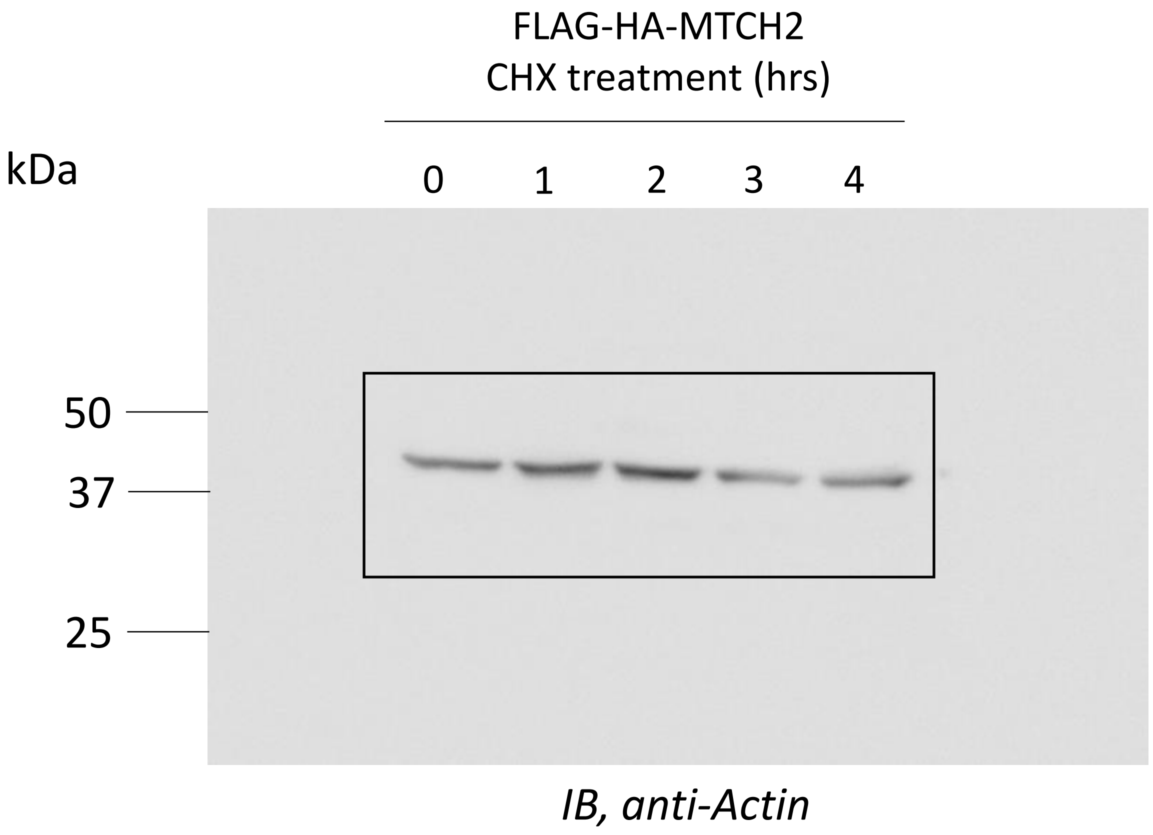
FLAG-HA-MTCH2
CHX treatment (hrs)
kDa
0
1
2
3
4
50
37
25
IB, anti-Actin

Fig 6C

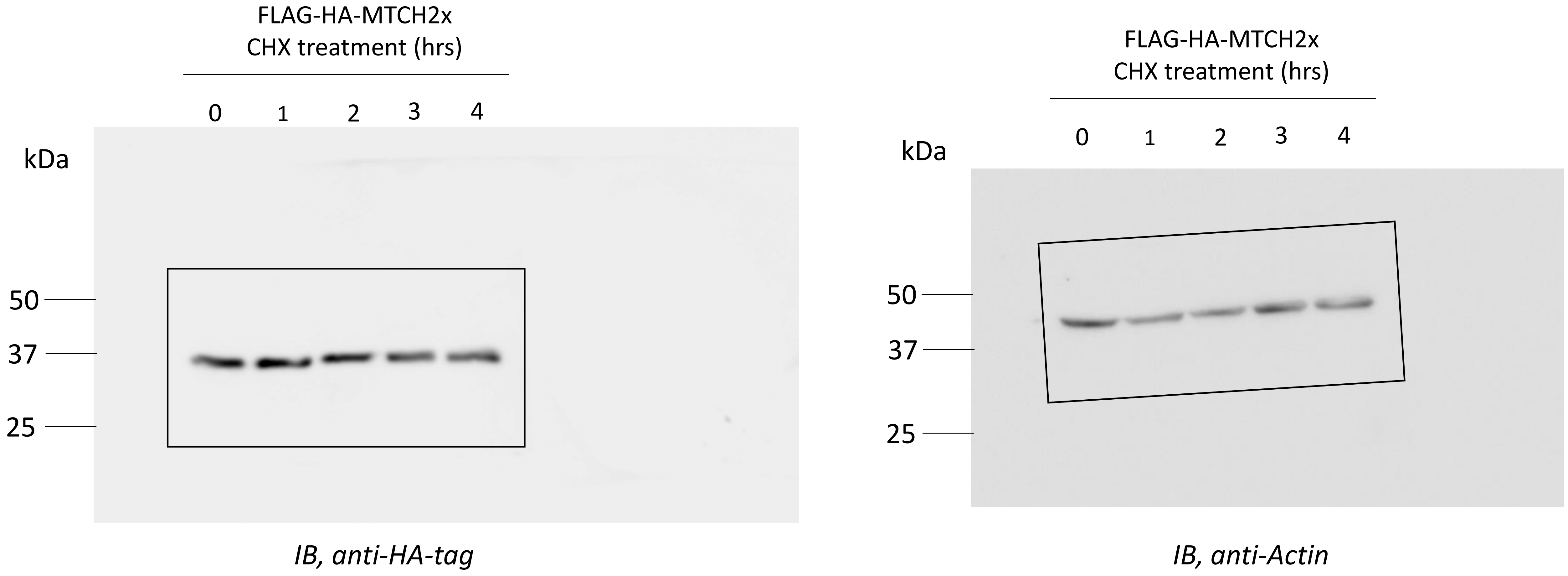
FLAG-HA-MTCH2x
CHX treatment (hrs)
0
1
2
3
4
kDa
50
37
25
IB, anti-HA-tag
FLAG-HA-MTCH2x
CHX treatment (hrs)
0
1
2
3
4
kDa
50
37
25
IB, anti-Actin

Fig 6C

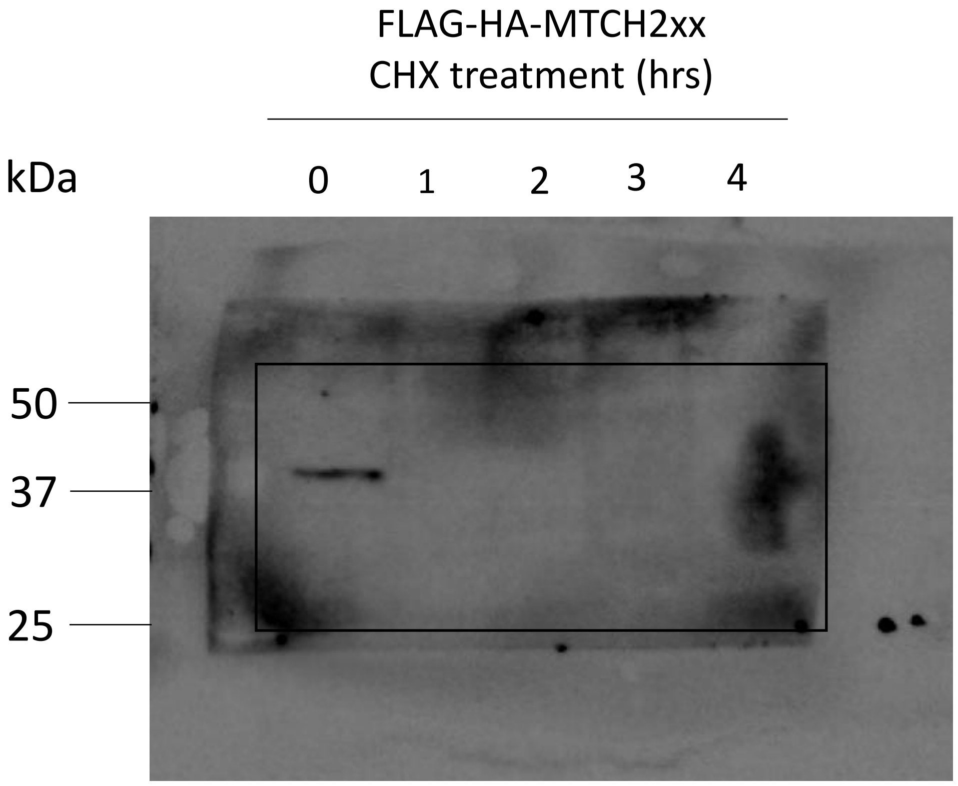

IB, anti-HA-tag

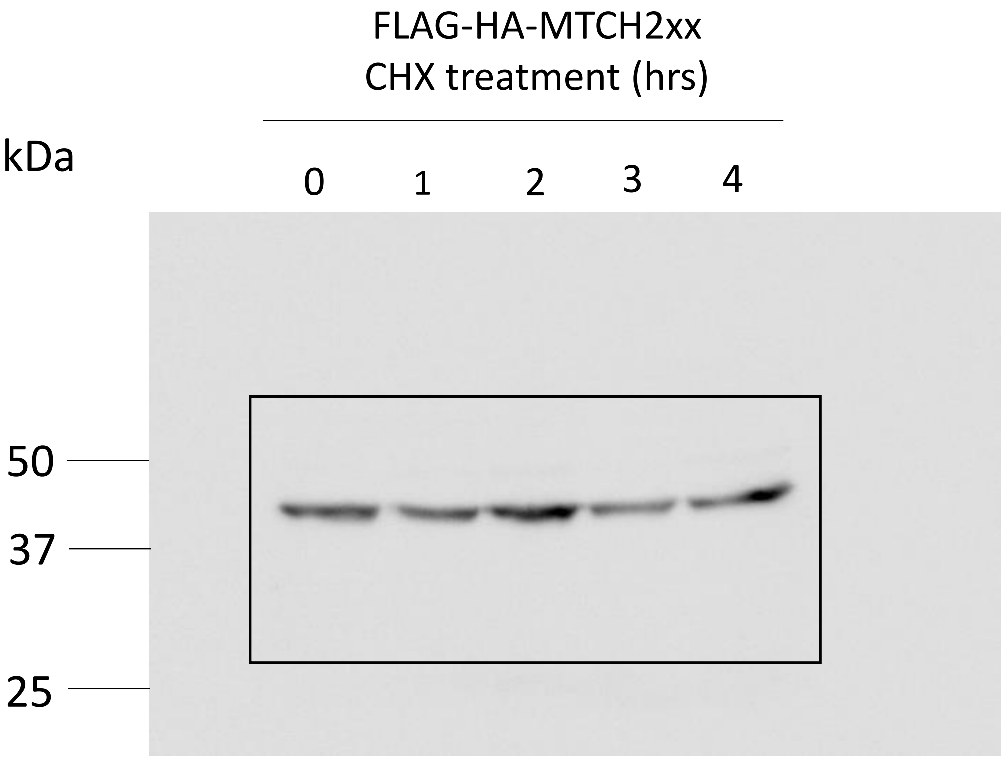

IB, anti-Actin

Fig 6D

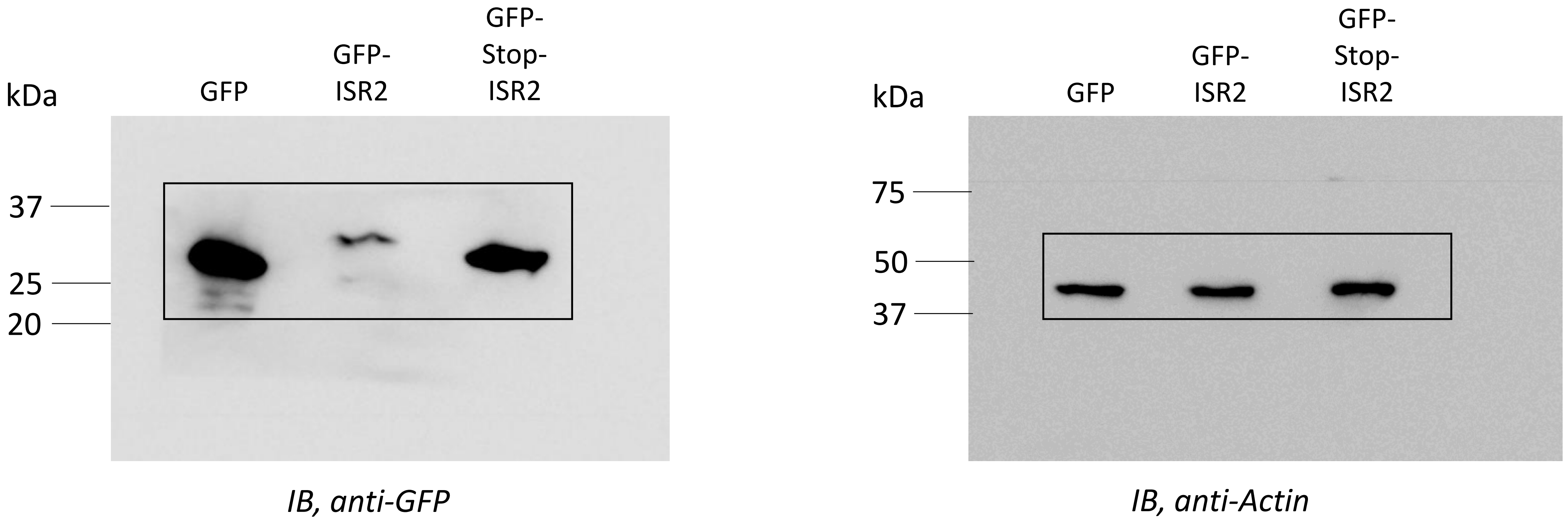

kDa
GFP
GFP-ISR2
GFP-Stop-ISR2
37
25
20
IB, anti-GFP
kDa
GFP
GFP-ISR2
GFP-Stop-ISR2
75
50
37
IB, anti-Actin

Fig 6D

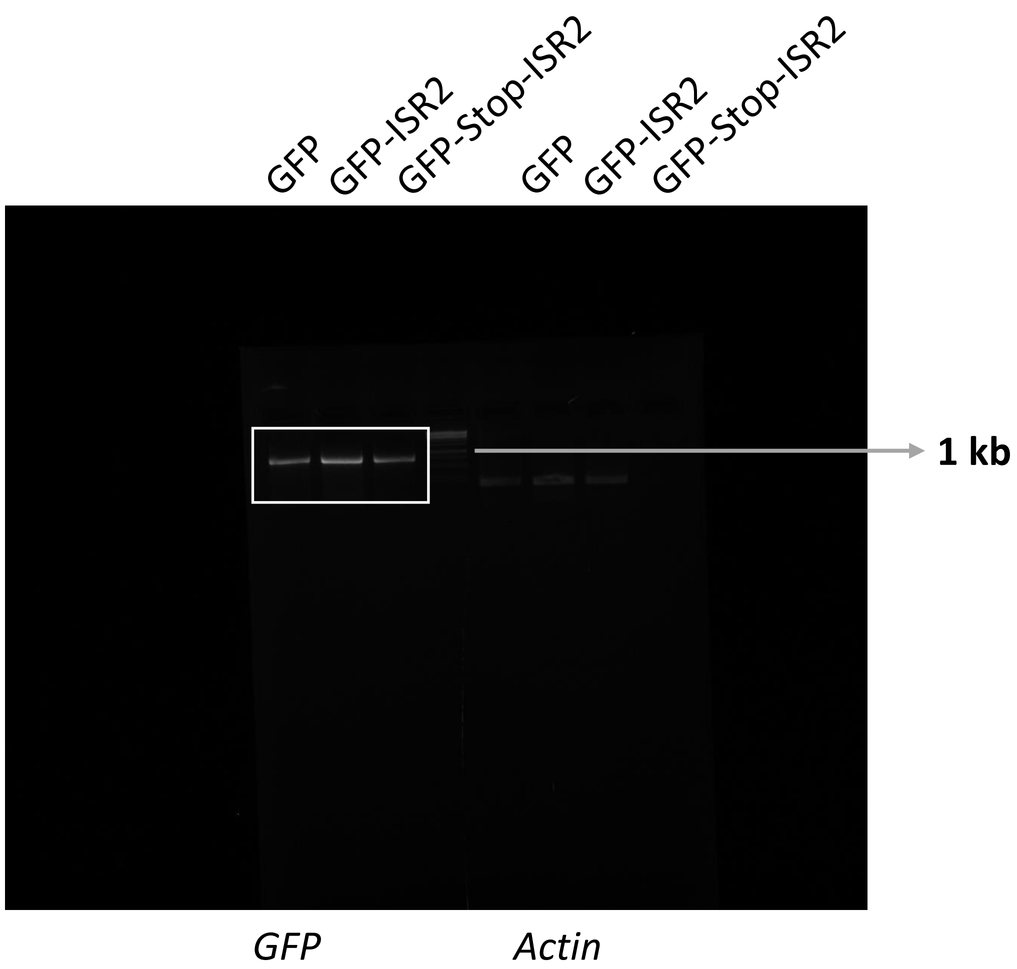

Exposure 1

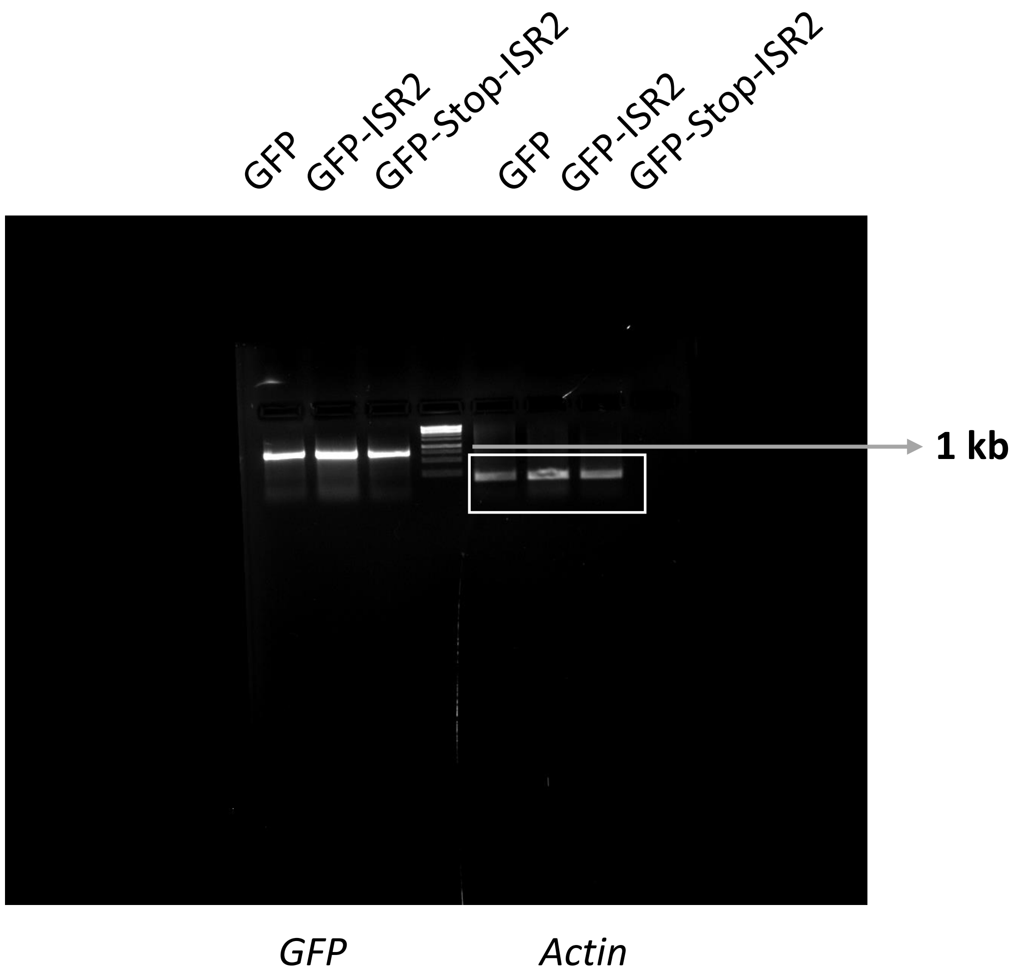

Exposure 2

Fig 6E

Sample	Replicate	%GFP positive cells	Average	SD
**GFP**	1	19.14	18.69666667	0.650410127
	2	19		
	3	17.95		
**GFP-ISR2**	1	2.5	2.05	0.392300905
	2	1.78		
	3	1.87		
**GFP-Stop-ISR2**	1	24.68	20.85333333	3.578412125
	2	17.59		
	3	20.29		
P value				
GFP and GFP-ISR2		<0.001		
GFP-ISR2 and GFP-Stop-ISR2		<0.001		

Fig 6E

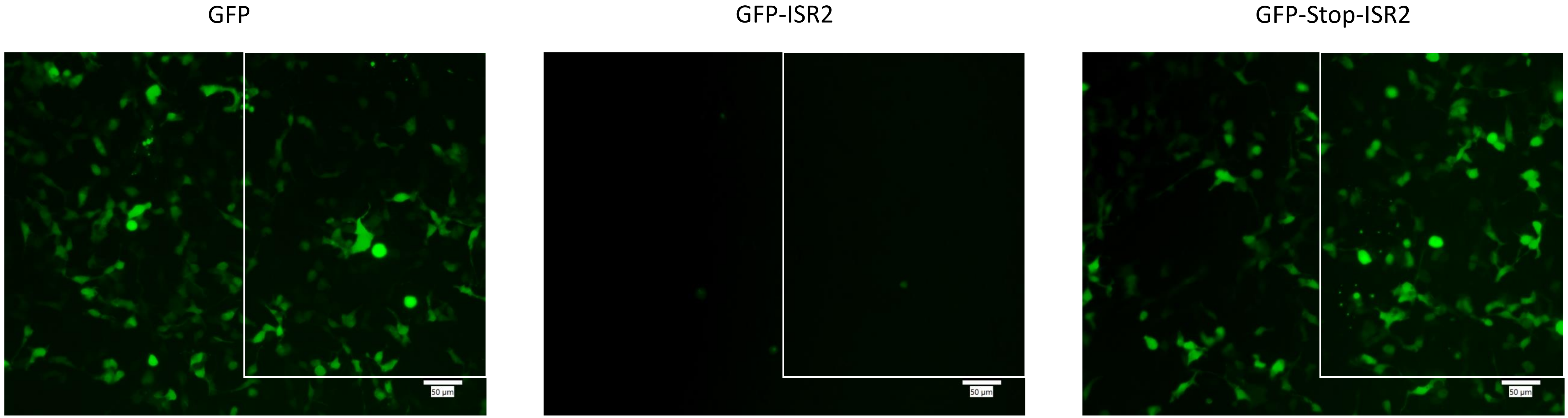

Fig 6F

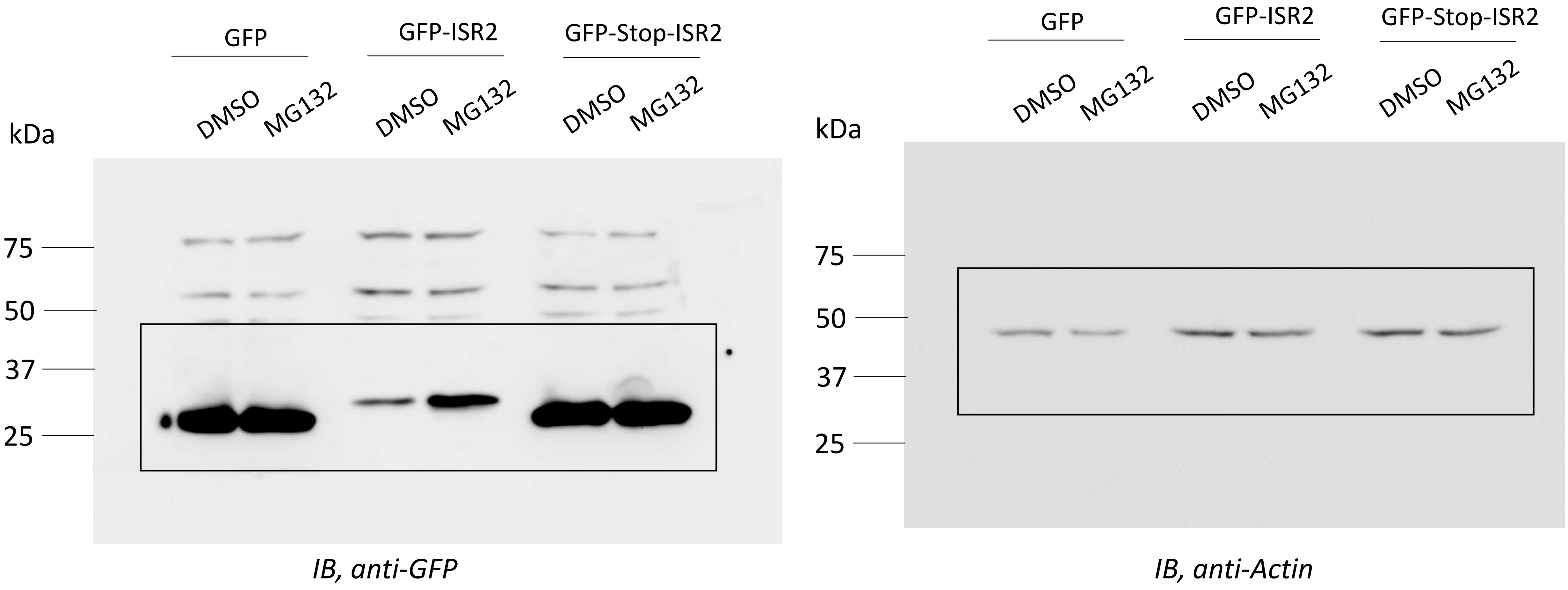
GFP
GFP-ISR2
GFP-Stop-ISR2
DMSO
MG132
DMSO
MG132
DMSO
MG132
kDa
75
50
37
25
IB, anti-GFP
GFP
GFP-ISR2
GFP-Stop-ISR2
DMSO
MG132
DMSO
MG132
DMSO
MG132
kDa
75
50
37
25
IB, anti-Actin

Fig 7A

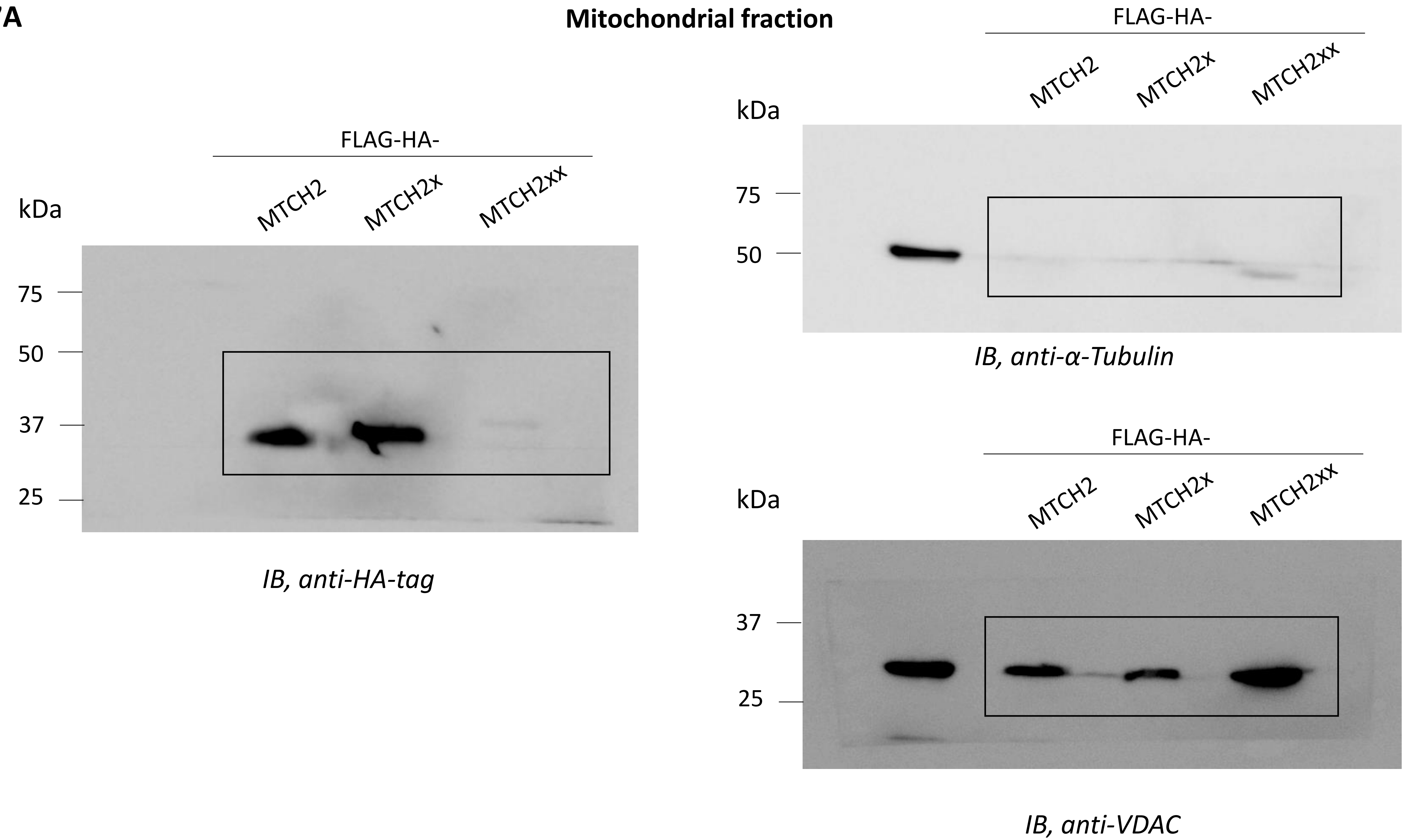

Mitochondrial fraction
FLAG-HA-
MTCH2
MTCH2x
MTCH2xx
kDa
75
50
37
25
IB, anti-HA-tag
FLAG-HA-
MTCH2
MTCH2x
MTCH2xx
kDa
75
50
IB, anti-α-Tubulin
FLAG-HA-
MTCH2
MTCH2x
MTCH2xx
kDa
37
25
IB, anti-VDAC

Fig 7A

Cytosolic fraction

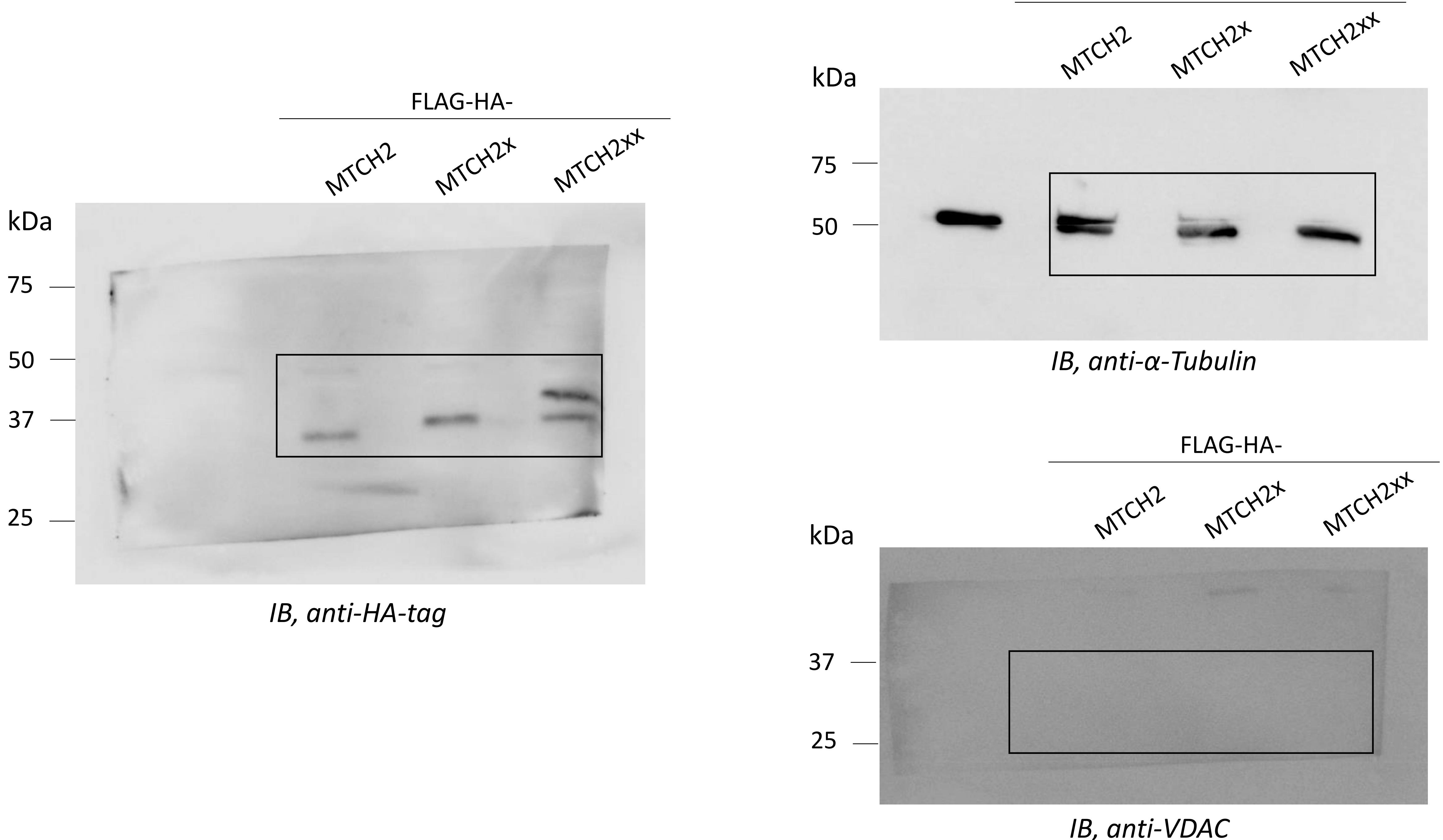

Fig 7B

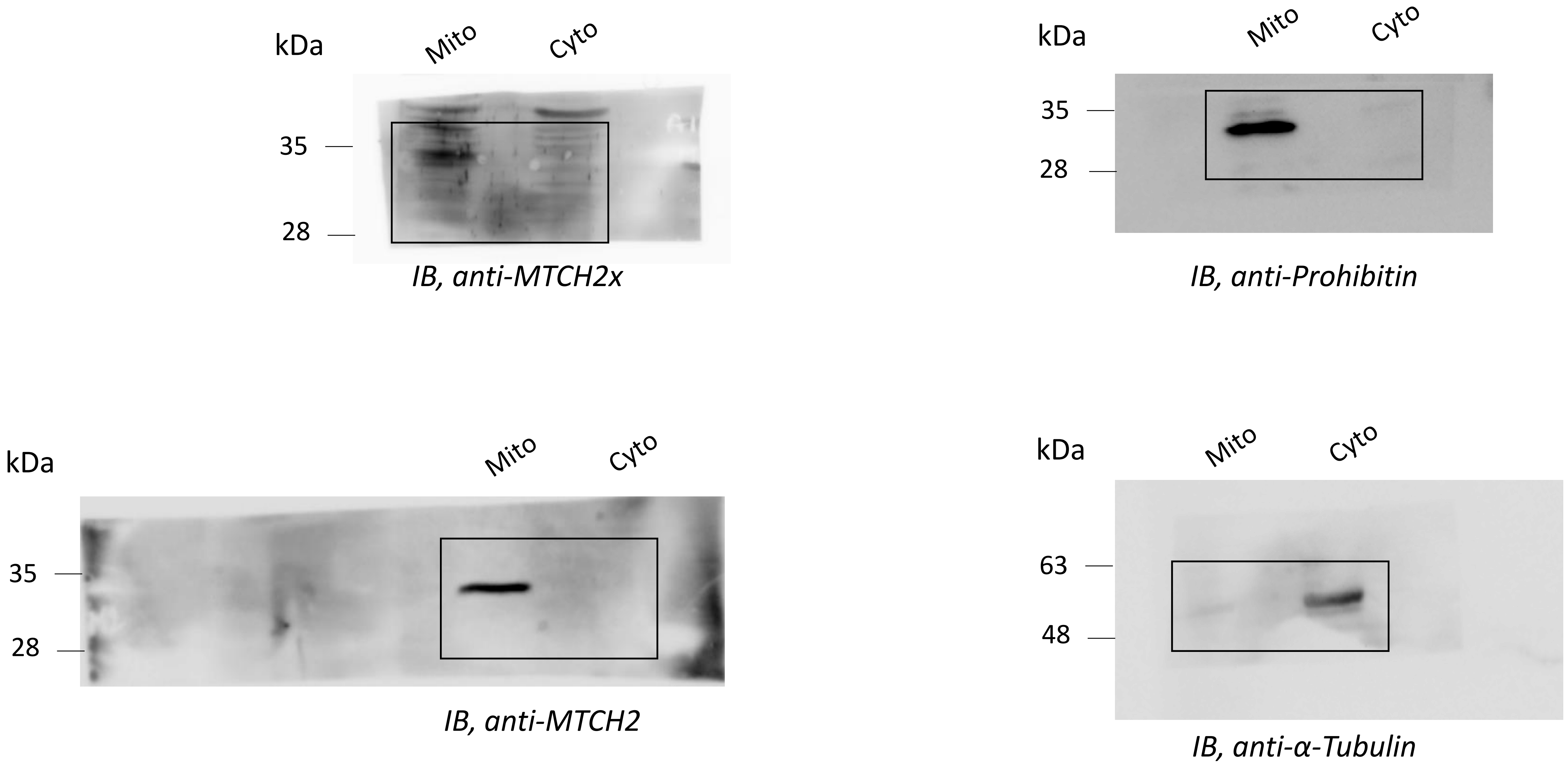
kDa
Mito
Cyto
35
28
IB, anti-MTCH2x
kDa
Mito
Cyto
35
28
IB, anti-Prohibitin
kDa
Mito
Cyto
35
28
IB, anti-MTCH2
kDa
Mito
Cyto
63
48
IB, anti-α-Tubulin

Fig 7C

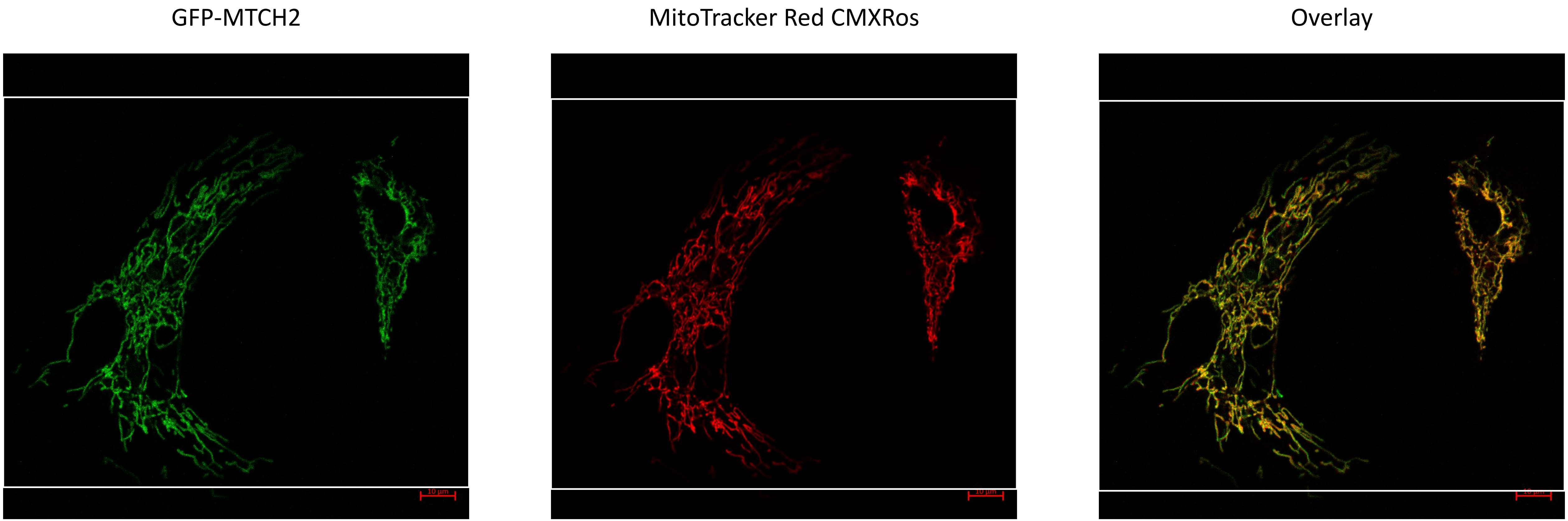

Fig 7C

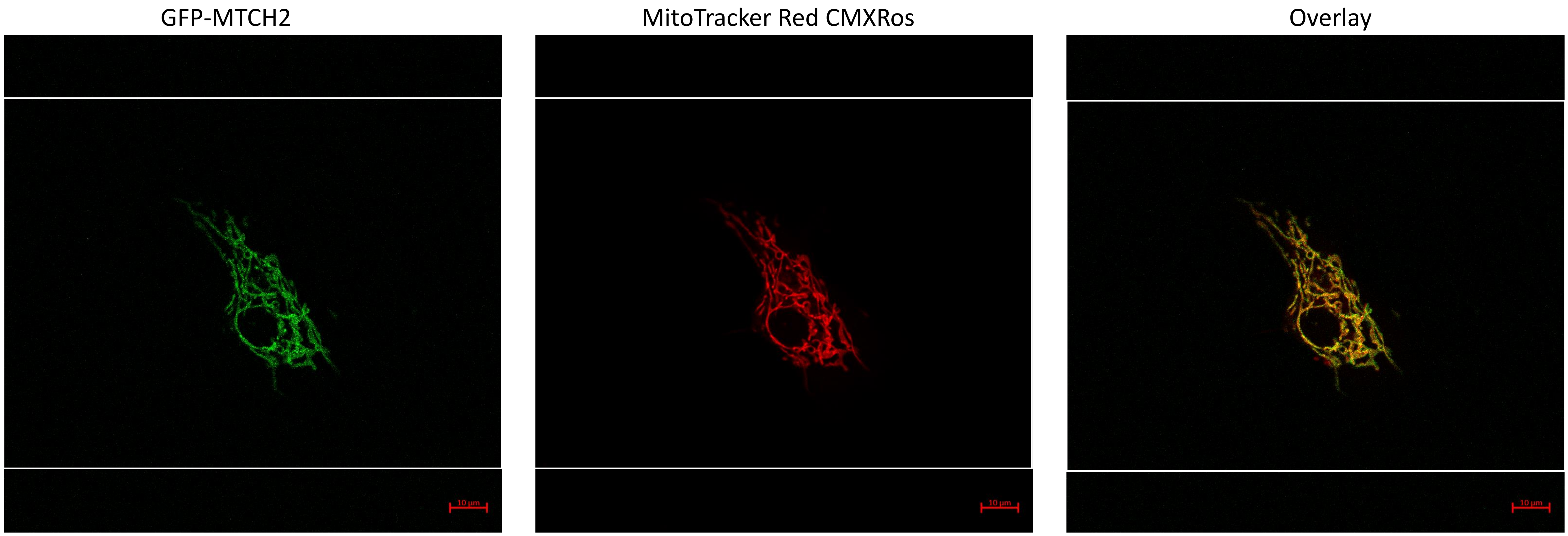

Fig 7C

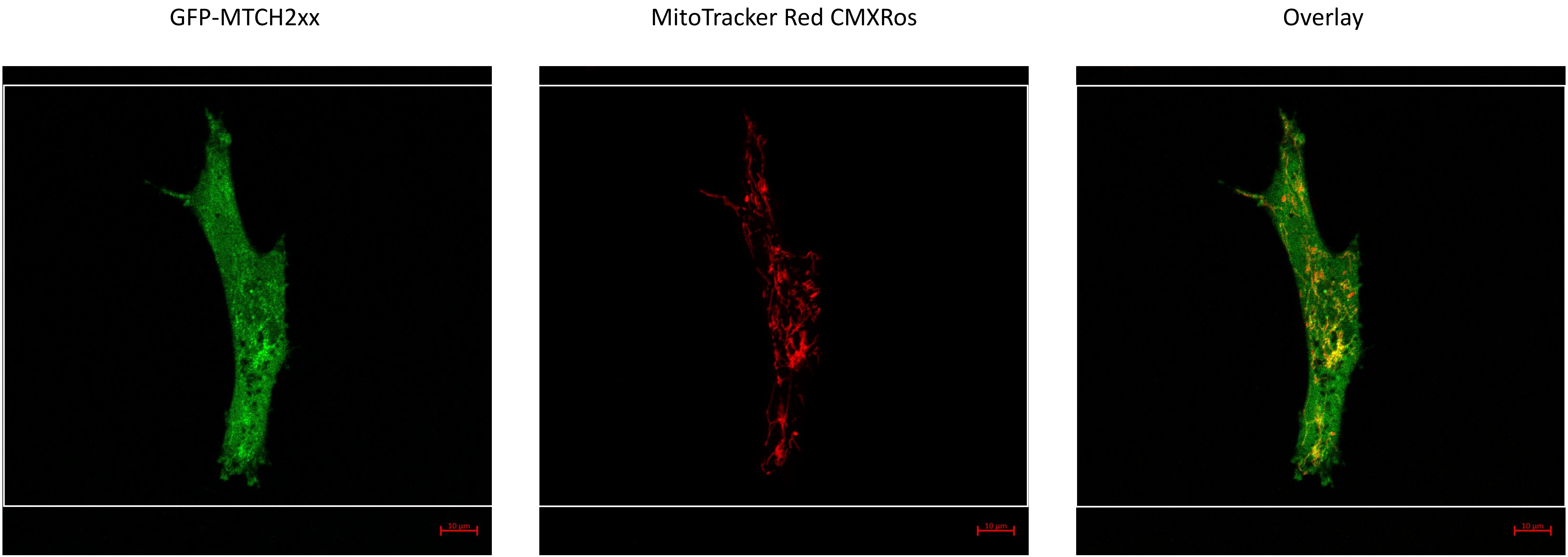

Fig 8A

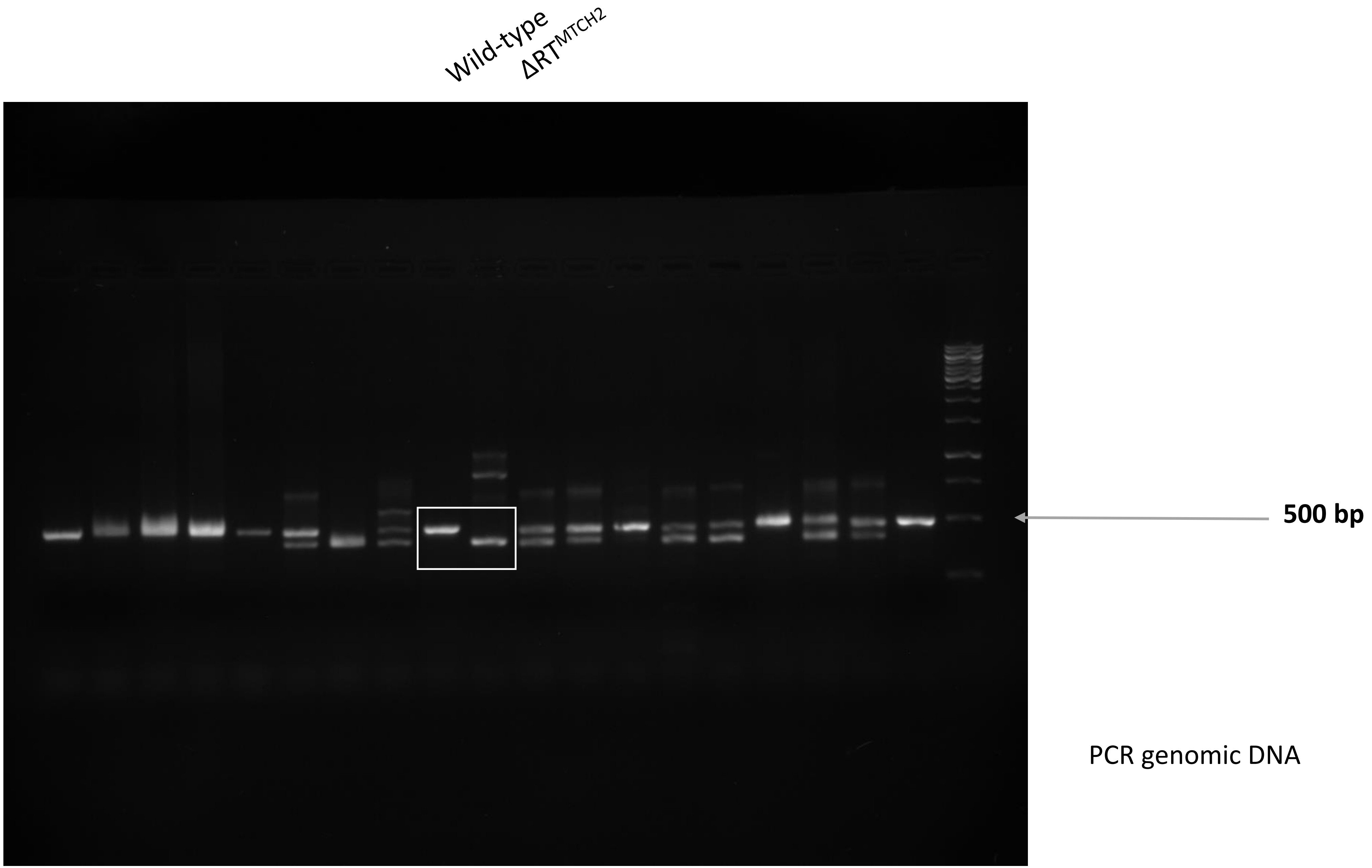

Fig 8B

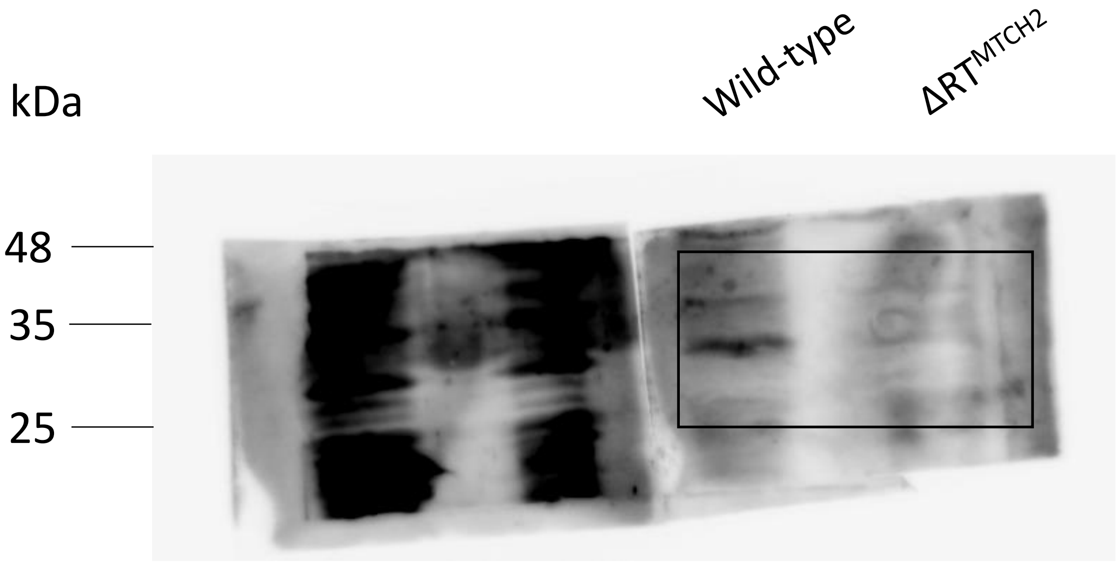

IB, anti-MTCH2x

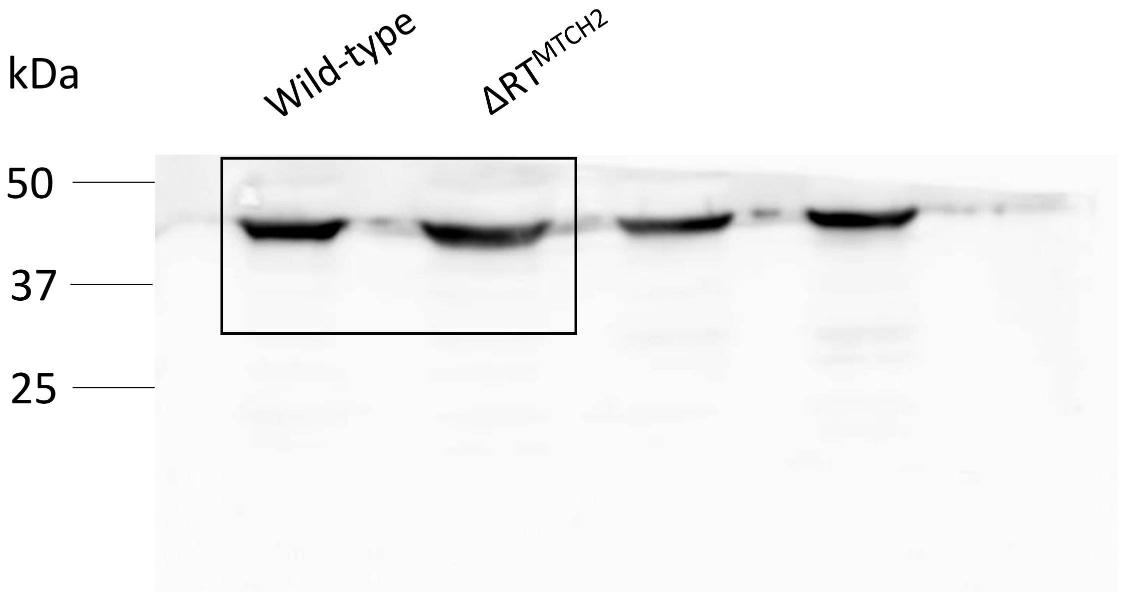

IB, anti-Actin

Fig 8B

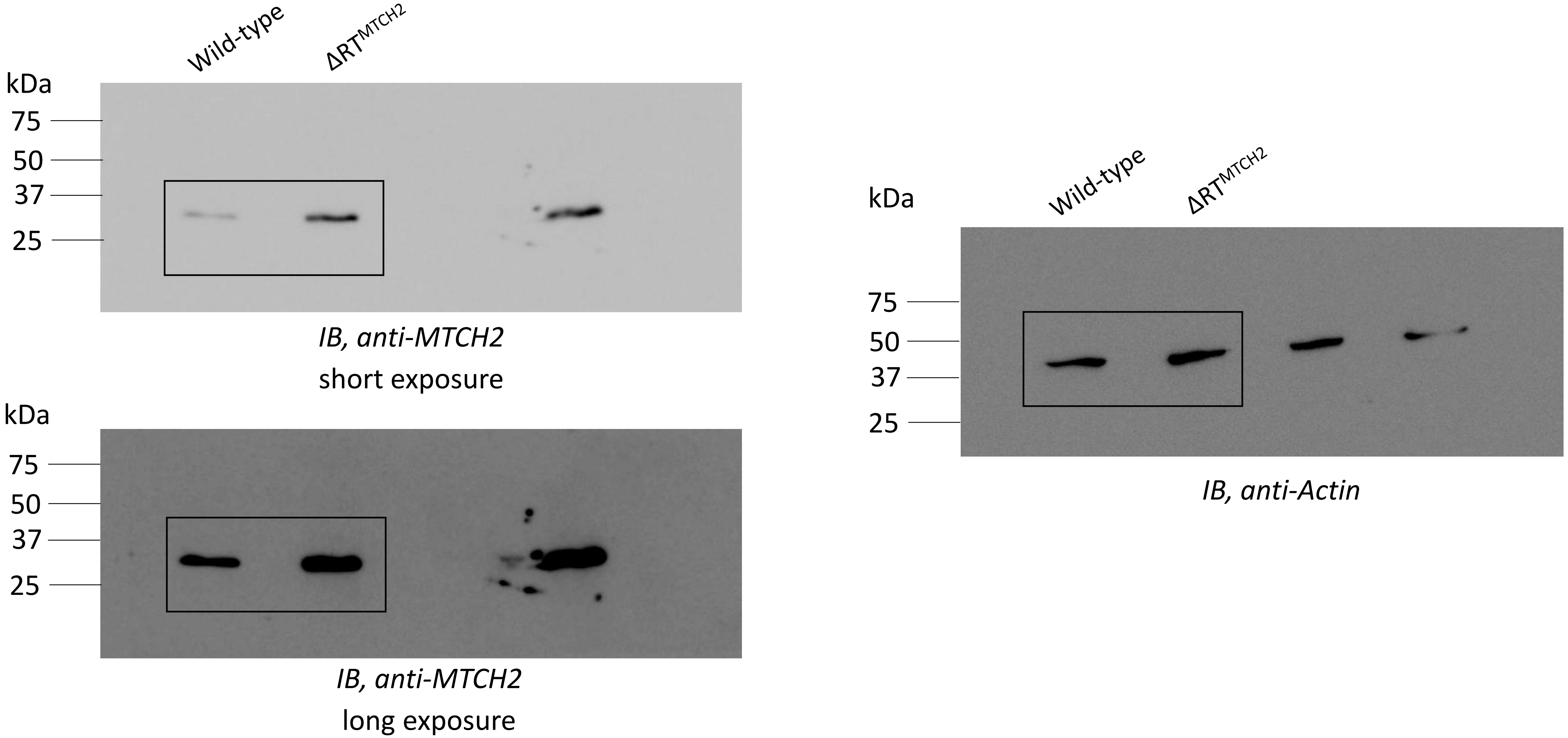
Wild-type
ΔRT^MTCH2
kDa
75
50
37
25
IB, anti-MTCH2
short exposure
kDa
75
50
37
25
IB, anti-MTCH2
long exposure
Wild-type
ΔRT^MTCH2
kDa
75
50
37
25
IB, anti-Actin

Fig 8C

Sample	Replicate	Fluorescence ratio $\lambda_{594}/\lambda_{538}$	Average	SD
**WT**	1	2.115625	1.866452543	0.197505509
	2	1.749457701		
	3	1.672858617		
	4	1.927868852		
**ΔRT^{MTCH2} Clone1**	1	0.875258442	1.115097812	0.182050791
	2	1.127009646		
	3	1.140215716		
	4	1.317907445		
**ΔRT^{MTCH2} Clone2**	1	1.164420485	1.237301775	0.093652709
	2	1.150819672		
	3	1.337272727		
	4	1.296694215		
**H2O2**	1	0.953448276		
P value				
WT and ΔRT^{MTCH2} Clone1		0.001		
WT and ΔRT^{MTCH2} Clone2		0.001		

Fig 8D

Sample	Replicate	Luminescence	Average	SD
**WT**	1	1190000	1216666.667	83266.63998
	2	1310000		
	3	1150000		
**ΔRT^{MTCH2}**	1	874000	871333.3333	95028.06603
	2	965000		
	3	775000		
P value				
WT and ΔRT^{MTCH2}		0.009		

Fig 8E

Sample	Replicate	Mean fluorescence	Average	SD
**WT**	1	5911.62	5932.093333	461.6006452
	2	5481.07		
	3	6403.59		
**ΔRT^{MTCH2}**	1	6175.87	6319.166667	126.5572267
	2	6365.99		
	3	6415.64		
P value				
WT and ΔRT^{MTCH2}		ns		

Fig 8F

Sample	Replicate	Expression in ΔRT^{MTCH2} relative to WT	Average	SD
MT-ND1	1	0.271683716	0.306139678	0.032727104
	2	0.336808394		
	3	0.309926925		
MT-CYB	1	0.287174589	0.285195496	0.001976813
	2	0.285190929		
	3	0.283220971		
MT-CO1	1	0.305660069	0.346787207	0.063936831
	2	0.420448208		
	3	0.314253344		
MT-ATP6	1	0.236514412	0.321583023	0.087753691
	2	0.316439148		
	3	0.411795509		
P value				
WT and ΔRT^{MTCH2}		<0.01		

Fig 8G

		Day 0			Day 1			Day 2			Day 3		
**Sample**	**Replicate**	**Fold proliferation**	**Average**	**SD**	**Fold proliferation**	**Average**	**SD**	**Fold proliferation**	**Average**	**SD**	**Fold proliferation**	**Average**	**SD**
**WT**	1	1.073634204	1	0.0493	5.035629454	4.81473	0.2185	8.019002375	9.16152	0.792	10.69833729	10.1235	0.4614
	2	0.978622328			4.864608076			9.843230404			10.26128266		
	3	0.978622328			4.845605701			9.339667458			9.90023753		
	4	0.96912114			4.513064133			9.444180523			9.634204276		
**ΔRT^{MTCH2} Clone1**	1	0.95	1	0.0688	3.43	3.8675	0.3042	7.15	6.4075	0.5886	7.56	6.595	0.8941
	2	0.99			3.9			5.92			7.15		
	3	1.1			4.11			6.61			5.79		
	4	0.96			4.03			5.95			5.88		
**ΔRT^{MTCH2} Clone2**	1	0.974169742	1	0.0484	4.273062731	4.31365	0.1968	4.265682657	4.76937	1.059	3.143911439	3.27491	0.549
	2	0.95202952			4.472324723			4.472324723			3.741697417		
	3	1.011070111			4.457564576			4.007380074			3.660516605		
	4	1.062730627			4.051660517			6.332103321			2.553505535		
P value													
WT and ΔRT^{MTCH2} Clone1		<0.0001											
WT and ΔRT^{MTCH2} Clone2		<0.0001											

Fig 8G

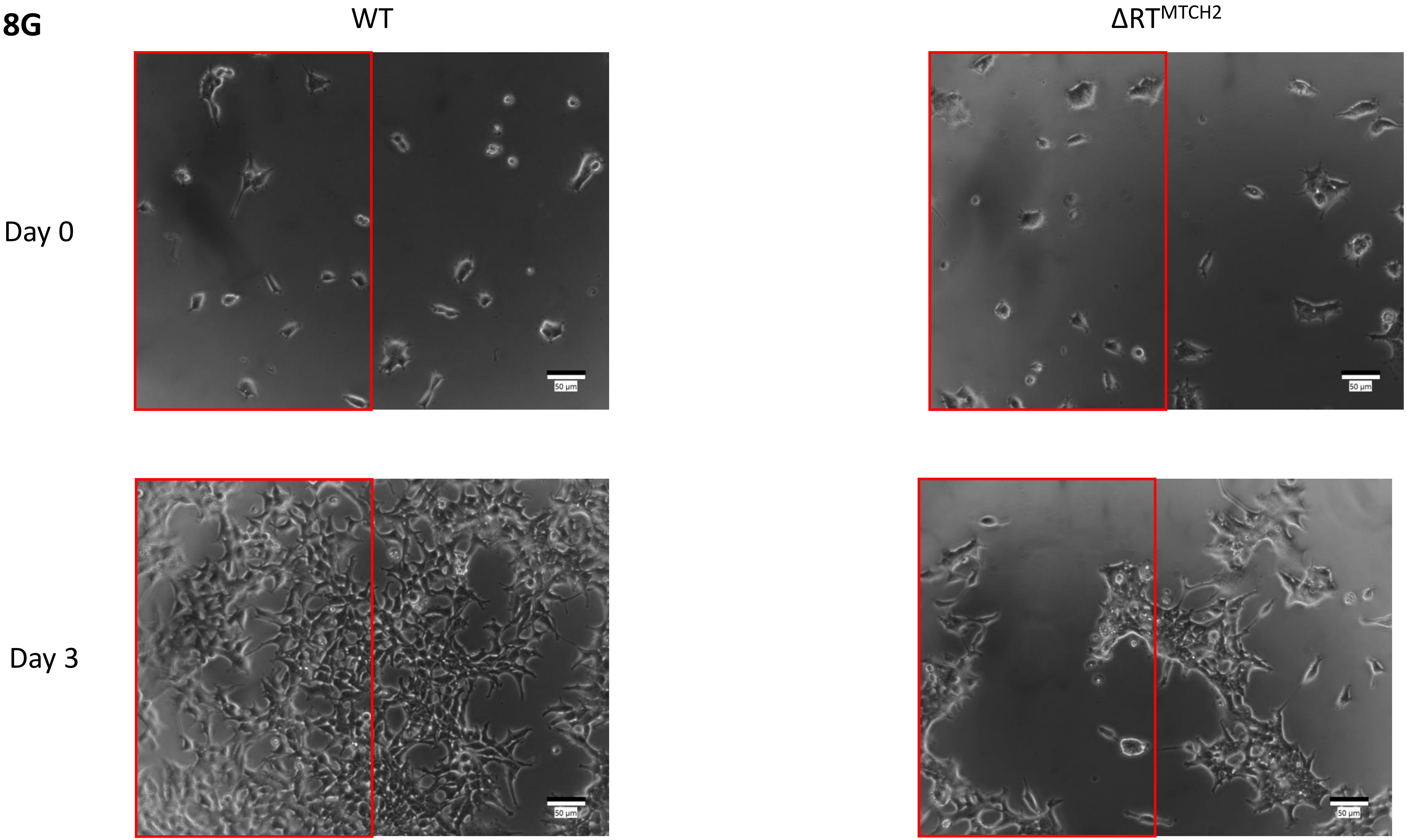

Fig S3

Sample	Replicate	Fluc	Rluc	Fluc/Rluc	Avg	SD
MTCH2* -Stop-*Fluc	1	98	940	0.104255319	0.089402752	0.025513265
	2	332	3192	0.104010025		
	3	63	1051	0.059942912		
MTCH2* -Stop-ISR1-*FLuc	1	1061	4498	0.235882614	0.237692587	0.03628877
	2	1211	4406	0.274852474		
	3	881	4354	0.202342673		
MTCH2* -Stop-ISR1+12^{ISR2}-*FLuc	1	28155	10658	2.641677613	2.253733598	0.346227203
	2	27455	12809	2.143414786		
	3	24648	12473	1.976108394		
MTCH2* -No Stop-*Fluc	1	51825	12858	4.030564629	4.207588089	0.163085407
	2	47981	11315	4.240477243		
	3	51668	11873	4.351722395		
P value						
MTCH2- Stop-*Fluc* and *MTCH2-* Stop-ISR1-*FLuc*		0.005				
MTCH2- Stop-*Fluc* and *MTCH2-* Stop-ISR1+12^{ISR2}-*FLuc*		0.0004				

Fig S3

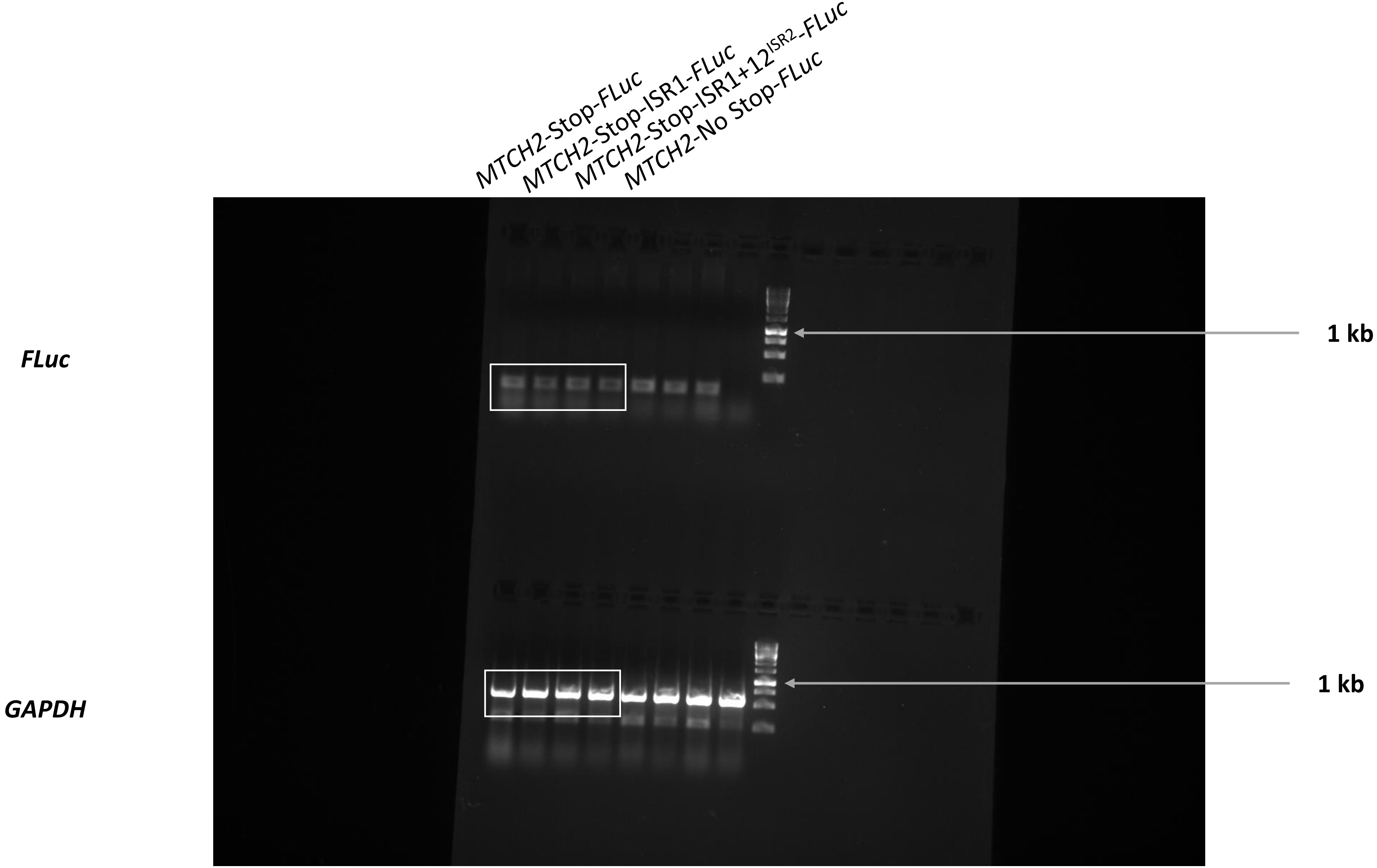
MTCH2-Stop-FLuc
MTCH2-Stop-ISR1-FLuc
MTCH2-Stop-ISR1+12ISR2-FLuc
MTCH2-No Stop-FLuc
FLuc
GAPDH
1 kb
1 kb

Fig S4

Sample	Replicate	Fluc	Avg	SD
**MTCH2-Stop-Fluc**	1	35	36	6.557438524
	2	43		
	3	30		
**MTCH2-Stop-ISR1+12^{ISR2} (WT)-FLuc**	1	2110	1990	355.5277767
	2	2270		
	3	1590		
**MTCH2-Stop-ISR1+12^{ISR2} (GG-CU)-FLuc**	1	2090	1873.333333	188.2374387
	2	1750		
	3	1780		
**MTCH2-Stop-ISR1+12^{ISR2} (AG-GA)-FLuc**	1	2860	2923.333333	290.229794
	2	3240		
	3	2670		

Fig S5A

Sample	Replicate	Fluc	Avg	SD
**MTCH2-Stop-Fluc**	1	18	17.33333333	1.154700538
	2	18		
	3	16		
**MTCH2-Stop-ISR1+12^{ISR2}-FLuc**	1	924	1132	497.7388874
	2	1700		
	3	772		
**MTCH2-Stop-12^{ISR2}-FLuc**	1	1050	1002.333333	118.8963134
	2	1090		
	3	867		
**MTCH2-non specific-FLuc**	1	40	39.33333333	1.154700538
	2	40		
	3	38		
P value				
MTCH2 -Stop-*Fluc* and *MTCH2* -Stop-ISR1+12^{ISR2}-*FLuc*		0.018		
MTCH2 -Stop-*Fluc* and *MTCH2* -Stop+12^{ISR2}-*FLuc*		<0.001		
MTCH2 -Stop+12^{ISR2}-*Fluc* and *MTCH2* -Non specific-*Fluc*		<0.001		

Fig S5B

Sample	Replicate	Fluc	Avg	SD
**MTCH2-Stop-ISR1-Stop-Fluc**	1	135	164.6666667	29.0229794
	2	166		
	3	193		
**MTCH2-Stop-ISR1-Stop-12^{ISR2}-FLuc**	1	18000	23333.33333	5401.234427
	2	28800		
	3	23200		
P value	0.018	Welch's correction applied		

Fig S6A

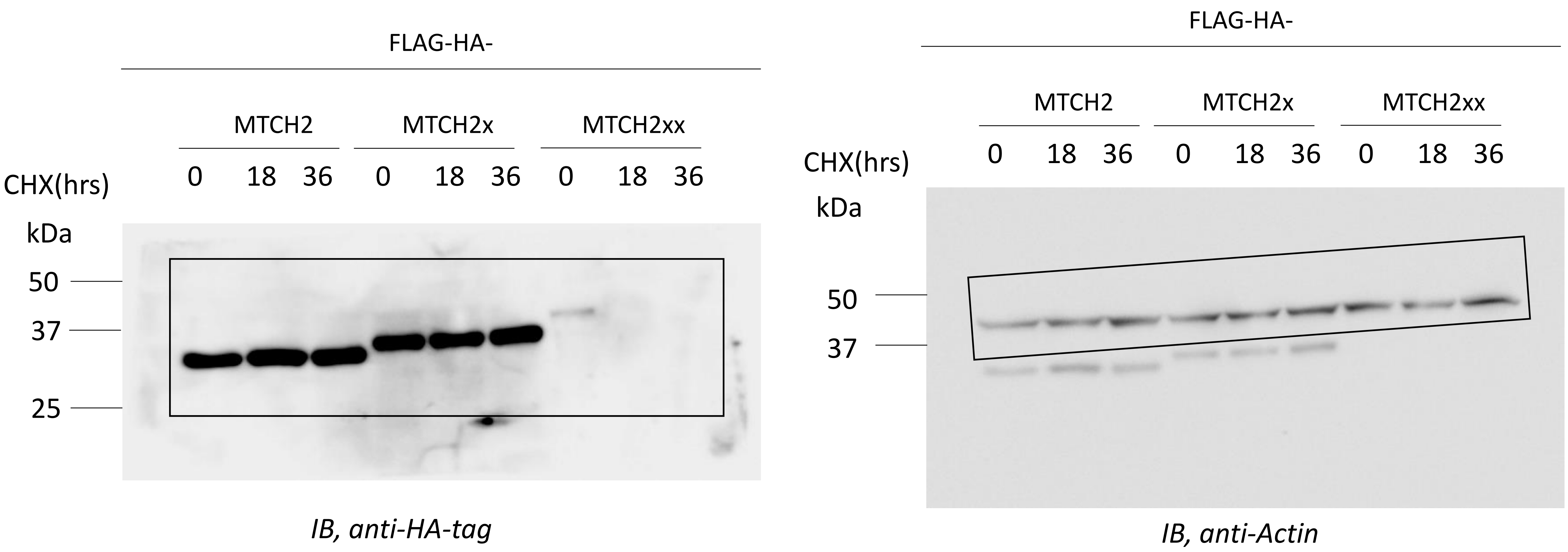
FLAG-HA-
MTCH2
MTCH2x
MTCH2xx
CHX(hrs)
0
18
36
0
18
36
0
18
36
kDa
50
37
25
IB, anti-HA-tag
FLAG-HA-
MTCH2
MTCH2x
MTCH2xx
CHX(hrs)
0
18
36
0
18
36
0
18
36
kDa
50
37
IB, anti-Actin

Fig S6B

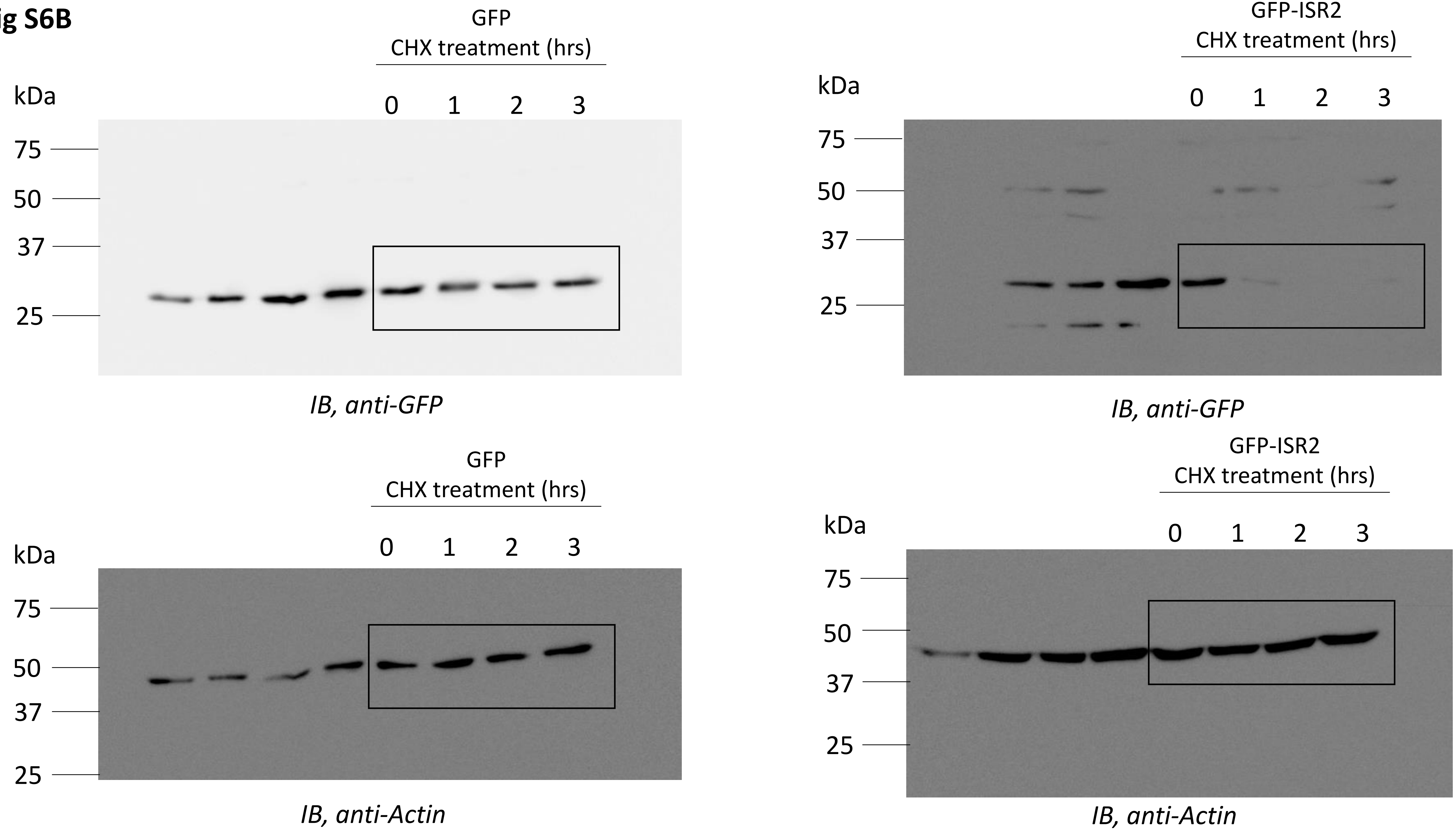
GFP
CHX treatment (hrs)
kDa
0 1 2 3
75
50
37
25
IB, anti-GFP
GFP-ISR2
CHX treatment (hrs)
kDa
0 1 2 3
75
50
37
25
IB, anti-GFP
GFP
CHX treatment (hrs)
kDa
0 1 2 3
75
50
37
25
IB, anti-Actin
GFP-ISR2
CHX treatment (hrs)
kDa
0 1 2 3
75
50
37
25
IB, anti-Actin

Fig S6C

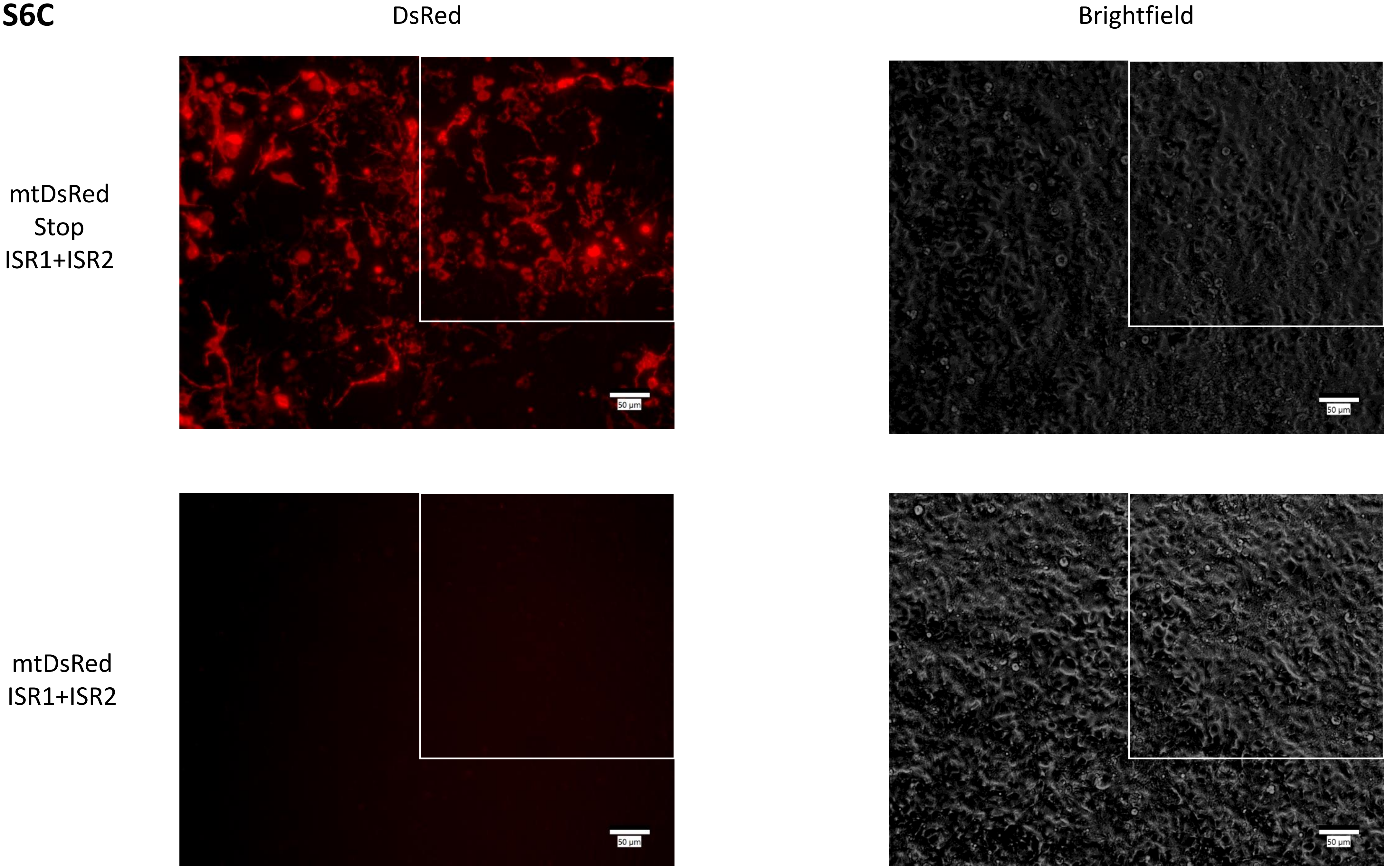
DsRed
Brightfield
mtDsRed
Stop
ISR1+ISR2
mtDsRed
ISR1+ISR2
50 µm
50 µm
50 µm
50 µm

Fig S6C

mtDsRed

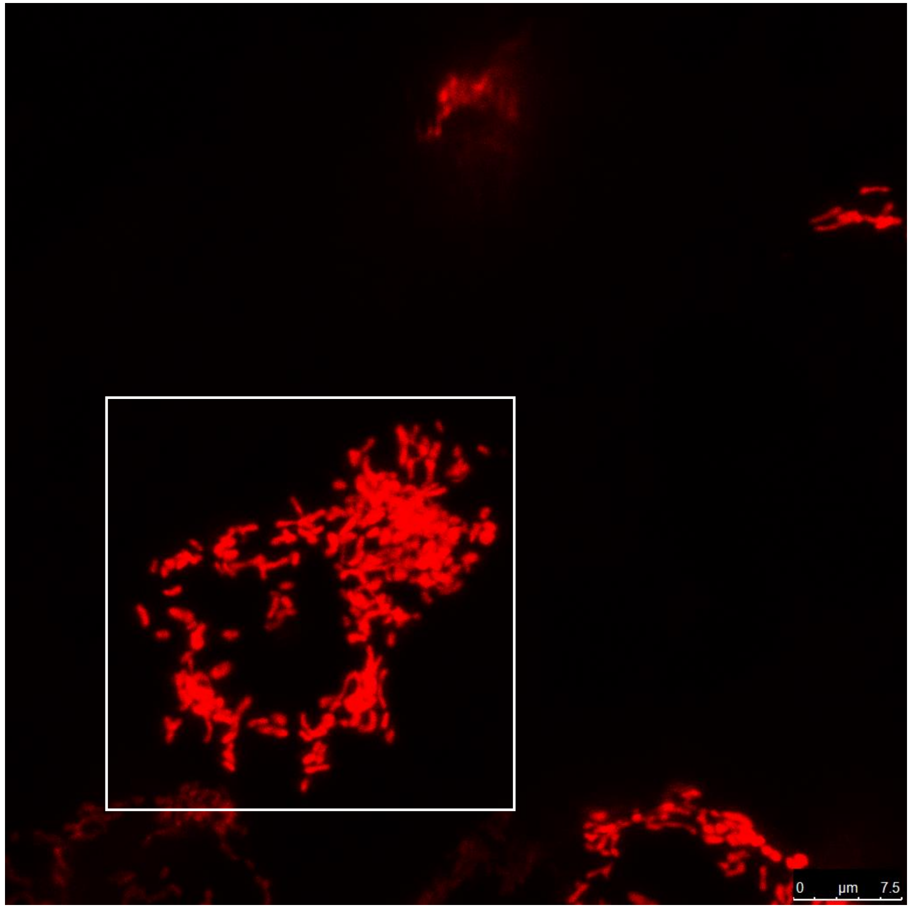

HEK293

Fig S7A

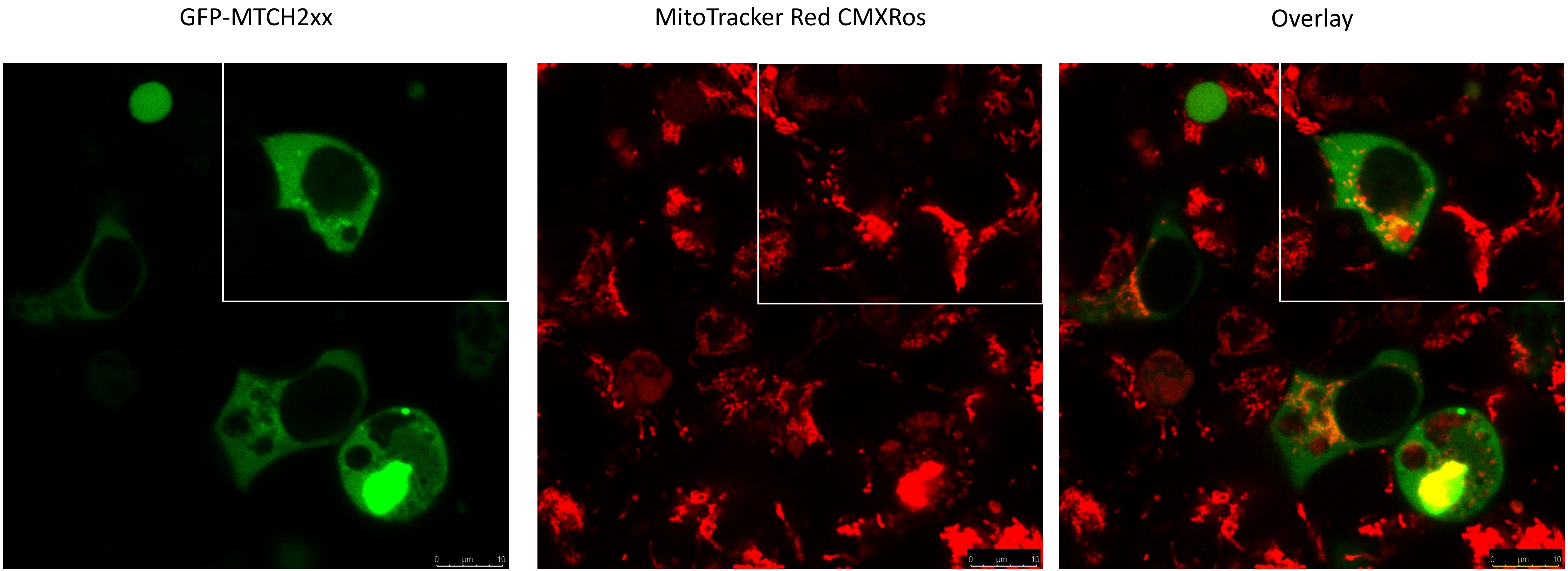
GFP-MTCH2xx
MitoTracker Red CMXRos
Overlay
0 µm 10
0 µm 10
0 µm 10

Fig S7A

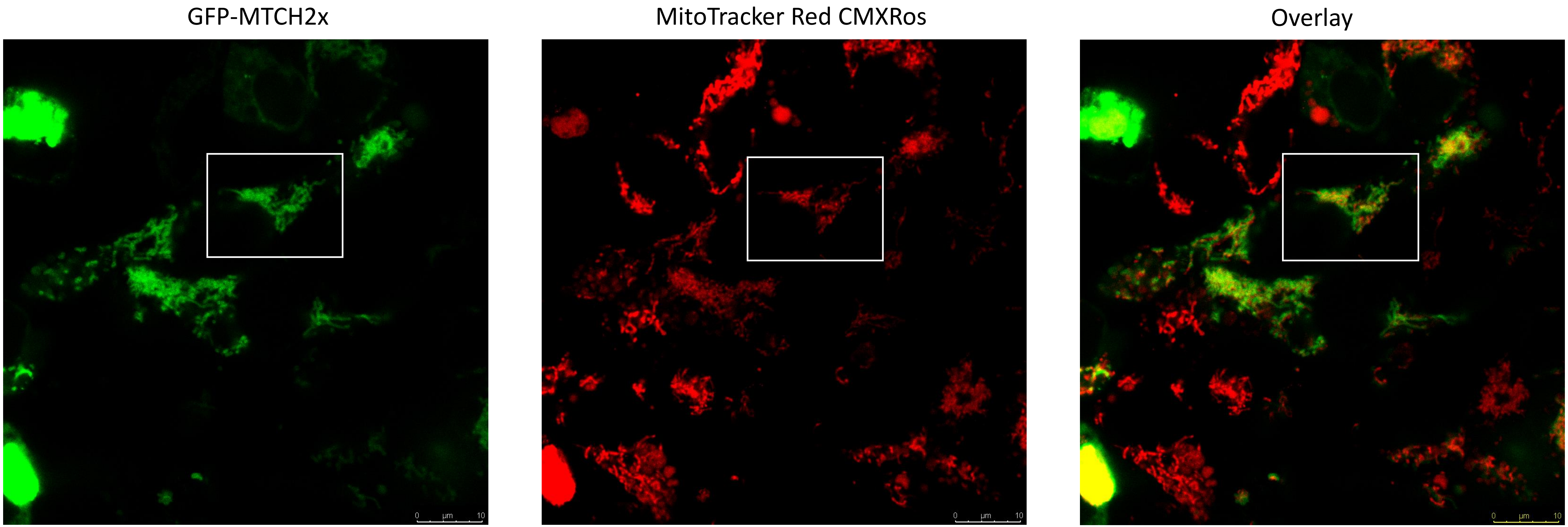
GFP-MTCH2x
MitoTracker Red CMXRos
Overlay
0 µm 10
0 µm 10
0 µm 10

Fig S7A

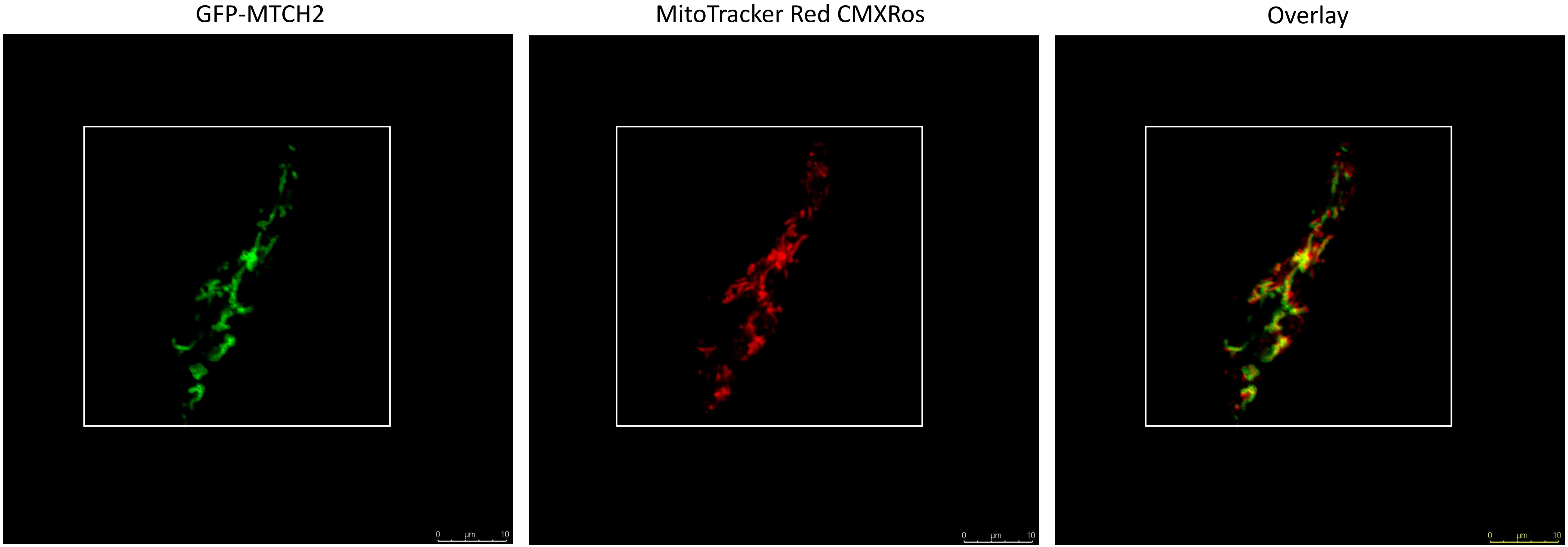
GFP-MTCH2
MitoTracker Red CMXRos
Overlay
0 µm 10
0 µm 10
0 µm 10

Fig S7B

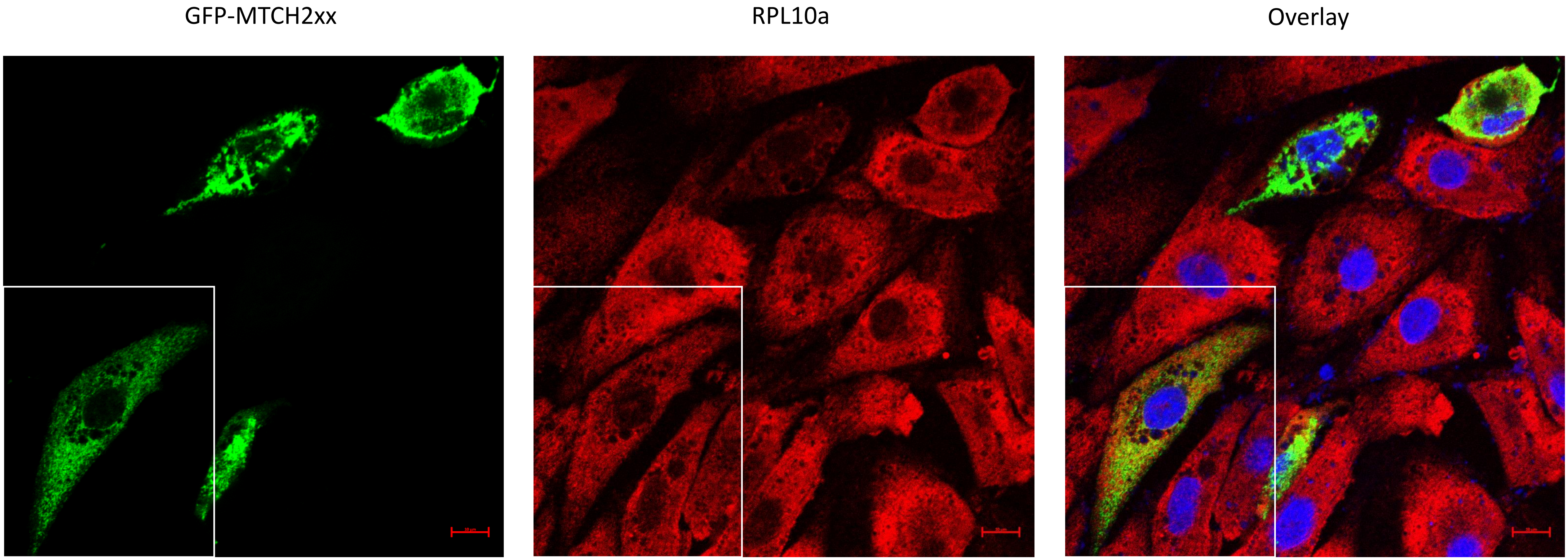

Supplement: Supporting Information [file supp_RA120.014253_160699_2_supp_606529_qhl9hf.pdf]
